# A neural signature of adaptive mentalization

**DOI:** 10.1038/s41593-026-02219-x

**Published:** 2026-03-09

**Authors:** Niklas Buergi, Gökhan Aydogan, Arkady Konovalov, Christian C. Ruff

**Affiliations:** 1 Zurich Center for Neuroeconomics, Department of Economics, University of Zurich, Zurich, Switzerland; 2 Max Planck Institute for Biological Cybernetics, Tübingen, Germany; 3 University Research Priority Program ‘Adaptive Brain Circuits in Development and Learning’ (URPP AdaBD), University of Zurich, Zurich, Switzerland; 4 School of Psychology, Centre for Human Brain Health, University of Birmingham, Birmingham, UK; 5 Faculty of Medicine, University of Zurich, Zurich, Switzerland

**Keywords:** Neural decoding, Learning algorithms, Social behaviour, Psychology, Decision

## Abstract

Mentalization, inferring others’ emotions and intentions, is crucial for human social interactions and is impaired in various brain disorders. While previous neuroscience research has focused on static mentalization strategies, we know little about how the brain adaptively selects which strategies to use at any given moment. Here we investigate this core aspect of mentalization with computational modeling and functional magnetic resonance imaging (fMRI) during interactive strategic games. We find that most participants can adapt their strategies to the changing sophistication of their opponents, though there are considerable individual differences. Model-based fMRI analyses identify a distributed brain network in which activity and connectivity track this mentalization-belief adaptation. The extent to which people update their beliefs about others’ sophistication can be predicted out of sample from neural activity, providing a neural signature of adaptive mentalization. Our model elucidates the neural basis of mentalization ability and provides a method for assessing these capabilities in healthy and clinical populations.

## Abstract (web summary)

Successful social interaction relies on predicting others’ behavior. Buergi et al. combined computational modeling and brain imaging to show how humans adjust to changing strategies, revealing a neural signature of adaptive belief updating.

## Main

Mentalization—the ability to infer beliefs, desires and emotions of others—is essential in virtually all human interactions, from interpersonal relationships to navigating political and business landscapes^1,2^. Recursive thought processes (for example, ‘I think that you think that I think’) have a critical role in language^3,4^ and lie at the heart of both cooperative^5^ and noncooperative social interactions^5,6^. Previous studies have developed paradigms to assess the ability to mentalize^7–11^, providing measures of specific mentalization strategies that can be compared across people^11^ and that help to identify patterns of brain activity in the social brain network^11,12^ that are correlationally^13^ and even causally^11^ linked to these strategies^4,7^.

Several studies have proposed that the temporoparietal junction (TPJ) may serve as a hub for cognition related to mentalization^7,11,14^. However, TPJ activation per se is not specific to mentalization and therefore cannot be used to infer whether people engage in mentalizing^14^. Moreover, tasks that measure the application of generic, context-independent mentalizing strategies (such as false belief tasks) do not capture the constantly changing behavioral strategies (and therefore mental states) of social interaction partners^15^. Experimental measures of static mentalization strategies may thus miss a key aspect of real-life mentalization. In line with this notion, recent findings have questioned the external validity of such accounts^8,15^.

Conversely, success in deception strategies may rely on the ability to dynamically adjust one’s reasoning to match a counterpart’s current line of thought^16,17^—a process we refer to as ‘adaptive mentalization’. Studies of repeated interactive games have suggested that humans do not statically apply the same strategy (as assumed in, for example, level-*k* models)^18^ but rather flexibly update their mentalization strategy to match the estimated current level of sophistication of their interaction partners^19–22^. We know very little about the neural processes underlying this key feature of mentalization^23^. Because this ability (that is, addressing ‘how’ to infer the other’s mental processes) operates on a higher conceptual level than the mentalizing processes previous work has investigated (that is, deriving ‘what’ to infer given a specific mental model), it is inherently unclear if similar or different brain regions underlie this ability.

Here we introduce an experimental and computational framework to assess adaptive mentalization and establish the underlying neurocomputational mechanisms. In line with previous behavioral accounts of mentalization^5,19,20^, our framework uses dynamic interactive games to investigate the adaptive nature of mentalization^10,11^. To identify the corresponding neural mechanisms, we developed a computational model (coined ‘Cognitive Hierarchy Assessment’ (CHASE)) that enables us to infer moment-by-moment adaptive changes in mentalization strategy from observed choice behavior. Our model combines learning rules for repeated interactions from cognitive neuroscience^10^ and bounded rationality approaches from behavioral economics (that is, level*-k* thinking)^6^ in an integrated Bayesian framework^23^. In contrast to most previous approaches, our model of adaptive mentalization makes it possible to dynamically track a player’s changing belief about the level of cognitive sophistication of the opponent, which determines the appropriate mentalization strategy to use at any moment. Moreover, the hierarchical structure of this inference process allows us to differentiate adaptive mentalization processes (that is, strategizing) from mere action implementation.

Our approach draws on the idea of iterated reasoning strategies: agents form recursive beliefs about their opponents’ strategies and best respond to the corresponding predictions, an idea established in classic models of strategic reasoning^18,23^. While the classic models assume that agents are endowed with a fixed level of sophistication, newer computational models—like *k*-ToM^20^ and game theory of mind^5,19^—suggest different ways by which agents update beliefs about others’ sophistication. Here we build upon and extend these models (regarding their scope, parsimony and behavioral foundation; Supplementary Note), provide behavioral evidence for the adaptiveness of mentalization and use functional magnetic resonance imaging (fMRI) to isolate neural processes specifically associated with the adaptive component of mentalization. Beyond the univariate approaches used in previous work^5,10^, we applied multivariate machine-learning methods^24^ to decode adaptive mentalization-related belief updates (BUs) from neural activity and replicated these results in an independent, socio-demographically more diverse sample. Crucially, the multivariate pattern allowed us to predict mentalization-related BUs in the replication sample without any retraining, suggesting that it constitutes a general neural signature of adaptive mentalization.

## Results

### Characterizing adaptive mentalization with the CHASE model

**Fig. 1:**
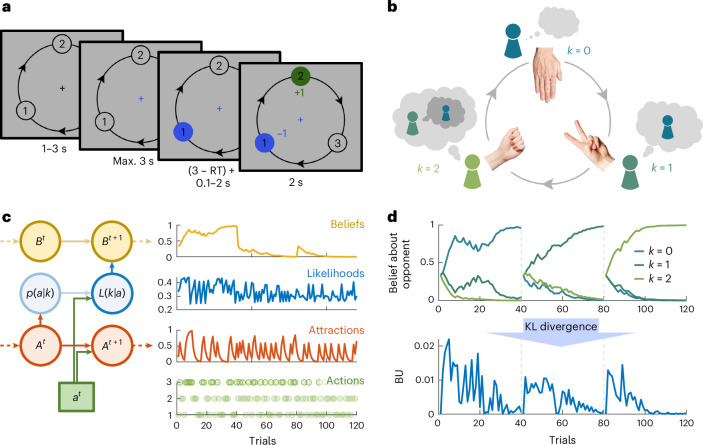
Experimental task and computational model of adaptive mentalization. **a**, Behavioral task. Participants played a repeated RPS game against human or artificial opponents. In each round, both players had to pick a number in private (here from 1, 2 or 3; choice highlighted in blue) before learning about the choice of the other player (in green). The player who picks the action that is exactly one step ahead of the opponent’s action wins the round (in the direction of the arrows), as indicated by numeric feedback. **b**, Illustration of recursive reasoning. If two example players—Sally and Anne—were to play this game, Anne might assume that Sally prefers (and therefore repeats) actions she played in the recent past. If Sally plays paper, Anne thus predicts that Sally will play it again (blue, level-0 strategy). Anne would respond by playing scissors, a level-1 strategy, as it adds one step of reasoning (dark green). However, Sally might be able to anticipate Anne’s thought process and form second-order beliefs (that is, ‘Anne thinks I will play paper given what I picked before, so she will play scissors’), causing Sally to play rock instead (a level-2 strategy; light green). Adaptive mentalization goes beyond predicting behavior given any specific level-strategy, by inferring which of these strategies the opponent is using (**c** and **d**). The hand photos in panel **b** were provided by L.B. Stauffer (University of Illinois Urbana-Champaign). **c**, Graphical model and illustrative time series. The proposed CHASE model captures the adaptive mentalization process of a player based on the following three distinct subprocesses: (1) tracking what a nonstrategic player (that is, level *k* = 0) is likely to play due to recency bias (by updating historical action frequencies referred to here as ‘attractions’), (2) applying recursive reasoning (illustrated in **b**) to these attractions, which leads to a mapping from observed actions to underlying levels (that is, a likelihood function (in blue)), and (3) integrating these likelihoods over time to update beliefs about the sophistication of the opponent (in yellow). All latent variables (in circles) are inferred based on the observed actions (in squares). Arrows indicate the information flow (dotted arrows for Markovian relationship). **d**, Example belief distribution and associated BU. The model provides a precise quantification of the individual’s update on the inferred reasoning process (or level *k*) of the opponent. This opponent-level BU is computed as the difference between the belief distributions at two successive time points (and thus independent of any particular level; formally calculated by using the KL divergence).

The CHASE model that captures adaptive mentalization in dynamic repeated games (here adapted rock-paper-scissors (RPS); Fig. 1a) builds on the fact that, if there is a salient action that a nonstrategic agent is likely to pick, strategic players will try to outsmart each other systematically (Fig. 1b). Please note that the ideal strategy in RPS as prescribed by game theory is to play unpredictably, by choosing all actions with equal probability. However, empirical studies show that humans are not good at producing random sequences^25^, which results in exploitable weaknesses for (nonrandom) strategic opponents and therefore provides clear incentives to mentalize^25,26^. Concretely, individuals can track each other’s past history, form first-order, second-order and higher-order beliefs about the other’s future actions and best respond to these. The number of recursive reasoning steps both players perform is often referred to as level *k* in the literature and defines a strategic player’s sophistication^6,18^.

If participants can mentalize adaptively, rather than simply performing a fixed number of reasoning steps, they form and update beliefs about the sophistication of the other player’s current strategy^5^. Formally, they construct a likelihood function over lower levels of sophistication given observed actions and integrate this evidence over time using Bayes rule (Fig. 1c and Supplementary Fig. 12). This inference process is bounded by their own maximum level of sophistication, which we refer to as $\kappa$ (to distinguish it from the currently played level *k*; for a full description of the model and all parameters, see Methods). Given the distribution of beliefs, we can compute the extent to which individuals update those opponent-level beliefs on any given trial using the Kullback–Leibler (KL) divergence between successive belief distributions (Fig. 1d). Finally, we assume that adaptive agents form an integrated prediction over the most likely next opponent action, weighted by the belief distribution over the opponent’s level, and best respond to this prediction given the shared knowledge about the rules of the game (with a degree of noise).

To validate the CHASE model and its underlying assumptions, we recruited a total of 553 participants (collectively making over 11,000 decisions) who played either against one another or against calibrated artificial opponents across several game variations, resulting in nine substudies (Supplementary Tables 1 and 2). This allowed to systematically investigate adaptive mentalization in strategic interactions and compare our model to existing approaches.

### Participants cannot distinguish between human and artificial opponents

**Fig. 2:**
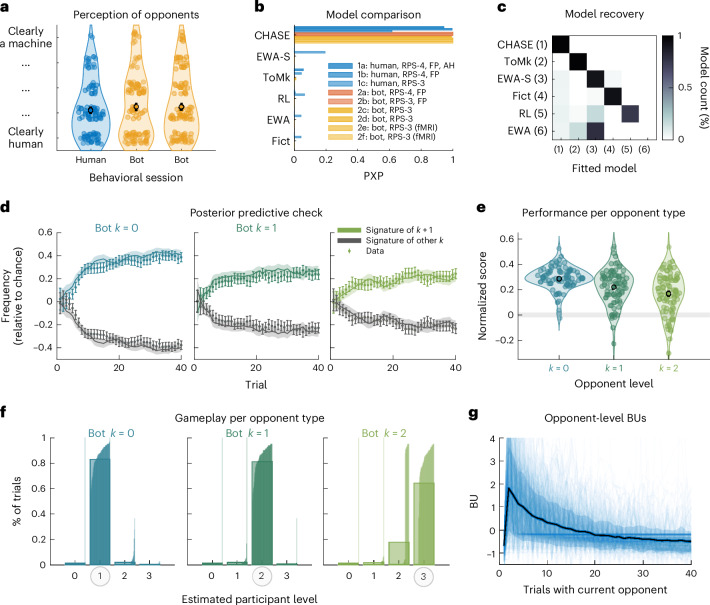
Behavioral and model-based evidence for adaptive mentalization. **a**, Modified Turing test. As some substudies used artificial opponents (based on the CHASE model), we probed participants’ perception of these opponents. When asked to rate the ‘humanness’ of their opponents after six runs of 40 trials, participants’ ratings were not affected by opponent type (*D*(324) = 0.1, *P* = 0.42; two-sided between-participant comparison; datasets 1c and 2d; individual sessions of simultaneous group testing are plotted separately; *n* = 54). **b**, Model comparison. The CHASE model outperforms alternative models of strategic play in explaining participant behavior across nine datasets using RPS-like games (varying if the opponent is human or artificial, if there are three or four actions, if the game is zero-sum or not (FP) and if the AH is displayed or not; PXP for all datasets of >0.85, for pooled data PXP = 1; *n* total = 553). **c**, Confusion matrix from a model recovery analysis that simulates synthetic data with different candidate models (best-fitting parameter estimates from fMRI dataset 2e; *y* axis) and then performs model comparison by plotting proportion of best fit for the corresponding data/model combination. This confirms that the CHASE model makes unique predictions that none of the other models can capture (the only notable confusions occur between EWA variants and RL, but not in any pairing with CHASE in column/row 1). **d**, Posterior predictive check. Simulating synthetic data with CHASE recreates behavioral signatures of adapting to artificial opponents observed in the empirical data (based on parameter estimates and behavior from fMRI dataset 2e, *n* = 50). Plotted is the frequency with which simulated or empirical actions are in line with a stylized best response against a particular opponent relative to chance level (that is, given the correct level and without any noise; Supplementary Methods). Solid lines and shaded areas represent mean and 95% CI of simulated data, error bars represent mean and 95% CI of empirical data (among participants); the frequency of the correct level-response against a particular opponent type is displayed in color and the frequency of alternative strategies is collapsed and displayed in gray. **e**, Performance against the artificial opponents. Participants successfully adapted to the levels of sophistication of their artificial opponents (either *k* = 0, 1 or 2; within participants), as indicated by an overall score (balancing wins and losses) that is significantly above chance (*k* = 0, *t*(85) = 29.01, *P* < 10^−45^; *k* = 1, *t*(85) = 14.71, *P* < 10^−24^; *k* = 2, *t*(85) = 10.09, *P* < 10^−15^; two-sided one-sample *t*-tests; no adjustment for multiple comparisons applied; gray area indicates a 95% CI for the group mean with random gameplay; datasets 2d and 2e; *n* = 86). **f**, Model-inferred levels against the artificial opponents. Most participants played the correct level in response to the different opponents (that is, exactly one level higher). Plotted are the percentage of trials where the gameplay can clearly be assigned to any of the different levels (that is, exceeding a 95% threshold of a permutation distribution), for each of the artificial opponent types (data as in **c**). **g**, While there is substantial variability in the extent to which participants update their beliefs about the level of the opponent at any given point in time, there is a clear decrease over time across participants ($\beta$ = −0.038, *t*(84.4) = −20.6, *P* < 10^−34^; linear mixed-effects model). Plotted are mean (black line), s.d. (blue shaded area) and individual time courses of opponent-level BU within one game (*z* scored within participants). AH, action history; CI, confidence interval.

To ensure that we can detect how people adapt to opponents with different mentalization capabilities, we implemented artificial opponents that provided a ‘ground truth’ for different levels of mentalizing sophistication. To maximize the ecological validity of these artificial agents, we based their behavior on a simplified version of the CHASE model (Methods). As previous work indicated that both behavior and neural activity can change depending on participants’ beliefs about human or artificial agents^5,13,20^, we tested whether participants were able to distinguish human from artificial opponents in a Turing-test-like setting (Methods). Adjusting the agents’ behavior, we ensured the same level of suspicion for human and artificial opponents (overall rating—*D*(54) = 0.14, *P* = 0.96; ratings for individual opponents—*D*(324) = 0.10, *P* = 0.42; datasets 1c and 2d; Fig. 2a), confirming that participants could not distinguish behavioral strategies of human and artificial opponents.

### Participants track action frequencies to predict nonstrategic play

In models of recursive reasoning, a crucial question is what defines the behavior of a level-0 agent, as this is the foundation that all higher levels respond to^6^. To address this, we first empirically validated candidate level-0 strategies by testing whether participants tracked (1) action frequencies, (2) experienced rewards, (3) both experienced and foregone rewards or (4) a combination of action frequencies and rewards. Furthermore, to assess the robustness of the level-0 rule across variations of the RPS game, we conducted nine substudies that varied the possible action space (3 versus 4), memory demands (game history shown), payoff scheme (zero-sum versus nonzero-sum) and whether opponents were human or artificial (Methods; Supplementary Table 1). We first assessed model identifiability through model recovery (Supplementary Fig. 1) and then performed a random-effects Bayesian model comparison between recoverable learning rules. This clearly favored action-frequency tracking across all datasets (pooled protected exceedance probability (PXP) = 1 for both human and artificial opponents; human per dataset = 0.49–0.81; artificial all > 0.99; Supplementary Fig. 2).

### CHASE outperforms existing alternative models

Next, we used these datasets to provide evidence that people adapt to the level of sophistication of their opponents—the crucial feature we can assess with our approach that differentiates it from other models (for example, reinforcement learning (RL), fictitious play (FP)^27^, experience-weighted attraction (EWA)^28^ or influence learning^10^). To this end, we performed random-effects Bayesian model comparison and found strong evidence that the CHASE model provides a better account of participants’ behavior across all the tested game environments (whole-sample PXP = 1.00, artificial opponents PXP = 1.00, human opponents PXP > 0.99, individual datasets all PXP > 0.61; Fig. 2b).

To provide further evidence that the CHASE model is uniquely suited to capture adaptive mentalization, we tested with posterior predictive checks whether the CHASE model, but not the alternative models, is capable of producing qualitative signatures of how participants adapt to different opponents. We simulated synthetic data with all candidate models (based on best-fitting parameter values in the fMRI dataset 2e) and compared the resulting action predictions with the empirically observed actions (in dataset 2e; Supplementary Results). The time course of the qualitative signatures of adaptive mentalization in the simulated data aligned closely with the patterns observed in human data for simulations based on the CHASE model, but not for the alternative models (Fig. 2d and Supplementary Fig. 6). In line with this, a model recovery analysis confirmed that the CHASE model makes unique predictions that no other models can capture (Fig. 2c).

### Participants adapt to the opponent’s level of sophistication

After establishing the CHASE model’s validity, we used it to directly test whether participants could flexibly adapt to varying levels of opponent sophistication (in datasets where participants faced artificial opponents). To ensure balanced exposure to different opponent levels, and to establish a ground truth for model validation, opponent sophistication levels were fixed within blocks of 40 trials (to levels 0, 1 and 2) but varied across blocks. Participants were unaware whether they were facing the same or different opponents across different blocks, requiring them to continuously update their beliefs. We found both model-free and model-based evidence that almost all participants (>95%) were able to adapt, and that the vast majority (~80%) did so successfully across all three levels of sophistication.

The most basic model-free index of successful adaptive mentalization (final score, defined as wins minus losses normalized by trials) was above chance for all opponent levels (*t*(85) > 10.0, *P* < 10^−15^; Fig. 2e) but declined with opponent sophistication ($\beta$ = −0.059, *t*(113.3) = −6.24, *P* < 10^−8^). This is consistent with the greater difficulty of performing more recursive reasoning steps for more sophisticated opponents.

Successful adaptive mentalization was also reflected in the qualitative features we used to index adaptive mentalization in model simulations and data (Fig. 2d–f). Posterior predictive checks (Fig. 2d) revealed within-block increases in the use of the strategy that was optimal against the current opponent and decreases in the use of nonoptimal strategies (Supplementary Fig. 6).

In line with this, when investigating the parameter estimates and inferred beliefs from the fitted model, we found a maximum-sophistication level ($\kappa$) of 3 in 79% of our participants, indicating that almost all participants were able to adapt to an opponent with *k* = 2 (see Supplementary Figs. 13 and 14 for details on parameter estimates). Similarly, the vast majority (~78%) of participants were able to infer the correct level of their respective opponents during the interaction (based on a threshold of *P* < 0.05; Fig. 2f). While this relatively high proportion contrasts with findings about recursive reasoning in other settings^18^, there was substantial variability in how quickly participants managed to learn and adapt to the opponent level (Fig. 2f).

In the CHASE model, variability in adaptation is captured by a parameter $\gamma$ that governs the level of noise associated with detecting a particular strategy in observed actions (Methods). This parameter also varied substantially among participants (Supplementary Fig. 13), and simulations indicated that it is a key determinant of behavioral success in the game (Supplementary Results). Moreover, $\gamma$ directly determines the strength of the model-derived opponent-level BUs, that is, the learning signals about opponent strategy, which were also highly variable across participants (but clearly decreased over time within a game; $\beta$ = −0.038, *t*(84.4) = −20.6, *P* < 10^−34^; Fig. 2g).

Finally, we examined whether using more sophisticated strategies was associated with longer response times. We correlated the model’s trial-by-trial estimates of the level-*k* strategy used with response times on each trial. As expected, higher inferred levels were linked to slower responses (even when controlling for opponent sophistication; $\beta$ = 0.047, *t*(106.7) = 2.5, *P* = 0.014 in dataset 2a, $\beta$ = 0.032, *t*(47.8) = 2.1, *P* = 0.041 in dataset 2e). This pattern is consistent with the notion that more recursive reasoning steps require more time.

Overall, the CHASE model captured participants’ ability to flexibly adapt their mentalization level across different opponent types. Crucially, the model’s trial-by-trial estimates revealed substantial individual variability in the amount of evidence participants required before adapting to an opponent’s strategy.

### fMRI results

Encouraged by the behavioral results, we used fMRI to investigate the neural basis of the adaptive mentalization processes formalized in the CHASE model, which could not be identified by models of static mentalizing strategies. A subset of participants underwent fMRI while playing RPS games against three different types of artificial opponents (each opponent type twice in random order; dataset 2e; *n* = 50; classical RPS game with three actions; see Methods for details). Each game consisted of 40 rounds and participants were told that they would be rematched with a different online human opponent after each game.

### Brain regions linked to evaluation and adaptation of mentalization strategies

First, we examined whether the model-derived computations corresponded to brain activity, to provide neural evidence for the assumptions embedded in the CHASE model and to clarify whether adaptive mentalization is encoded in similar or distinct areas from those found to correlate with static belief inference^29^. Building on previous evidence on these brain regions, we focused on regions of interest (ROIs) selected a priori by an automated meta-analysis for the term ‘theory of mind’ using Neurosynth^30^. However, most results also hold when performing whole-brain inference (Supplementary Fig. 11).

**Fig. 3:**
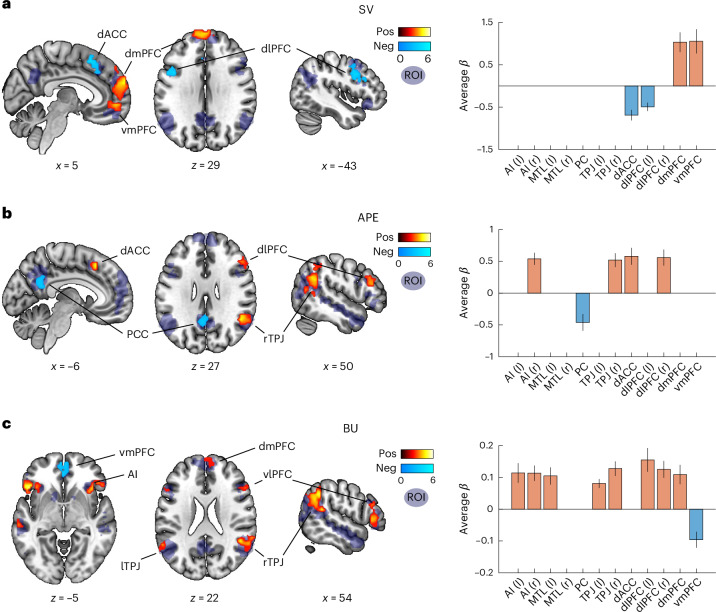
Key model variables are encoded in different patterns of brain activity during action selection and feedback. **a**, Correlations of brain activity with model-derived SV during the choice phase, capturing the reward that participants are expecting when playing their chosen action. Left, *t* values from SPMs of positive (hot colors) and negative (cold colors) correlations superimposed on sagittal and horizontal brain slices (all *P* < 0.05 FWE SVC within an a priori social brain mask (in blue); cluster-forming threshold *z* = 2.408; see Supplementary Tables 4–6 for *P* values per cluster). Positive activation was found in vmPFC and dmPFC (peak at MNI *x*, *y*, *z* = 3, 63, 27, respectively; *k* = 336; *P*_FWE_ < 0.001; Supplementary Table 4), as well as dlPFC (peak at MNI *x*, *y*, *z* = −31, 3, 58; *k* = 138, respectively; *P*_FWE_ = 0.003), and deactivation in dACC (peak at MNI *x*, *y*, *z* = −1, 12, 51, respectively; *k* = 97; *P*_FWE_ = 0.008) with increasing CV. Displayed are representative slices visualizing the location and extent of the clusters (note that they do not exactly match the peak coordinates). Right, average neural *β*s within significant clusters in the different ROIs (all *P* < 0.002 uncorrected; mean across participants ± s.e.m.; ROIs with no significant clusters are omitted; *n* = 48; see Supplementary Fig. 15 for individual data points). **b**, APE during feedback phase shows associations within a distributed network of the social brain encompassing several regions (rTPJ—peak at MNI *x*, *y*, *z* = 48, −49, 27, respectively; *k* = 167; *P*_FWE_ = 0.001; dACC—peak at MNI *x*, *y*, *z* = −7, 21, 48, respectively; *k* = 54; *P*_FWE_ = 0.038; right dlPFC—peak at MNI *x*, *y*, *z* = 45, 33, 20, respectively; *k* = 49; *P*_FWE_ = 0.047; and right AI—peak at MNI *x*, *y*, *z* = 36, 24, −5, respectively; *k* = 47; *P*_FWE_ = 0.050; as well as a deactivation in the PCC—peak at MNI *x*, *y*, *z* = −1, −49, 20, respectively; *k* = 65; *P*_FWE_ = 0.025; Supplementary Table 5). Left and right as in **a**. **c**, Model-derived BU (that is, a participant’s change in belief about the opponent’s level of sophistication; formally defined as the KL divergence between two successive beliefs) correlates with neural activity in several areas (rTPJ—peak at MNI *x*, *y*, *z* = 51, −43, 16, respectively; *k* = 165; *P*_FWE_ < 0.001; lTPJ—peak at MNI *x*, *y*, *z* = −58, −46, 37, respectively; *k* = 125; *P*_FWE_ = 0.002; right AI/vlPFC—peak at MNI *x*, *y*, *z* = 39, 27, −2, respectively; *k* = 116; *P*_FWE_ = 0.004; left AI/vlPFC—peak at MNI *x*, *y*, *z* = −46, 24, −5, respectively; *k* = 118; *P*_FWE_ = 0.003; and dmPFC—peak at MNI *x*, *y*, *z* = 6, 51, 44, respectively; *k* = 60; *P*_FWE_ = 0.021; as well as deactivation of vmPFC—peak at MNI *x*, *y*, *z* = −4, 45, −9, respectively; *k* = 60; *P*_FWE_ = 0.021; Supplementary Table 6). Left and right as in **a**. MTL, medial temporal lobe; SVC, small volume correction; SPM, statistical parametric map; PCC, posterior cingulate cortex; Pos, positive; Neg, negative.

Specifically, we computed the subjective value (SV) of the chosen action while participants were making a choice, as well as the action prediction error (APE) and the opponent-level BU when participants learned about the opponent’s action. Please note that all these signals were derived from dynamically evolving beliefs about the opponent’s strategy embedded in the CHASE model, rather than from the application of a single strategy as in previous investigations. As an initial validation, we examined value-related model variables during the choice period, which should correlate with activity in brain regions known to encode SV, such as ventromedial prefrontal cortex (vmPFC)^31^. Conforming to our prediction, and in line with previous findings^10^, we found significant activation in vmPFC, dorsomedial PFC (dmPFC), as well as dorsolateral PFC (dlPFC), and deactivation in dorsal anterior cingulate cortex (dACC) with increasing choice value (CV; Fig. 3a and Supplementary Table 4).

Next, the CHASE model provided dynamic, trial-wise estimates of participants’ beliefs about the opponent’s most likely next action. We used these beliefs to examine an APE signal in the brain during the feedback phase (Methods), which here depends on dynamic adaptation of opponent-level inference rather than on a fixed strategy as in previous studies^10^. We found activity related to this dynamic APE in the right TPJ (rTPJ), dACC, right dlPFC and right anterior insula (AI), as well as a deactivation in the posterior cingulate cortex (Fig. 3b and Supplementary Fig. 15).

We then examined the most crucial computation embedded in the CHASE model—the extent to which participants update their beliefs about the opponent’s level (defined for each trial as KL divergence between the previous and present distributions of beliefs about the opponent’s strategy *k*; Figs. 1d and 2g; Methods). Trial-by-trial changes in these beliefs reflect how strongly participants revise their model of the opponent’s reasoning process, which is essential for correct mental state inference and selecting the appropriate strategy in response. When regressing neural activation on this BU signal, we found significant bilateral activation in the TPJ, AI, ventrolateral PFC (vlPFC) and dmPFC, as well as deactivation in vmPFC (Fig. 3c). To rule out the possibility that this analysis was confounded by correlations between BUs and APEs, or by generic temporal effects (for example, adaptation/novelty or other quantities that decrease monotonically throughout a run), we performed several control analyses. These confirmed that our findings were robust to statistical controls for both correlations between BUs and APEs, as well as any potential surprise or temporal adaptation effects (Supplementary Results).

In summary, our results indicate that the TPJ is involved in the flexible evaluation (APE) and adaptation (BU) of mentalization strategies, not merely the execution of a single such strategy, thereby extending earlier accounts of its role in social cognition^13,14^. These computations are not restricted to one specific level of sophistication, but are a general feature of updating beliefs from *any* level to another, indicating a more general role of the TPJ than previously assumed^11,12^.

### Individual sensitivity to opponent strategies relates to distributed neural processes

To examine possible neural origins for individual differences in adaptive mentalization, we examined how brain activity related to the model parameter $\gamma$, which governs the strength of the trial-wise BU signal. Conceptually, $\gamma$ has an effect similar to a Bayesian learning rate—individuals with high (low) $\gamma$ strongly (weakly) update their beliefs in response to an opponent’s behavior. We examined two possibilities of how brain activity may relate to this parameter: $\gamma$ may correlate with localized changes in activation of single brain regions (for example, TPJ, dmPFC), or may instead relate to differences in functional connectivity within the social brain network.

To examine this, we first included $\gamma$ as a participant-wise covariate in a second-level model of the mean activation during the feedback phase, extracted from first-level models that did not contain BU (to avoid statistical circularity). No single region showed a significant correlation.

**Extended Data Fig. 1:**
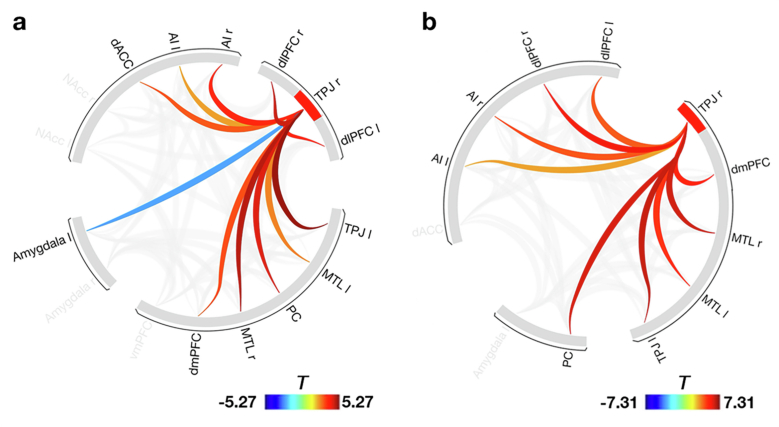
Seed-based connectivity (SBC) analysis using the right TPJ as the seed region. **a**, Participants with a higher estimated $\gamma$ parameter, capturing one’s sensitivity to information about the strategy of the other, exhibited stronger rTPJ connectivity across most regions within the social brain network (11 of 15 ROIs), with particularly strong correlations observed with the dlPFC, AI, and dmPFC (colors indicate *T*-values). After denoising (Methods), functional connectivity was computed based on time series epochs corresponding to the feedback phase. Standard preprocessing and denoising procedures were applied, and connectivity was assessed using the rTPJ as the seed region with individual $\gamma$ values as a covariate on the second level. Results were thresholded using family-wise error (*p*_FWE_ < 0.05) correction for multiple comparisons. **b**, The same analysis in the replication sample. Nine of 11 initially significant ROIs were successfully replicated (*p*_uncorrected_ < .05).

We then tested the second possibility—whether $\gamma$ was reflected in dynamic functional connectivity across the brain network implicated in the task. As previous work^10,11^ had identified rTPJ connectivity patterns during static mentalizing, we examined how connectivity of this area covaried across participants with $\gamma$. We conducted a seed-based connectivity analysis with rTPJ as the seed region, testing whether the strength of its connectivity with the other 15 social brain ROIs during the outcome phase covaried with individual $\gamma$. As shown in Extended Data Fig. 1a, higher $\gamma$ values were associated with stronger rTPJ connectivity across 11 of 15 ROIs (*P*_FWE_ < 0.05), with particularly strong associations observed in the AI (r), dlPFC (r) and TPJ (l). These findings suggest that participants who are behaviorally more sensitive to information about opponents’ strategy exhibit greater functional integration of the rTPJ within the social brain network.

### Opponent-level BUs can be decoded from neural activity

We then addressed the question of whether adaptive mentalization can be decoded from neural activity alone. Given sufficient specificity and sensitivity, such a neural marker could help assess the extent of adaptive mentalization in an unobtrusive way and may have clinical utility (for example, in studies with autism spectrum disorder)^24^. Accordingly, we used a multivariate machine‑learning approach^32^ to test whether participants’ level *k* (reasoning steps) and the magnitude of their belief updating could be decoded from whole‑brain activity, using leave‑one‑participant‑out cross‑validation (Methods)^33^.

First, we tested whether we could decode the model-derived level of strategic sophistication (the number of recursive reasoning steps, *k*) that participants used predominantly during each run. Providing additional neural evidence for the CHASE model, we could indeed decode the level *k* of participants’ current strategy from whole-brain neural activation during both choice and feedback, with higher fidelity during feedback (choice—accuracy = 39.19%, *P* = 0.044; feedback—accuracy = 43.33%, *P* = 0.001; chance level = 33%).

**Fig. 4:**
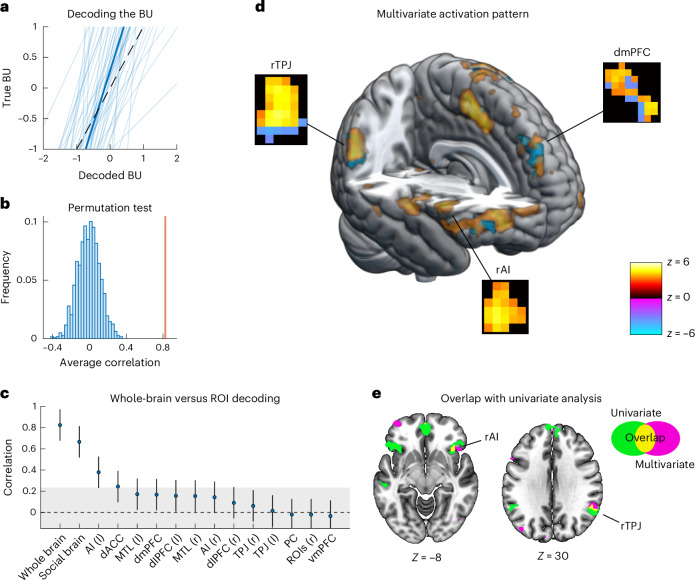
Adaptive mentalization —dynamic update of opponent-level beliefs —can be decoded with high out-of-sample accuracy from neural activity. **a**, Correlations between actual and (out-of-subject) predicted updates of the belief about opponent level are positive across all participants and very high on average (*r* = 0.82, 88% participants *r* ≥ 0.5, *P* < 0.0002 two-sided based on permutation testing). Individual correlation coefficients were first Fisher’s *z* transformed to avoid distortions in the means due to the skewed sampling distribution. Plotted are regression lines from individual participants (thin blue lines) and the pooled sample (thick blue line). The dotted black line represents ideal decoding. **b**, A permutation test reveals that this average correlation (orange line) is far apart from the correlations expected by chance (chance distribution plotted as blue histogram). **c**, MVPA decoding using only voxels from different (combinations of) ROIs reveals that areas outside the social brain also contribute to the predictive performance, as the decoding performance drops noticeably when constraining the training data to voxels within the social brain (to *r* = 0.65). When just training on the voxels in individual ROIs, the left AI and the dACC also allow for above-chance decoding (*r* = 0.38 and 0.24, respectively). Plotted are mean (and s.e.m.) correlations for each ROI; gray area indicates chance level based on the permutation distribution from **b** (*n* = 48). **d**, Thresholded *z*-value map (*P* < 0.001, uncorrected) of the multivariate activation pattern predicting BUs. The multivariate activation patterns were derived based on the dot product of the neural signature weights and the BOLD signal. Highlighted rectangles visualize multivariate patterns within selected clusters of interest (rTPJ, dmPFC and rAI). Warm (hot) colors represent positive relations, while cold (cool) colors indicate negative relations of voxel activity with predicted BUs. All weights are *z* transformed to highlight regions that contribute most consistently with either positive or negative values across all participants. Some regions—such as the bilateral AI, bilateral dorsal striatum and bilateral vlPFC—exhibit positive contributions to BU, whereas the dmPFC and rTPJ display specific spatial patterns of both positive and negative contributions (see ‘Analyses of the neural signature pattern’ in Supplementary Methods as well as Supplementary Tables 13 and 14). Please note that unthresholded patterns are used for prediction; the thresholded (*P* < 0.001, uncorrected) patterns shown here are for illustration purposes. **e**, The multivariate weight map overlaps with the univariate analysis in the rTPJ and bilateral insula (plotted are voxels with significant weights from the multivariate analysis with *P*_FDR_ < 0.05 based on 5,000 bootstrap samples, and univariate *β*s with *P*_FWE_ < 0.05 SVC).

We then asked whether the model‑derived BUs—the process of adapting to another’s strategy—could be decoded out of sample from whole‑brain activity. Although the BU is purely model-derived, predicting it from neural activity alone was possible with very high accuracy (average per-participant correlation between actual and predicted label, *r* = 0.82; overall correlation in pooled data, *r* = 0.49, *P* < 0.0002 based on permutation testing; Fig. 4a,b and Supplementary Fig. 16 for bin-level predictions), serving as further neural validation of the CHASE model. This high predictive accuracy was very consistent across participants (in 88% participants, *r* ≥ 0.5). Several robustness checks confirmed that BU decoding was not confounded by surprise (APE), reward, reaction times or by generic temporal confounds (linear and quadratic time trends; Supplementary Results and Supplementary Fig. 18). Notably, in contrast to the multivariate patterns linked to particular levels (see above), BU is independent of any particular level. Because the BU captures the general process of adapting one’s strategy to the other’s way of reasoning, rather than simply inferring beliefs with a static strategy, we refer to the resulting neural activation pattern as a ‘general neural signature of adaptive mentalization’.

To test whether the identified neural signature was specific to the social brain network or distributed throughout the whole brain, we repeated the decoding procedure using only voxels within social brain ROIs, either together or in each region individually. This allowed us to assess which regions are necessary for predicting adaptive mentalization out of sample. Limiting the analysis to social brain voxels substantially reduced predictive accuracy (average correlation *r* = 0.65; Fig. 4c and Supplementary Table 13 for a parcellation of all significant weights from the whole-brain model), indicating that areas outside the social brain contribute to performance. At the level of individual ROIs, patterns derived from the left AI and the dACC performed significantly above chance (*r* = 0.38 and 0.24, respectively), whereas patterns from other regions failed to decode BUs in isolation (Fig. 4c). Thus, multivariate patterns restricted to single regions lack sufficient specificity and accurate inference about mentalization requires distributed information across the brain.

The whole‑brain decoding results raise a key question—does prediction rely on fine‑grained, distributed voxel patterns or merely on mean ROI activation? To test this, we repeated the procedure from above using the mean *β* per ROI, either individually or combined, instead of multivoxel patterns. While some of these models also provided above-chance performance, they performed substantially worse than our whole-brain multivariate pattern (Supplementary Fig. 17). Specifically, voxel‑level decoding exhibited almost a doubling of variance explained relative to mean‑ROI decoding (67.9% versus 34.8%). This suggests that variability in fine-grained voxel-level patterns within and across ROIs contains important information about the BU signal.

To shed light on these specific spatial patterns, we examined which voxels consistently contributed to predicting the BU signal across participants. We first calculated the dot product between the blood-oxygen-level-dependent (BOLD) responses to BU and the neural signature weights derived from the adaptive mentalization decoder. This approach yielded voxel-wise activation values for BU, which, when summed up, result in the decoder’s prediction of BU. We repeated this procedure for all participants and subsequently converted the activation values to *z* scores to obtain the voxels that most consistently contributed to BU across participants. Figure 4d displays the thresholded *z* mask (*P* < 0.001). The distribution of activations varied considerably across the brain and within specific ROIs. Specific regions—such as the bilateral AI, bilateral dorsal striatum and bilateral vlPFC—exhibited mostly positive contributions to BU, whereas other regions, including the dmPFC and rTPJ, displayed both positive and negative contributions (Supplementary Fig. 9). These findings revealed a nuanced functional organization, such as a ventral-to-dorsal spatial gradient in rTPJ and a center-surround pattern in dmPFC, implying a spatially specific neural code of the key adaptive mentalization computation captured by the CHASE model.

Finally, we examined whether the univariate analysis described above and the multivariate approach yield convergent findings. Indeed, we found an overlap of significant voxels from both approaches in the rTPJ and the bilateral AI, corroborating their role in interpersonal belief updating (based on 5,000 bootstrap samples with *P*_FDR_ < 0.05; Fig. 4e). However, a substantial number of significant multivariate voxels were outside the significant clusters from the univariate analysis (Supplementary Table 13). This shows that univariate and multivariate analyses partially converge, but also underscores the necessity of multivariate analyses when predictive power is required.

### Replication in an independent dataset

Given the high consistency of our neural results across participants, we assessed the out-of-sample robustness and ecological validity of the neural signature of adaptive mentalization by replicating our findings in a more demographically diverse group of participants (*n* = 47, 57% female, age = 32 ± 8.2 years, years of education = 16 ± 3.1; Methods).

**Fig. 5:**
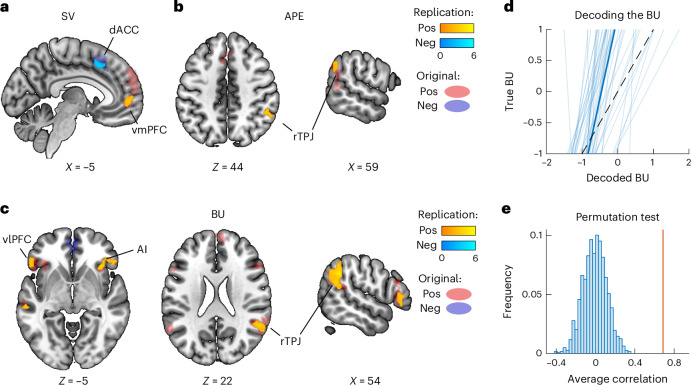
Replication of the neural results in an independent, demographically more diverse sample. **a**, Significant activation in line with the model-predicted SV of the chosen action during choice. Shown is the significant activation in the replication sample (solid; yellow = positive, light blue = negative) within the significant clusters from the primary dataset (transparent; red = positive, dark blue = negative; Fig. 3). **b**,**c**, Same as in **a**, but for APEs and the BU, respectively. **d**, When applying the neural signature (that was trained on the primary dataset) to this completely unseen data, the decoding accuracy drops only slightly and remains at a very high level (*r* = 0.67, 74% participants *r* ≥ 0.5). **e**, A permutation test reveals that this average correlation (orange line) is still far away from the chance distribution (blue histogram, *P* < 0.0002 two-sided).

First, almost all univariate findings were replicated (that is, all clusters with *P* < 0.01 in the discovery sample, except dlPFC for SV and left TPJ for BU; Fig. 5a–c, Supplementary Tables 10–13 and Supplementary Fig. 15). In line with this consistency, *t* values exhibited a high spatial correlation across voxels between the two samples, indicating that the neural activation pattern was also similar below the threshold for statistical significance (BU, *r* = 0.72; APE, *r* = 0.55; SV, *r* = 0.42; all *P* < 0.0001).

We then examined the replication of the functional connectivity analyses related to the parameter $\gamma$, and successfully replicated 9 of the 11 initially significant ROI correlations (*P*_uncorrected_ < 0.05; not replicated—left amygdala and the dACC). These results confirm that functional network integration of the rTPJ within the social brain network is stronger in individuals with higher sensitivity to their opponent’s strategy (Extended Data Fig. 1b).

Finally, we tested whether the identified multivariate neural pattern—the neural signature of mentalization—could predict the BU in this independent dataset. Despite being demographically more diverse, the prediction performance only dropped slightly (BU, *r* = 0.67; *P* < 0.0002 based on permutation testing) and was robust to several control analyses (Supplementary Results). Thus, the multivariate neural signature of adaptive mentalization we identified generalizes across different groups and demographics, suggesting that it may hold potential for clinical applications.

## Discussion

The brain processes enabling humans to mentalize have been intensely studied in neuroscience, but most approaches have focused on static strategies^1,8,29,34,35^ that cannot adapt to the idiosyncratic, changing thoughts and actions of interaction partners^20^. Here we propose and empirically validate a new account of adaptive mentalization that can flexibly track dynamic adjustments of inferences about others’ mental states.

We show that individuals are capable of flexibly adapting to others’ strategies, and that our model of adaptive mentalization (CHASE) outperforms existing alternative models in a large sample (*n* = 553) across various game settings. fMRI measures reveal a dynamic APE signal—based on changing beliefs about the opponent strategy—in the rTPJ and a mentalization-related BU in the rTPJ and AI (extending into vlPFC; with additional clusters in left vlPFC and medial temporal lobe)^10,11,13^. Crucially, a key model parameter ($\gamma$, governing sensitivity to opponent-level information) is associated with functional connectivity of the rTPJ with the social brain network. We further construct a multivariate neural signature that allows out-of-sample prediction of the extent of this BU signal based on task-related neural activations. The high predictive performance of this decoder (main sample, *r* = 0.82; replication sample, *r* = 0.67) suggests that it may be useful as a neural marker for diagnostic assessments and could inform the design of interventions in demographically diverse samples^24,32^.

Many existing theories have characterized mentalization either as a static process^7,9,36^ or have constrained their approach to one-shot interactions, possibly due to the complexity of modeling dynamic, adaptive mentalization in repeated interactions^6,18^. Here, building on previous theoretical frameworks^5,20^, we provide modeling and neural evidence that participants can dynamically adjust their inference process to match their opponent’s idiosyncratic way of strategizing across repeated interactions^23,37^. To capture this ability, we introduce a belief-updating model combined with a simple game paradigm (Fig. 1b) and calibrated artificial opponents, providing a reliable tool for assessing adaptive mentalization in evolving interactions.

Consistent with prior work, some participants reached a ceiling against highly sophisticated opponents^18,38^, suggesting individual limits in reasoning depth. However, most achieved level 3, indicating greater sophistication than static, one-shot measures typically revealed. Model comparison showed that players inferred opponents’ behavior primarily from observed action frequencies rather than from rewards or counterfactuals^19,23^, although human–human play also involved some reward learning. Moreover, participants based their responses on a distribution of beliefs about opponent sophistication, more consistent with cognitive hierarchy than single-level reasoning^6,18,23^. These findings have direct implications for computational modeling of social reasoning—they constrain which informational cues people are likely to track, clarify the attainable depths of recursive reasoning and highlight that individuals may maintain graded belief distributions. Together, these results narrow the modeling space for future studies of repeated interactions in mixed-strategy games and emphasize the value of explicitly modeling belief updating. CHASE thus provides a simple yet extensible framework for capturing dynamic mentalization; future work may extend it to evolving opponent models, nonuniform priors or richer forms of social inference and multistep planning across broader game structures^19,23,37,39^.

From a neuroscience perspective, our findings extend current accounts of the role of social brain areas—particularly the rTPJ—in social cognition^14,40^. Previous work has linked the TPJ to static belief attribution in strategic settings^7,10,11^, but it has remained unclear whether this region also supports higher-level adaptive inference. We show that the rTPJ contributes to dynamically adjusting one’s mentalization depth to an opponent’s strategy, with this adaptive function localized to anatomically similar TPJ subregions as implicated in static mentalization^7,9^. Unlike earlier studies associating regional activity with fixed levels of recursive reasoning (for example, inferring what you are thinking, or what you are thinking I am thinking)^10,11^, we find that rTPJ activity reflects a more general update in beliefs about ‘how’ others reason. Thus, activation across the social brain seems to track general beliefs about an opponent’s reasoning process, rather than single-level strategic inferences. The previously reported correlations^11,13^ and causal links^11^ between rTPJ activity and second-order beliefs may therefore index the adaptive transition from simpler to more complex levels of reasoning, which static models capture as increased engagement in higher-level strategies.

The neurocomputational role we propose here for the social brain network also differs from previous accounts of observational learning, which have proposed brain mechanisms of arbitration across different strategies^41,42^ that may also constitute a form of dynamic adaptation in social interactions. For example, previous research has found that activity in both vlPFC and TPJ may be linked to arbitration between choice imitation and goal emulation^41^ and between cooperative and competitive intentions^42^. However, while these accounts also describe types of dynamic adaptation, they do not entail any recursive mentalization but rather different types of learning from others’ behavior, without considering that others might be responding to one’s own behavior in return.

In addition to information about functional contributions of brain regions, we show that a whole-brain multivoxel pattern identified through machine learning encodes BU signals beyond what is captured by mean activity, local ROI patterns or aggregated social brain signals. This indicates that adaptive mentalization relies on a distributed network engaging multiple cognitive systems, potentially interfacing with systems involved in working memory, valuation and recursive reasoning. Our results also refine the characterization of mentalization-related activity within the social brain: while some regions (for example, AI, vlPFC) show uniformly directed contributions to BUs, areas such as rTPJ and dmPFC exhibit mixed spatial patterns of positive and negative weights, revealing fine-grained multivariate organization and functional gradients not accessible to univariate approaches. These findings highlight the multifaceted and widely distributed neural basis of adaptive mentalization. At the same time, our results also confirm that this adaptive mentalization network is remarkably homogeneous across participants, indicating that information that is encoded at a higher granularity than meta-analytically derived ROIs is preserved across participants, in line with previous work using similar methodology^24^. While this pattern does not necessarily rely on single voxels, it can uncover task-specific multivoxel subdivisions of well-known ROIs. This generalizability raises the questions whether it is an innate structure specialized for adaptive mentalization, and whether there is a genetic basis that could account for individual, potentially clinically relevant, differences. The approach we introduce here may help to address those questions in future studies, as it provides a very fine-grained assessment (on a trial-by-trial level) of the cognitive processes and the associated neural activity underlying adaptive mentalization^5,19,21^.

In addition to the experimental paradigm, modeling approach and insights into the neural processes underlying adaptive mentalization, our study also establishes a multivariate neural signature that can be used to assess this ability purely from neural activation patterns rather than behavioral observations^24^. Such a neural marker may be able to differentiate between individuals who merely mimic mentalization as a coping strategy and those who genuinely engage in cognitive mentalization processes. For example, the diagnosis of autism spectrum disorder currently relies heavily on subjective assessments through lengthy interviews and multiple questionnaires^43^, leaving room for subjectivity and interpretation. The neural signature for mentalization identified here holds potential to have the required sensitivity and specificity to reliably predict the adaptiveness of mentalization—specifically, the correlation between neurally predicted and model-inferred adaptive mentalization was *r* ~ 0.80, which compares favorably with previously reported decoding accuracies from neural data (0.74 for pain ratings^44^, 0.53 for craving^24^ and 0.92 for rewards^24,32^).

However, there are several limitations that need to be considered for such a possible application. First, we applied our model only to one strategic setting (the RPS game) rather than to a general set of games or social situations that involve mentalization. Second, although the ability to form higher-order beliefs (such as ‘I think that you think that I think’, etc.) is a fundamental aspect of mentalization, it is crucial to acknowledge that mentalization may encompass a broader range of dimensions, such as emotional empathy^35^. Third, despite the relatively high predictive performance of our neural marker, future studies should test the extent to which the neural marker established here generalizes to other experimental settings assessing social interactions.

In sum, we provide a comprehensive empirical characterization of the cognitive and neural processes underlying adaptive mentalization. Our approach, and the identified neural signature in particular, holds potential for assessing an individual’s ability to adapt their social inference to the idiosyncratic reasoning of interaction partners. This critically advances our understanding of this remarkable human ability, offers a new way of investigating it further and may help to improve diagnosis and therapy of corresponding difficulties in brain disorders.

## Methods

### Participants

The study was approved by and conducted in accordance with the policies of the Institutional Review Board of the University of Zurich and the Cantonal Ethics Commission in Zurich (study protocol 2019-00653). We recruited 506 healthy participants from the participant pool of the Department of Economics at the University of Zurich. Fifty of these completed the task while undergoing fMRI (all males, age = 23.44 ± 2.57 years), the others only performed a behavioral version of the task (56% females, age = 24.7 ± 4.7 years). To replicate our neural results and to test the generalizability of the neural signature in a more diverse sample, we also report analyses from another sample of participants that constitutes the control group of a clinical study that will be reported in a separate publication (*n* = 47). Notably, these participants markedly deviate from the default young student population demographic in neuroimaging research (*n* = 47, 57% female, age = 32 ± 8.2 years, years of education = 16 ± 3.1). For the fMRI sessions, we excluded participants ineligible for fMRI scanning according to standard MRI safety exclusion criteria. All participants gave written informed consent. To ensure sufficient power (of at least 80%, to detect moderate effect sizes with *P* < 0.05) and to validate and characterize the CHASE model, we chose a large sample size for the behavioral experiments, an order of magnitude larger than typical behavioral experiments. For the fMRI experiment, our chosen sample size provides sufficient power (of at least 80%) to detect moderate effect sizes with *P* < 0.05, which is comparable to or exceeds the average sample size of most fMRI studies^5,10^.

### Experimental design

#### Task

Participants played variants of repeated RPS games against human and artificial opponents. In every round, both players simultaneously picked one of three available actions (‘rock’, ‘paper’ or ‘scissors’). Each action beat one action and, in turn, got beaten by the third, leading to a circular, nontransitive dominance structure (that is, ‘rock’ beats ‘scissors’, ‘scissors’ beats ‘paper’, ‘paper’ beats ‘rock’). To reduce cognitive demands and reinforce this payoff structure, as well as to allow for a straightforward extension of the action space (that is, introducing a fourth action), we presented the game in the form of a circle where participants picked a number instead of symbols (from 1 to 3 or 4 depending on game variant; Fig. 1a). Then, the player who picked the number that was exactly one step ahead of the opponent’s number won the round (and the other player lost; choosing the same action resulted in a tie; the direction of the action dominance was indicated by arrows).

To test the robustness of our findings, we performed several experiments where we modified one or several components of the game, namely (1) the number of possible actions (3 versus 4), (2) the memory demands (by displaying the recent history in the game, the last 12 trials), (3) the used incentive scheme (zero-sum or not) and (4) the opponent (human or artificial). Across all experiments, participants were instructed that they would be randomly matched with other participants to play several matches lasting 30–40 rounds each. Participants typically played six matches (and were always instructed to meet a new opponent in each match), leading to a total of 180–240 trials (see Supplementary Table 1 with the exact specifications of the different experiments).

In the ‘discovery fMRI’ dataset, participants played against three different artificial opponent types in the scanner (with either 0, 1 or 2 steps of reasoning). Throughout each run, participants played against the same opponent type, and after each run they switched to another opponent type to avoid subsequent repetitions. Specifically, they played against each opponent twice in a counterbalanced order, leading to six runs in total. One run consisted of 40 trials, while each trial consisted of a fixation phase (0–6 s), a response phase (3–6 s) and feedback phase (2 s). Intertrial intervals (ITIs) were carefully chosen through simulations to maximize design efficiency and decorrelate action from feedback phase. On average, trials lasted around 7.3 s, with a total of 24 min of the game inside the fMRI scanner.

For the ‘replication fMRI dataset’, we collected more data by increasing the number of observations per participant (instead of scanning more participants), and thus changed the experimental timing in the replication fMRI dataset as follows. Again, participants played against three different opponent types in the scanner (with either 0, 1 or 2 steps of reasoning). However, this time, participants played thrice against each opponent, again with counterbalanced opponent type order to avoid subsequent repetitions, leading to nine runs in total. One run consisted of 40 trials, while each trial consisted of a fixation phase (1–3 s), a response phase (3–5 s) and feedback phase (2 s). Again, ITIs were carefully chosen through simulation to maximize design efficiency and to decorrelate response from the feedback phase. On average, trials lasted around 7.3 s, with a total of about 45 min of the game inside the scanner.

All games were incentivized—the final game score was converted to CHF based on predetermined conversion rates (3-to-1 for fMRI and 4-to-1 for the behavioral experiments) and paid out at the end of the experiment (in addition to a fixed show-up rate). On average, participants earned 69.4 ± 6.4 CHF in the fMRI experiment and 30.5 ± 5 CHF in the behavioral experiments.

#### Artificial opponents

To provide a general-purpose measure of mentalization, we aimed to (1) make sure all participants interact with behavior that is equally informative about the opponent’s strategy, and (2) test how flexibly (and quickly) participants can adapt to a range of different sophistication levels. To achieve this level of experimental control, we used artificial opponents that mimic human behavior. This approach also allowed to induce high levels of reasoning (*k* = 3) that earlier models of repeated strategic interactions were typically not able to capture.

To ensure that the artificial opponents are as human-like as possible, we based them on the CHASE model that we also use to explain participant behavior, as this model provided the best account of participant behavior across a wide range of game specifications in human-versus-human gameplay (see Fig. 2b for model comparison and below for a specification). In particular, the artificial opponents used the same learning rule and recursive reasoning mechanism, but were fixed to one level of sophistication (either *k* = 0, 1 or 2; that is, no adaptive BUs). In addition, we carefully calibrated the noise structure (when and how they deviated from their strategy) to balance informativeness and human-likeness over a series of behavioral experiments (datasets 2c and 2d; *n* = 134; see Supplementary Methods for details).

To confirm that the resulting artificial opponents cannot be distinguished from human opponents, we conducted a Turing-test-like assessment in three behavioral sessions. As in all behavioral experiments, participants were instructed to play against other participants who were simultaneously tested (18 participants per session) in the same room (in small cubicles for anonymity). At the end of the experiment, we asked them to rate on a five-point Likert scale whether the different opponents they played with were human or artificial. We conducted a Komogorov–Smirnov test to assess whether there are any differences between the ratings for human and artificial opponents (Fig. 2a).

The behavioral experiments took place in one of the large behavioral labs at the UZH Department of Economics, accommodating up to 35 participants per session. All experiments were fully computerized and participants were seated in individual cubicles. The computerized environment and the arrangement of cubicles ensured that participants remained anonymous, preventing them from discerning the identity of their opponents (human or artificial opponent).

In both of our fMRI experiments, participants were informed that they were competing against opponents situated in a neighboring room. They were informed that we implemented meticulous measures to ensure that participants remained unseen by one another throughout the experiment, thereby eliminating potential bias in their beliefs or behaviors. Specifically, participants were told that their opponents were located in an adjacent behavioral lab within the facility, which they passed by and witnessed when entering the scanner room (this behavioral lab room is equipped with 14 computerized cubicles for behavioral experiments). To enhance the realism of this setup, we conducted connection checks on the screen before commencing the experiment. Furthermore, at the beginning of each run, we simulated delays, suggesting that we were waiting for the readiness of other participants. This was convincingly done either through simulated telephone calls or by research assistants, indicating that we were awaiting the signal to start, thereby reinforcing the impression of a large experimental environment. Coupled with the carefully calibrated bot, which passed a modified Turing test to ensure human-like behavior, these elements collectively fostered a convincing experience of engaging in real-time competition with other human opponents.

### Computational models

To link the observed behavior in the game (a series of actions) to putative underlying cognitive mechanisms and strategies, we considered and compared a series of cognitive-computational models that people might be using. These included simple learning rules (for example, reinforcement learning or fictitious play) and more complex strategies that alternate among different learning rules or propose additional mechanisms of responding to the opponent (such as experience-weighted attraction, EWA).

### CHASE model

In line with earlier models of recursive reasoning^18–20^ and related empirical findings^25^ that humans systematically deviate from random gameplay, our proposed CHASE model assumes that players base their mentalizing on the assumption that there is a salient action that nonstrategic players (defined as *k* = 0) would choose more often than others. Strategic players then add a limited number (*k*) of recursive reasoning steps by iteratively best-responding to that action (for example, if ‘rock’ is salient, a *k* = 1 agent will choose ‘paper’, a *k* = 2 agent ‘scissors’, etc.). In addition, adaptive agents infer the level of recursive reasoning of the opponent by integrating evidence for the different levels over time. More formally, the proposed modeling approach is based on three main assumptions.

#### A1: nonstrategic play (*k* = 0) is governed by a Markovian updating rule

This updating rule maps the history of the game (past actions and rewards) to attractions $A\left(a\right)$ for each action $a$ at each trial $t$, quantifying how salient or ‘attractive’ the different actions appear to be for a nonstrategic agent. In the context of the RPS under study here, we empirically identified this learning rule to be a simple delta rule over actions:

1 $$A{\left(a\right)}_{t+1}=A{\left(a\right)}_{t}+\alpha \times \left({\bf{I}}\left(a\right)-A{\left(a\right)}_{t}\right)$$

where **I**(*a*) is an indicator vector that is 1 for the chosen action and 0 for all other actions, and $\alpha$ acts as an inverse forgetting rate that determines how quickly the agent forgets about past actions (or, equivalently, how much she is influenced by the most recent actions; Supplementary Methods). In other words, attractions here can be interpreted as a noisy representation of historical action frequencies with an exponential memory decay. These attractions are linked to the observed behavior through a softmax function $\sigma$ with a noise parameter *β*, governing the extent to which agents tend to stick to the historical action frequencies or act randomly:

2 $$P\left({a|k}=0\right)=\sigma \left(A\left(a\right)|\beta \right)=\frac{\exp \left(\beta \times A\left(a\right)\right)}{{\sum }_{a}\exp \left(\beta \times A\left(a\right)\right)}$$

#### A2: strategic players (*k* > 0) use recursive reasoning

While a level-0 agent essentially ignores the interpersonal nature of the game, higher-level agents try to outsmart each other by predicting what the opponent will play, or predicting what the opponent thinks they will play, etc. Specifically, an agent with sophistication *k* assumes that the sophistication of the other agent is exactly one level lower (that is, *k* *−* 1) and therefore applies *k* steps of recursive reasoning. The resulting action probabilities are given by:

3 $$P\left({a|k} > 0\right)=\underbrace{\sigma \left(\right.\varPi \times \ldots \sigma \left(\right.\varPi}_{k\,{{times}}} {{\times} p\left({a|k}=0\right)\left.\right)\ldots \left. \right)}$$

where $\varPi$ denotes the payoff matrix of the game (specifying how combinations of actions map to payoffs for both players) and *σ* denotes a softmax function (as above; dropping the dependence on *β* for clarity). This means that strategic players start with the behavior of a nonstrategic agent and simulate how to respond to it, how to respond to that response, etc., up to their level *k* (notably, the level determines whose nonstrategic play acts as the starting point—odd levels are other-referential while even levels are self-referential). A softmax function is used here instead of argmax to incorporate stochasticity, capturing noisiness in action selection and recursive reasoning.

#### A3: adaptive players (${\boldsymbol{\kappa }}$ > 1) try to infer the level of the opponent

While a *k* = 1 agent is certain that the opponent must be *k* = 0 (incapable of conceiving levels equal to or higher than his own), higher-level agents face uncertainty about which level (lower than their own) the opponent is most likely playing. To adapt to different strategic players, adaptive agents thus form and update beliefs about the opponent’s level of sophistication (please note that we denote their own maximum level by $\kappa$ to distinguish it from the current level *k* of a strategic agent). In particular, they use equations (2) and (3) to compute action probabilities for each possible level lower than their own, which allows them to construct a likelihood function over levels $L({k|a})$ upon observing an opponent action $a$^Opp^:

4 $$L\left({k|a},\kappa \right)=\underbrace{\left[P\left(a={a}^{\mathrm{Opp}}|\,k=0\right),P\left(a={a}^{\mathrm{Opp}}{|k}=1\right),\ldots \right]}_{{\kappa}-1\,{\mathrm{elements}}}$$

Then, they use this likelihood to update their beliefs $B(k)$ about the level of the opponent, based on their priors from the previous trial, using Bayes rule:

5 $$B{\left({k|a}\right)}_{t+1}=\frac{{L({k|a})}_{t}\times B{\left(k\right)}_{t}}{{\sum }_{k}{L({k|a})}_{t}\times B{\left(k\right)}_{t}}$$

Finally, adaptive agents form an integrated prediction over the most likely opponent action, weighted by the belief distribution over the opponent’s level $B(k)$, and noisily best respond to it:

6 $$P\left({a|}\kappa \right)=\sigma \left(\varPi \times P\left({a|k}\right)\times B\left(k\right)\right)$$

In other words, for each possible opponent level $k < \kappa$, they consider what the opponent would play and weight this prediction by their belief that this is the opponent’s true level. This approach enables a dynamic assessment of a player’s time-varying strategy at any given point in time. Notably, it also allows for a quantification of the extent of opponent-level BU, given by the KL divergence between successive belief distributions (‘Parametric modulators’).

#### Loss sensitivity and learning differences

In addition to these main assumptions, we add two more deviations from rationality in the recursive reasoning process. First, to allow for efficient exploration of strategies, we allow for an unequal weighting of wins and losses by introducing a parameter $\lambda$ that scales the influence of losses in the payoff matrix (that is, the −1 entries; please note that the effect of this parameter is distinct from loss aversion in standard risk-taking tasks; Supplementary Results):

7 $${\varPi }_{{ij}}=\left\{\begin{array}{l}-\lambda ,\,\,\,\,\mathrm{if}\; {\varPi }_{{ij}}=-1\\ {\varPi }_{\mathrm{ij}},\,\,\,\,\,\mathrm{otherwise}\end{array}\right.$$

Second, we allow for individual differences in the ability to learn about the opponent’s level by distorting the likelihood function according to a softmax function, where the magnitude of distortion is determined by an inverse temperature parameter $\gamma$ that captures the participant’s sensitivity to evidence about the level of the opponent:

8 $$\hat{L}\left({k|a}\right)=\sigma \left(L\left(k{|}a\right)|\gamma \right)$$

This distorted likelihood function is hence used in the BU in equation (5) in place of the undistorted likelihood $L({k|a})$.

#### Free parameters

In total, the resulting model is thus characterized by the following five parameters: $\alpha ,\beta ,\gamma ,\lambda$ and $\kappa$ (regulating the speed of updating attractions, recursive reasoning noise, sensitivity to evidence for opponent’s level, loss sensitivity and depth of mentalization ability, respectively). We verified that all parameters are identifiable by performing parameter-recovery simulations (*r* from 0.73 to 1 between generating and recovered parameters; see Supplementary Methods and Supplementary Fig. 3 for details).

### Alternative models

#### Reinforcement learning (RL)

A simple nonstrategic learning rule is to repeat the actions that were successful in the past. Such an RL rule can be modeled by a delta rule of the form:

9 $$A{\left(a\right)}_{t+1}=A{\left(a\right)}_{t}+\alpha \times \left(\pi \times {\bf{I}}\left(a\right)-A{\left(a\right)}_{t}\right)$$

where $\pi$ denotes the payoffs that the agent would have received for all possible actions, given the opponent action (but please note that only the payoff of the chosen action is taken into account in the update, due to the indicator vector)^7^. Here the attractions $A(a)$ act as estimated action values (sometimes denoted *Q* values in other models). As for a level-0 agent, the behavior of an RL agent is then probabilistically governed by these attractions according to a softmax function, given some inverse behavioral decision temperature *β*:

10 $$P\left({a|}\mathrm{RL}\right)=\sigma \left(A\left(a\right)|\beta \right)$$

Of note, this agent can be seen as a special case of our CHASE model that uses RL as learning rule and has $\kappa =0$ (that is, equivalent to *k* = 0).

#### Fictitious play (FP)

A more sophisticated approach that acknowledges the agency of the other player is given by fictitious play (FP)^27^. Here agents try to estimate the probabilities with which the other player chooses their actions and then best respond to them. This agent corresponds to a CHASE model as formulated above with $\kappa =1$ (that is, equivalent to a fixed *k* = 1). Both FP and RL are fully specified by two free parameters (a learning rate $\alpha$ and a behavioral temperature parameter $\beta$).

#### Experience-weighted attraction (EWA)

As there is evidence for both RL and FP in many games, the experience-weighted attraction (EWA) model was introduced to hybridize the two. In brief, this is achieved by introducing a parameter $\delta$ that quantifies the extent to which an agent also learns from ‘foregone’ payoffs (that is, the payoffs that would have resulted from choosing different actions). In addition, there are two more parameters that govern the extent to which old information is discarded—a forgetting rate that is fixed and cognitively determined, $\phi$, and one that is strategic, $\rho$, to allow discarding old experience when needed, for example, in changing environments. In full, the update equation takes the following form:

11 $$A{\left(a\right)}_{t+1}=\frac{\phi \times {n}_{t}\times A{\left(a\right)}_{t}+\left[\delta +\left(1-\delta \right)\times {\bf{I}}\left(a\right)\right]\times \pi }{{n}_{t-1}}$$

where $n$ captures the amount of experience (or the ‘experience-equivalent’) of the agent, which in turn is updated by:

12 $${n}_{t+1}=\rho \times {n}_{t}+1$$

As for the other models, attractions are converted into action probabilities based on a softmax function. The original formulation has five free parameters ($\delta ,\phi ,\rho$ as well as initial attractions and experience-equivalent). However, to ensure stable parameter estimates, we only estimated parameters that govern the updating process, while we manually set the initial values for attractions and experience-equivalents (as we did with all other models—zero for experience-equivalents, uniform for action frequencies and expected rewards based on random opponent behavior for value estimates), resulting in three free parameters.

#### Self-tuning EWA

As the EWA model has been criticized for its high number of free parameters, a simplified version that fixes some parameter values to empirical values and replaces others with functions of experience has been proposed^28^. In particular, a change-detector function $\phi (t)$ and an attention function $\delta (t)$ change how strongly the agent depreciates old evidence and how much she learns from foregone payoffs, respectively. As a result, only the behavioral temperature parameter $\beta$ is estimated. We refer to the original publication for a full description of all the changes to standard EWA.

#### ToMk

The model that is conceptually most similar to CHASE is the ToMk model, which was previously also used to capture strategizing in RPS^45^. In brief, this model also entails simulating what opponents of increasing sophistication would play, but uses a heuristic confidence-updating mechanism to form a response, rather than the more principled Bayesian BUs on which the CHASE model is based.

Specifically, the level-0 agent is defined as noisily responding to recency-weighted past action frequencies of the opponent (which are updated according to equation 1; similar to a level-1 agent in CHASE):

13 $$\mathrm{EV}\left({a|k}=0\right)=\varPi \times A{\left(a\right)}^{\mathrm{Opp}}$$

14 $$P\left({a|k}=0\right)=\sigma \left(\mathrm{EV}\left(a{|}k=0\right)|\beta \right)$$

where $\mathrm{EV}$ denotes the expected value of performing a particular action.

The next-higher agent, level 1, simulates the opponent’s level-0 behavior, computes their most likely action (by taking the argmax) and then responds with a mixture response that combines the best response to this opponent prediction with the expected value from their ‘own’ level-0 strategy:

15 $$\begin{array}{l}{P(a|k=0)}^{\mathrm{Opp}}=\left\{\begin{array}{l}\begin{array}{ll}1, & \mathrm{if}\,a=\max\,\mathrm{EV}{(a|k=0)}^{\mathrm{Opp}}\end{array}\\ \begin{array}{ll}0, & \mathrm{otherwise}\end{array}\end{array}\right.\end{array}$$

16 $$\mathrm{EV}\left({a|k}=1\right)=c\times \varPi \times {P\left({a|k}=0\right)}^{\mathrm{Opp}}+\left(1-c\right)\times \mathrm{EV}\left({a|k}=0\right)$$

Here the mixture weight *c* is given by a Markovian confidence variable that is updated on each trial according to another delta rule (that shares the update rate with the attraction delta rule):

17 $${c}_{t+1}=\left(1-\alpha \right)\times {c}_{t}+\alpha \times I\left(P\right)$$

where $I(P)$ is an indicator function that is 1 if the opponent action was successfully predicted and 0 otherwise.

Finally, higher levels simulate this mixture response process from the opponent’s perspective and then recursively integrate higher-level responses (to predicted argmax behavior) with a level-specific confidence according to equation (16) (replacing *k* = 1 and *k* = 0 with *k* and *k* − 1; and extending *c* to a vector of length *k*). These confidences all get updated simultaneously after observing the opponent action, but credit is only given to the lowest level that can explain the action (that is, the confidence for this level is updated positively; while the confidence for all higher levels that predict the same action stay constant; and all other levels decay):

18 $${c}(k)_{t+1}=\left\{\begin{array}{ll}(1-\alpha )\times {c}_{t}+{\alpha} {\times} {\rm{I}}(P), & {\mathrm{if}}\,\,\,P(a={a}^{\mathrm{Opp}}|{k}^{\mathrm{Opp}})\ne 1,{\forall}\ {k}^{\mathrm{Opp}} < k \\ {c}(k)_{t}, & {\mathrm{otherwise}}\end{array}\right.$$

where ${a}^{\mathrm{Opp}}$ is the action chosen by the opponent.

To avoid increasingly nested recursion, the confidence weights of the opponent are not estimated but are assumed to be fixed to 0.8 (similar to the assumption in CHASE that agents update beliefs, but opponents play a fixed level)^45^.

### Behavioral analysis

#### Statistical analysis

To analyze the behavioral data, we estimated logistic or linear regression models for participant-level analyses, and multilevel models with random intercepts and all random slopes included for trial-level analyses, using MATLAB’s Statistics Toolbox. To minimize the risk of false positives, we used the Satterthwaite approximation to estimate the degrees of freedom of fixed effects (rather than relying on the more liberal residual degrees of freedom approximation). The data distribution was assumed to be normal, but this assumption was not formally tested.

#### Model fitting and comparison

To fit the models to our participants’ behavior, we used maximum-likelihood estimation by combining an initial grid search with MATLAB’s fminunc function and applying transformations where necessary. We fitted one set of parameters per participant (across opponents) for each model and reset all relevant prior belief variables (for example, beliefs, attractions) to a uniform distribution at the beginning of each block. Notably, the model is therefore agnostic to whether a participant rematched with a previous opponent or encountered a completely new opponent. We computed Akaike information criterion scores to account for differences in the number of free parameters and used random-effects Bayesian model comparison to ensure that the comparison is not overly sensitive to outliers, using the VBA toolbox^46^. We report PXP, quantifying the likelihood that a model is expressed more frequently than all other candidates, accounting for the possibility of chance differences.

#### Model and parameter recovery

To confirm that the conclusions drawn from parameter estimates and model comparisons are valid, we conducted model- and parameter-recovery analyses. To account for parameter dependencies, we used empirical parameter estimates (from the fMRI dataset 2e) to simulate synthetic data (while competing against our artificial opponents). For the model recovery analysis, we simulated and fitted data using all candidate models and performed random-effects Bayesian model comparison for the data generated from each model. For each generated dataset, we report how often each candidate model provided the best fit for individual simulations (that is, based on parameters from a single participant). For parameter recovery, we simulated and fitted data only for the CHASE model (again using parameter estimates from dataset 2e) and correlated the parameters used for simulation with the estimates derived from fitting the simulated data.

### Neuroimaging data acquisition and analysis

#### Data acquisition

While participants performed the task in the scanner, we acquired T2*-weighted whole-brain echo planar images using a Philips Achieva 3T whole-body scanner (Philips Medical Systems) equipped with an 8-channel Philips sensitivity-encoded (SENSE) head coil. We used a TR of 2238 ms and TE of 30 ms with 40 slices (transversal, ascending acquisition); 3-mm slice thickness; 3-mm × 3-mm in-plane resolution; 0.5-mm gap; 90° flip angle. Five dummy-image excitations were performed and discarded before functional image acquisition started. In addition, we acquired a high-resolution T1-weighted three-dimensional fast-field echo structural scan used for image registration during postprocessing (sequence parameters = 170 sagittal slices; matrix size = 256 × 256; voxel size = 1 × 1 × 1 mm; TR/TE = 8.3/3.9 ms). In addition, we recorded physiological data during scanning to control for heart and breathing artifacts.

#### Data preprocessing

Preprocessing was performed using fMRIPrep (v20.2.3; ref. ^47^), which is based on Nipype (v1.6.1; ref. ^48^), with standard settings. For a detailed description, see the Supplementary Methods. For smoothing, we used a Gaussian kernel with a full width at half maximum of 6 mm.

#### Data exclusion criteria

To ensure that all participants engaged in strategic play rather than resorting to random gameplay, we assessed their behavior against the artificial opponent of lowest sophistication (*k* = 0). Because this opponent shows a simple tendency to repeat past actions, any attentive player should be able to successfully adapt to them over time. Accordingly, participants who did not perform above chance against this opponent were excluded from the analysis; this affected only two participants, leaving a final sample of *n* = 48.

#### Univariate analysis

To test associations between the model variables and neural activity, we used a mass-univariate approach as implemented in SPM12 (ref. ^49^). We combined all model-derived variables of interest into a shared first-level model that contained the following regressors: action selection phase, outcome phase, choice value (CV) during the action selection phase, and action-prediction error (APE) and BU during the outcome phase (see below for details and parametric control variables). Action selection phase and outcome phase were modeled as appropriately placed stick functions, whereas CV, APE and BU were modeled as parametric regressors of these stick functions (without orthogonalization). All regressors were convolved with the canonical hemodynamic response function in SPM12. To control for potential confounds of movement, we added the six motion parameters (three rotations and three translations), their derivatives and the volume-wise global signal estimate from fMRIPrep as regressors of no interest. We also included 18 regressors based on the software package TAPAS^50^ (vR2018.1.1) to control statistically for the effects of cardiac and respiratory cycles. To increase the sensitivity of our analyses, we restricted the analyses to a set of a priori selected ROIs based on an automated meta-analysis for the term ‘theory (of) mind’ (using Neurosynth’s uniformity test with the default *P*_FDR_ < 0.01 cutoff, including 181 studies as of 16 September 2022). We retained only connected clusters with *k* > 50 to remove noise from the mask. To assign clusters to anatomical regions, we used spectral clustering to break apart connected areas (for example, TPJ/medial temporal lobe or vmPFC/dmPFC) and anatomical information from automated anatomical labeling to put the correct ones back together again and assign labels^51^. Unless specified otherwise, we used nonparametric cluster-level inference within the conjunction of all ROIs using SnPM (http://warwick.ac.uk/snpm; v13.1.06) with an initial cluster-forming threshold of *z* = 2.408 and a family-wise error (FWE) rate of *P* = 0.05.

#### Parametric modulators

To test whether there is univariate neural evidence for the most important variables predicted by the CHASE model, we included the following participant-specific time courses as parametric modulators in a shared first-level model for each participant (without orthogonalization), allowing the parametric modulators to compete for variance in an unbiased fashion.

##### Choice value (CV)

When participants have to make a choice, we would expect to see neural activity in reward regions related to the estimated value of the chosen option, given their current prediction about the opponent’s next action. This value is constructed as part of the action selection process, namely in the very last step, when participants noisily choose a best response to their prediction of the opponent’s action, marginalizing over their beliefs. Formally, it is given by:

19 $$\mathrm{CV}=\varPi \times P\left({a|k}\right)\times B\left(k\right)\times {{\bf{I}}\left(a\right)}^{\mathrm{Partic}}$$

where ${\bf{I}}(a)$^Partic^ is 1 for the action chosen by the participant and 0 otherwise.

##### Action prediction error (APE)

Similarly, upon observing the action of the opponent, participants can compare their prediction against the observed outcome, leading to an APE. While this computation is not strictly required for the updating process of the model, it is highly likely to be computed by the brain, given the emphasis on trying to predict the opponent’s action; this signal can thus serve to evaluate the success of the current behavioral strategy. Formally, this APE is defined as the deviation from the player’s prediction:

20 $$\mathrm{APE}=1-P\left({a|k}\right)\times B\left(k\right)\times {{\bf{I}}\left(a\right)}^{\mathrm{Opp}}$$

where $I(a)$^Opp^ is 1 for the action chosen by the opponent and 0 otherwise. Please note that this signal differs from that investigated in previous studies of APEs^37,38^, as it measures deviations of observed actions from dynamically changing predictions based on the currently most likely level of opponent gameplay (based on adaptive mentalizing embedded in CHASE), rather than from just one static, fixed strategy as in previous studies.

##### Belief update (BU)

Finally, the most distinguishing feature of the CHASE model is that it assumes that people form and update beliefs about the level of recursive reasoning of the opponent. To test this, we can compute a BU signal that quantifies the extent to which participants updated their beliefs about the opponent upon observing the opponent’s action. Formally, we quantify this update from prior beliefs to posterior beliefs using the KL divergence:

21 $${\mathrm{BU}}_{t}={\sum }_{k}B{\left(k\right)}_{t}\times \log \frac{B{\left(k\right)}_{t}}{B{\left(k\right)}_{t-1}}$$

Because the KL divergence has no natural upper bound, this variable was *z* scored within participants before entering the first-level models.

##### Controlling for potential confounds

In addition to those variables of interest, we also added CV and the reward (that is, the outcome of a single round) as parametric modulators during the feedback phase to account for any reward-related processing. Finally, we added action identities (dummy-encoded) to control for potential confounds of (1) playing a certain action during choice, and (2) observing a certain opponent action during the feedback.

#### Functional connectivity analysis

Functional connectivity analyses were conducted using the CONN toolbox (v20b, release 22.v2407)^52^ in combination with SPM12. We focused on seed-based connectivity, using the rTPJ as the seed region and 15 predefined regions within the social brain network as targets (16 ROIs in total). These ROIs were selected a priori based on an automated meta-analysis for the term ‘theory of mind’ using Neurosynth’s uniformity test (see univariate analysis methods for details). Specifically, we tested whether individual differences in connectivity strength during the feedback phase—where participants received information about opponent strategies—were modulated by participants’ *γ* values, the key CHASE model parameter indexing sensitivity to opponent-level information. Our main goal was to determine whether individual variation in *γ* is reflected in the functional integration of the social brain network during outcome processing.

##### Denoising

Before running any analysis, functional data were denoised using a standard denoising pipeline^52^ including the regression of potential confounding effects characterized by white matter time series (five CompCor noise components), cerebrospinal fluid time series (five CompCor noise components), session and task effects and their first-order derivatives (six factors) and linear trends (two factors) within each functional run, followed by bandpass frequency filtering of the BOLD time series between 0.01 Hz and 0.1 Hz. CompCor^53,54^ noise components within white matter and cerebrospinal fluid were estimated by computing the average BOLD signal as well as the largest principal components orthogonal to the BOLD average within each participant’s eroded segmentation masks. In addition, we added BU as a nuisance regressor to control for trial-by-trial fluctuations in neural activity related to belief updating processes, ensuring that subsequent connectivity analyses reflected effects beyond moment-to-moment belief-driven variance.

##### First-level analysis

ROI-to-ROI connectivity matrices were estimated, characterizing the functional connectivity between each pair of regions among all 16 ROIs. Functional connectivity strength was represented by Fisher-transformed bivariate correlation coefficients from a weighted general linear model (GLM), estimated separately for each pair of ROIs, characterizing the association between their BOLD signal time series. Individual scans were weighted by a boxcar signal characterizing the feedback phase (the focus of this analysis), convolved with a statistical parametric mapping canonical hemodynamic response function and rectified.

##### Group level analysis

Group-level analyses were performed using a GLM. For each individual connection, a separate GLM was estimated, with first-level connectivity measures at this connection as dependent variables (one independent sample per participant and one measurement per feedback phase), and participant-level identifiers as independent variables. At the second level, individual $\gamma$ values were included as a covariate to test whether functional connectivity strength varied with individual differences in sensitivity to opponent-level evidence. Connection-level hypotheses were evaluated using multivariate parametric statistics with random effects across participants and sample covariance estimation across multiple measurements. Inferences were performed at the level of individual ROIs. Results were thresholded using a *P* < 0.05 connection-level threshold and a family-wise correction of *P*_FWE_ < 0.05 was applied.

#### Multivariate analysis

To introduce a tool to test the level of adaptive mentalization in other datasets and studies, we used a multivariate pattern analysis approach to test whether the levels of strategic sophistication, as well as the extent of the associated BU, can be decoded and predicted out of sample from neural activity. For the former, we used support vector machines as implemented in the Decoding Toolbox^55^ (for technical details, see Supplementary Methods). This was based on average run-level activity during either the choice or the feedback phases of the game. We fitted first-level models where we either included all trials within a run (and linked it to the level that was predominantly played during this run) or only trials where behavior can be linked to a particular level with certainty (based on a permutation distribution; we also added regressors for motion and physiological noise correction). For the continuous BU decoding, we used least absolute shrinkage and selection operator PCR as implemented in the CANlab Toolbox (https://github.com/canlab/CanlabCore; for technical details, see Supplementary Methods). We fitted another set of first-level models where trials are assigned to one of five separate regressors according to the extent of the BU (using equally spaced participant-specific bins of the log-transformed time series, again including regressors for noise correction). The resulting β maps were then averaged across runs, giving one β map per bin for each participant. To evaluate the model while minimizing the risk of overfitting, we performed a leave-one-participant-out cross-validation scheme for both types of decoding (that is, training the classifier on all but one participant and evaluating it on the left-out one), and performed permutation testing to compute nonparametric *P* values. For the categorical level decoding, we report balanced accuracy scores. For the continuous BU decoding, we report both average (using Fisher’s *z* transformation) and overall (that is, pooled across participants) Pearson correlation coefficients between the computational model-inferred extent of the BU and the one predicted from neural activation patterns.

#### Replication in an independent dataset

To test the robustness of our main findings, we repeated the corresponding neural analyses in an independent sample (*n* = 47; ‘Participants’). Date exclusion criteria (see above) were met by only one participant, leaving a final sample of *n* = 46. Univariate analyses followed the same procedures as above, but inference was restricted to the clusters that were identified in the primary dataset. Due to the more diverse demographics, we controlled for the potential effects of age and sex in second-level analyses. To assess the similarity across activation patterns beyond a binary statistical threshold, we also computed Pearson correlation between the distribution of second-level *t* values across the whole social brain. Multivariate analysis for continuous decoding also followed the same steps as above, but crucially did not entail training a new predictive algorithm. Instead, the pretrained neural signature from the primary dataset was used to predict the extent of the BUs in participants in this new sample.

### Reporting summary

Further information on research design is available in the Nature Portfolio Reporting Summary linked to this article.

## Online content

Any methods, additional references, Nature Portfolio reporting summaries, source data, extended data, supplementary information, acknowledgements, peer review information; details of author contributions and competing interests; and statements of data and code availability are available at 10.1038/s41593-026-02219-x.

## Supplementary information

Supplementary Information Supplementary Methods, Supplementary Results, Supplementary Note (Extended Discussion), Supplementary Figs. 1–18 and Supplementary Tables 1–14.

Reporting Summary

## Data Availability

All behavioral data can be accessed at: https://github.com/ruffgroup/neural_signature_of_mentalization^56^. Preprocessed neural data are available upon request.

## Code availability

The analysis code to produce all main results and figures can be accessed at: https://github.com/ruffgroup/neural_signature_of_mentalization^56^.
