# Supplementary material for: A neural signature of adaptive mentalization
Source: Nat Neurosci. 2026 Mar 9;29(4):934–44. doi: 10.1038/s41593-026-02219-x (PMC13061600; doi:10.1038/s41593-026-02219-x)
Supplement: Supplementary file 1 — Supplementary Methods, Supplementary Results, Supplementary Note (Extended Discussion), Supplementary Figs. 1–18 and Supplementary Tables 1–14. [file 41593_2026_2219_MOESM1_ESM.pdf]

nature neuroscience

Supplementary information https://doi.org/10.1038/s41593-026-02219-x

A neural signature of adaptive mentalization

In the format provided by the authors and unedited

Supplementary Information

Supplementary Methods

Datasets

In total, we recruited 506 subjects across 8 studies who together played 114'500 rounds of RPS across 2002 runs (i.e. 30-100 rounds against the same opponent; see Supplementary Table 1). In each run, participants were instructed that they face a new opponent. If participants played against artificial opponents (as opposed to other human participants), they always faced bots performing either 0, 1, or 2 steps of reasoning (always all three in counterbalanced order; in some cases twice or three times).

Supplementary Table 1: Description of the different datasets.

	Opponent	Actions	History	Zero-sum	Environment	Trials	Runs	*N*
1a	Human	4	Yes	No	behavioral	100	3	52
1b	Human	4	No	No	behavioral	100	3	58
1c	Human	3	No	Yes	behavioral	40	6	18
2a	Artificial	4	No	No	behavioral	40-80	3/6	194
2b	Artificial	3	No	No	behavioral	30	3	36
2c	Artificial	3	No	Yes	behavioral	30-60	3/6	62
2d	Artificial	3	No	Yes	behavioral	40	6	36
2e	Artificial	3	No	Yes	fMRI	40	6	50
								**506**

Supplementary Table 2: Description of the replication sample.

	Opponent	Actions	History	Zero-sum	Environment	Trials	Runs	*N*
3a	Artificial	3	No	Yes	fMRI	40	9	47

Identifying level-0 behavior

In models of recursive reasoning, a crucial question is what defines the behavior of a level-0 agent (as this is the foundation that all higher levels respond to). This question is especially challenging in the context of repeated interactions and a non-transitive payoff structure, as

is the case in the repeated RPS under study here. We tackle this challenge by empirically testing a range of plausible level-0 behavior that are based on earlier models of strategic decision making in repeated interactions, and by fitting subject-specific parameters to capture individual differences in this process.

To this end, we compared a range of plausible candidates across the human-vs-human datasets, and confirmed our results in the remaining datasets where subjects played against artificial opponents (which themselves were based on this learning rule). In particular, we considered that subjects might track i) historical action frequencies, ii) experienced rewards, iii) both experienced and foregone rewards, or iv) a combination of action frequencies and rewards. Here, (i) and (ii) correspond to the underlying learning rule in fictitious play and reinforcement learning for a level-1 and -0 agent, respectively (see "Alternative models" in Methods for a description). Further, (iii) incorporates the main mechanism from EWA[1], i.e. learning from foregone payoffs by some fraction δ:

$$A(a)_{t+1} = A(a)_t + [\delta + (1-\delta)\cdot \mathrm{I}(a)]\cdot \alpha \cdot (\pi - A(a)_t)$$

using the same notation as in Methods. Finally, (iv) combines elements of frequency and reward learning by taking a weighted combination of the experienced reward and the action identity:

$$A(a)_{t+1} = A(a)_t + \alpha \cdot ([\delta \cdot \mathrm{I}(a)\cdot \pi + (1-\delta)\cdot \mathrm{I}(a)] - A(a)_t)$$

We employed a two-step procedure to identify the most likely learning rule: First, we performed a model recovery analysis for CHASE models based on the different candidate learning rules (and their respective best-fitting parameter estimates in the fMRI dataset), revealing that only frequency and reward learning can be identified unambiguously (see Supplementary Figure 1). Next, we used random effects Bayesian model comparison to compare these two candidates. This revealed that frequency learning clearly outperformed reward learning, both when pooling the data (PXP = 1) and when only considering the bot datasets (which were used for the fMRI analysis; PXP = 1 in pooled data, PXP > .99 in individual datasets). In human-human gameplay, however, there was evidence that a minority of subjects might employ reward learning instead (PXP = .93 in pooled data, range .49 - .81 in individual datasets; see Supplementary Figure 2).

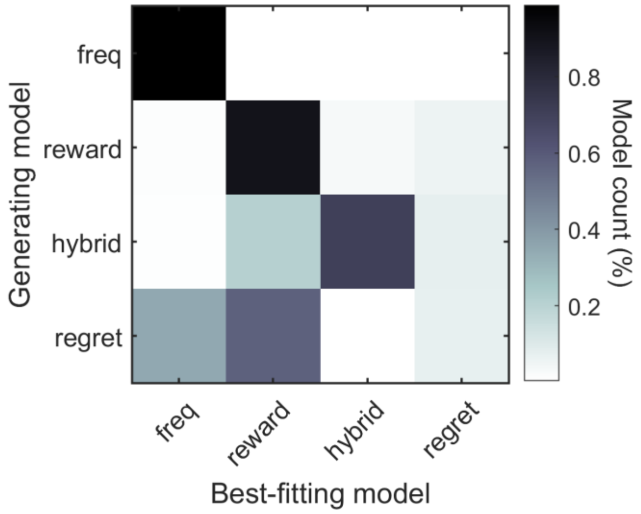

Supplementary Figure 1. Confusion matrix for the different learning rules from a model recovery exercise. Simulating synthetic data based on the models with different candidate learning rules based on their best-fitting parameter estimates (from the fMRI dataset 2e), and then performing model comparison on this artificial data, reveals that the simpler learning rules - action frequency and reward learning - can be recovered reliably, while the more complex ones - hybrid and regret-based learning - are often confused for the simpler ones.

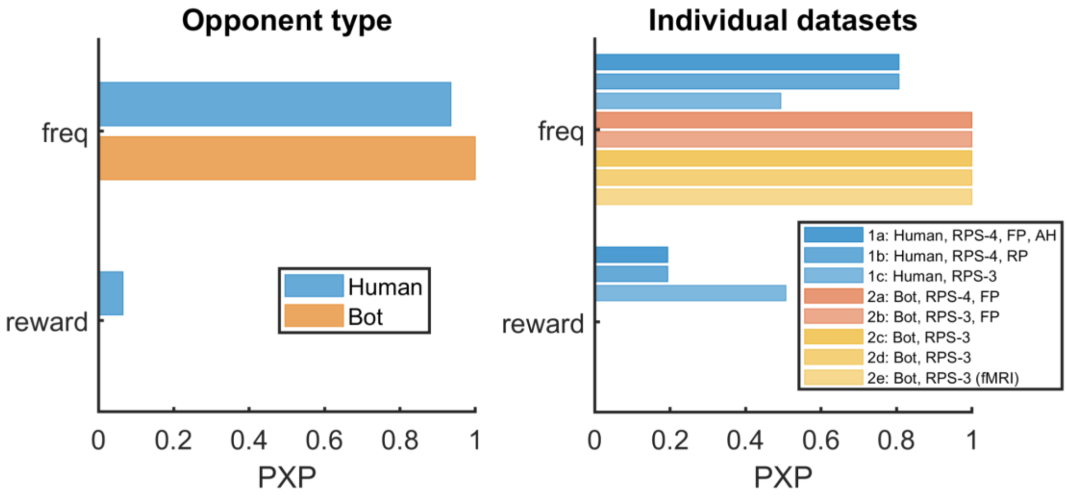

Supplementary Figure 2. Model comparison for recoverable learning rules of the CHASE model. Left panel: Protected exceedance probabilities (PXP) when pooling data by opponent type, right panel: PXPs for individual datasets. In datasets based on interactions with our artificial opponents, there is overwhelming evidence that frequency learning best describes the behaviour of participants (PXP = 1 in pooled data, PXP > .99 in individual datasets). In datasets based on interactions with other human participants, the evidence is also clearly tilted towards frequency learning, though some subjects might be better captured by reward learning (PXP = .93 in pooled data, range .49 - .81 in individual datasets).

Calibrating the artificial opponents

To make the artificial opponents as ecologically valid as possible, we based them on human behavior. In particular, they were based on a simplified version of the CHASE model where the level of sophistication is fixed to a particular level of reasoning throughout a game (i.e. k = 0, 1, or 2). To reduce working memory demands, we set the speed of updating attractions to a value close to 1, leading to a rapid decay of the influence that past trials have on the agent and thus a strong recency bias (i.e. $\alpha = .9$; 87th percentile in human-human gameplay). In addition, the recursive reasoning noise was set to a fairly low value (i.e. $\beta = 10$, 94th percentile in human-human gameplay) to maximize the information about the current level. To further mimic human gameplay, the noise level was adaptive and employed a win-stay/lose-shift heuristic. In particular, if the subject won in 3 out of 5 of the past trials *and* in the most recent one, the artificial opponent became increasingly likely to deviate from the prescribed action with increasing length of the winning streak. If the winning streak was 2 (or 3) against an artificial opponent with level 0 (or higher), a noisy action followed deterministically. Similarly, if one of the two other outcomes (i.e. lose or tie) was repeated three or four times, the artificial opponent would also play a noise action and deviate from its strategy. This prevented artificial opponents employing a self-referential strategy (e.g. k = 2) from becoming too predictable. Together, these features allowed the bot to provide a challenging and realistic opponent for human players.

Model fitting and parameter recovery

We estimated subject-specific maximum-likelihood estimates of all parameters using a combination of grid-search and an unconstrained nonlinear optimization algorithm as implemented in Matlab (fminunc). To ensure that parameter estimates stay within their natural bounds, we estimated the parameters either in logit (α) or log space (all other parameters except κ). As κ can only take on integer values, we repeated the optimization for all possible values of κ. Note that low values of κ prevent the effect of some of the other parameters, effectively removing them from the model (in particular: γ if $\kappa < 2$ and λ if $\kappa < 1$).

In addition, to avoid numerical issues and increase psychological plausibility, we had to set an upper bound on the value of the sensitivity to level evidence parameter γ (as otherwise the level-evidence softmax would increasingly turn into an argmax). For the analysis in this paper, we set a very liberal bound of $\gamma \leq 10$, which impacts less than 7% of subjects of the final fMRI sample and has only negligible effects on the log likelihood of the model (less than 0.01% decrease as compared to an unbounded model). However, while parameter recovery

of all but one parameters is very consistent across the examined range, recovery of γ shows a decrease in identifiability at $\gamma > 2.5$, suggesting that the effect of γ on the softmax function already starts saturating at this point (see Supplementary Figure 4; note that 77% of subjects are estimated to have a lower γ in the fMRI sample). If one is interested in exact parameter estimates rather than model predictions, future work might consider further constraining the estimation. For example, constraining γ estimates to $\gamma \leq 2.5$ still decreases the log-likelihood only marginally (~0.2% decrease on average in affected subjects; see Supplementary Figure 4) and doesn't change the inferred belief update trajectories significantly (average subject-level correlations r = .86 in affected individuals, individual correlations r > .8 in 94% of fMRI participants; see Supplementary Figure 5).

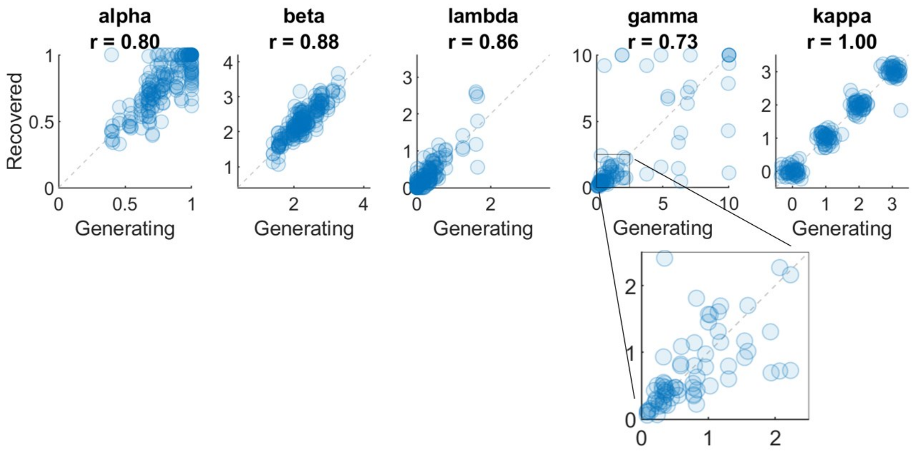

Supplementary Figure 3. Parameter recovery for the CHASE model. Synthetic data were simulated using the empirically observed parameter estimates from the fMRI sample (copied for each level of κ), and the estimated parameters from that data were compared to the generating parameters used for simulations. All parameters can be recovered well, with correlations between generating and recovered parameters of $r \geq .73$. The only noticeable difficulties with recoverability are observed for high values of γ (> 2.5), but these are only found in a minority of participants (77% of fMRI subjects had smaller values). Each dot represents a single simulation with parameters from one of the fMRI participants, in combination with a random κ from 0 to 2); the dotted gray line indicates the identity line.

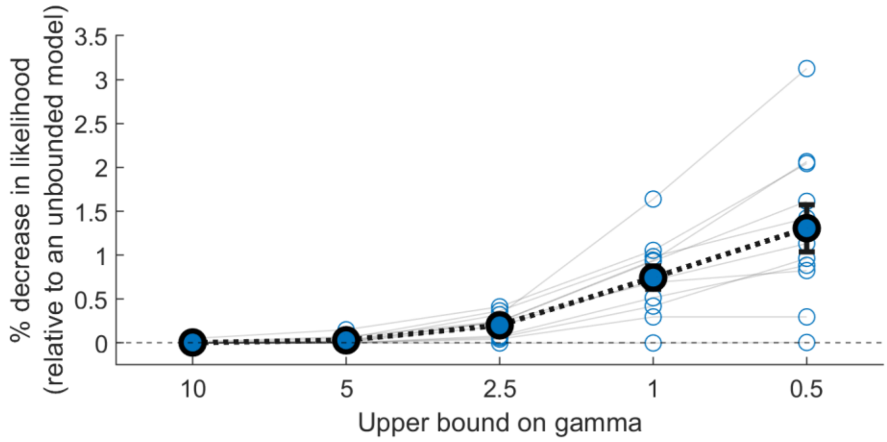

Supplementary Figure 4. Effects of increasingly smaller upper bounds of the estimation of the γ parameter. Shown is the proportional decrease (if any) in subject-specific likelihoods relative to an unbounded model (for subjects whose γ was estimated to be > 2.5 in the fMRI dataset 2e). For $\gamma \leq 10$ the likelihoods are essentially unchanged (<0.01% decrease), indicating that the effect of the parameter on the softmax function beyond that point (for $\gamma > 10$) has saturated (i.e., effectively turning it into an argmax). Further decreasing the upper bound to $\gamma \leq 2.5$ still only decreases the likelihood marginally (~0.2%) in affected individuals. Empty dots connected by lines are individual subjects, filled dots and error bars indicate mean and s.e.m., respectively.

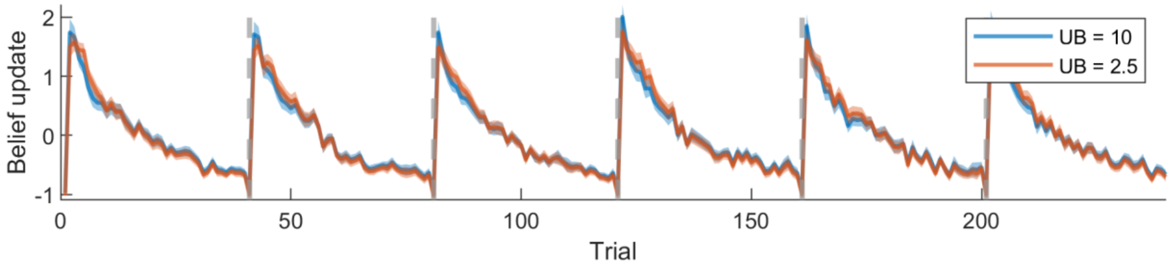

Supplementary Figure 5. Effects of decreasing the upper bound on the estimation of the γ parameter on the inferred belief update. The belief update time course (as entered into the neural GLMs) only changes marginally if decreasing the bound from 10 to 2.5. Plotted is the mean time course across subjects in the fMRI dataset 2e (shaded area indicates s.e.m.). Dotted vertical lines indicate a change in opponent. UB = upper bound.

Posterior predictive checks

To visualize what specific behavioral variance indicative of adaptive mentalization is captured by the CHASE model, but not the competing models, we performed posterior predictive checks of all models. As a complement to a purely quantitative model comparison, this serves to refute alternative models based on the presence or absence of key qualitative features in the data (i.e., depending on whether or not these features are also present when simulating data based on the candidate models)[2]. To this end, we used a simple model-independent

index of level gameplay that is purely based on the relation between the present and the previous action.

Specifically, we exploited the clear action-to-level mapping of RPS and combined it with the simple assumption that participants only care about the very last action. This leads to clear action predictions for each level, as illustrated in Figure 1b in the main text: For example, if both players played paper in the last trial, playing paper again would be indicative of *k*=0, scissors of *k*=1, and rock of *k*=2. More generally, if players did not play the same action in the previous trial, even higher levels can be distinguished (i.e., up to *k*=5; see "Identifiability of levels in RPS games" below). Note that this simple model-free index of level-gameplay corresponds to a strategic agent that fully updates attractions (i.e., α = 1) and is noiseless (i.e., $\beta \rightarrow \infty$). This allowed us to test if adaptive mentalization results in an increase in the frequency with which we observe the behavioral signature of the correct level against a particular opponent (i.e., always exactly one level higher). To explain this in more detail, we first formally define the model-independent signature of level k that we used for posterior predictive checks and then present a step-by-step example illustrating this procedure.

Model-independent signature. Formally, this model-independent signature can be defined for a level-1 agent as an action a_t that is a best response to the last action of the opponent with:

$$a_t^{self} = (a_{t-1}^{other} mod(3)) + 1$$

where mod(3) refers to modulo 3 in modular arithmetic, which is essential for mapping the action onto a circle and ensuring cyclic responses (i.e., rock beats paper beats scissors which in turns beats rock; but here using numbers instead, where 3 beats 2 beats 1 which in turn beats 3). Similarly, a level 2 signature is defined as a best response to an opponent who employs a level 1 strategy; accordingly, a level 2 signature corresponds to:

$$a_t^{self} = ((a_{t-1}^{self} + 1) mod(3)) + 1$$

Here, the plus one within the brackets captures the fact that the opponent is responding to one's own action (as a *k*=2 agent assumes the other to be *k*=1), while the plus one at the end captures one's own response to the predicted opponent action. As a consequence, this behavior is self-referential, since level 2 strategies are built on exploiting biases that arise from how one is perceived by others. Applying the same logic to a level 3 signature leads to:

$$a_t^{self} = ((a_{t-1}^{other} + 2) mod(3)) + 1$$

where two steps are added in the brackets, capturing the two lower (implied) levels, and the reference is again one's own past action.

Note that these best responses to given levels, based on recursive reasoning, represent the bare minimum for strategizing in this game. They do not incorporate any of the crucial assumptions of the CHASE model — that is, they only describe best-responding to observed last actions and neither incorporate any of the CHASE model's parameters - such as α (frequency learning rate), γ (inference over levels), or κ (maximum level) - nor the actual level inference based on cognitive hierarchy considerations. However, if participants do engage in successful mentalization, we can expect to see the frequency of the correct response to a given opponent increase over time.

Example calculation.

Step 1 — Action-to-level mapping. Suppose that in the previous round, both players chose action $a_{t-1} = 1$ (player A's last action = 1; player B's last action = 1). A level-1 player expects the other to repeat their last action and hence best-responds to the opponent's last move, which means "move one step forward"—so Player A would choose action $a_t(k=1) = 2$. A level-2 player assumes the opponent is level-1 (who would choose 2) and best-responds to that anticipated move, i.e., one more step forward to their action, $a_t(k=2) = 3$. A level-3 player assumes the opponent is level-2 (who would choose 3) and best-responds again, circling back to $a_t(k=3) = 1$. In this example, if the subject actually chose action $a_t = 2$ on the current trial, we tag the trial as matching level 1.

Step 2 — Calculate per-trial frequencies and 95% CIs (two complementary approaches).

(i) Correct vs. incorrect. First, in a model-agnostic approach, for each trial *t* we computed the proportion of subjects who played the unique best response to the bot's level (i.e., exactly one level above), pooling all other choices as "incorrect." This approach requires no disambiguation because each trial has a single correct label by design. We then smoothed this curve with a moving window of 10 trials to even out idiosyncratic changes across time (applied identically to empirical and simulated data) and computed mean and 95% CIs for both empirical and simulated data. This is the metric used in Figure 2d and for cross-model comparisons in Supplementary Figure 6.

(ii) Level-specific. In a second level-specific approach, we computed for each trial *t* and level $k \in \{1,2,3\}$ the proportion of subjects whose choice matched the level-*k* signature in that given trial (based on the logic and equations above), and then smoothed this curve with a moving window of 10 trials to even out idiosyncratic changes across time (applied identically to empirical and simulated data; see Supplementary Figure 7). To avoid double-counting in

the case that an action could theoretically correspond to multiple levels (~30% of all cases, note that in the correct vs incorrect approach, double counting is not possible, as there is only 1 correct answer per trial), we counted such ambiguous actions towards the level of the previous trial (and, if this was also ambiguous, randomly assigned a level). We then computed the across-subject mean $f_k(t)$ and its 95% confidence interval (computed across subjects). Both approaches yield very similar trajectories, indicating that the underlying strategies are well captured regardless of whether responses are summarized by specific levels or by the correct-vs-incorrect index.

Step 3 — Generate model predictions and compare. Then, to test if our model can recreate the patterns observed in participants' data, we used the model to create synthetic "twin" data for each participant, by using the participant's best-fitting parameter estimates to simulate their presumed decision-making when facing the artificial opponents (i.e., rather than using the data to estimate parameters, we used best-fitting parameters to simulate what choice data would look like given those parameters). For this synthetic data, we then assigned each action to a level and computed frequency time courses in the same way as for the empirical data. That is, we repeated Steps 1 and 2 on these synthetic choices to obtain model-predicted $f_k(t)$ curves and their 95% CIs. Finally, for the posterior predictive plots, we overlaid empirical and simulated trajectories, allowing a direct visual check of whether the model qualitatively reproduces the observed temporal evolution of level-specific signatures over time.

Since our focus is on adaptive mentalization (i.e., correctly inferring and adapting to the other's strategy), for visual clarity we focused in our main analyses on the "correct vs. incorrect" approach. Thus, the figures show the clear distinction between signatures of the correct response (i.e., frequency of *k*+1) and of incorrect responses (i.e., frequency of any other *k*; see Figure 2d for CHASE, and Supplementary Figure 6 for the alternative models). These figures illustrate that only CHASE, but none of the alternative models, are able to reproduce the qualitative patterns seen in the empirical data across artificial opponents. For the winning CHASE model, in Supplementary Figure 7, we also provide more detail and plot all level-signatures individually against all opponents. This further confirms that the model also captures the choice frequency of actions in line with incorrect inference of other levels. Together, these posterior predictive checks complement and further elucidate the quantitative model comparison reported in the main text, suggesting that a lack of capturing

adaptation across opponent types is a key driver of the superior quantitative fit of the CHASE model.

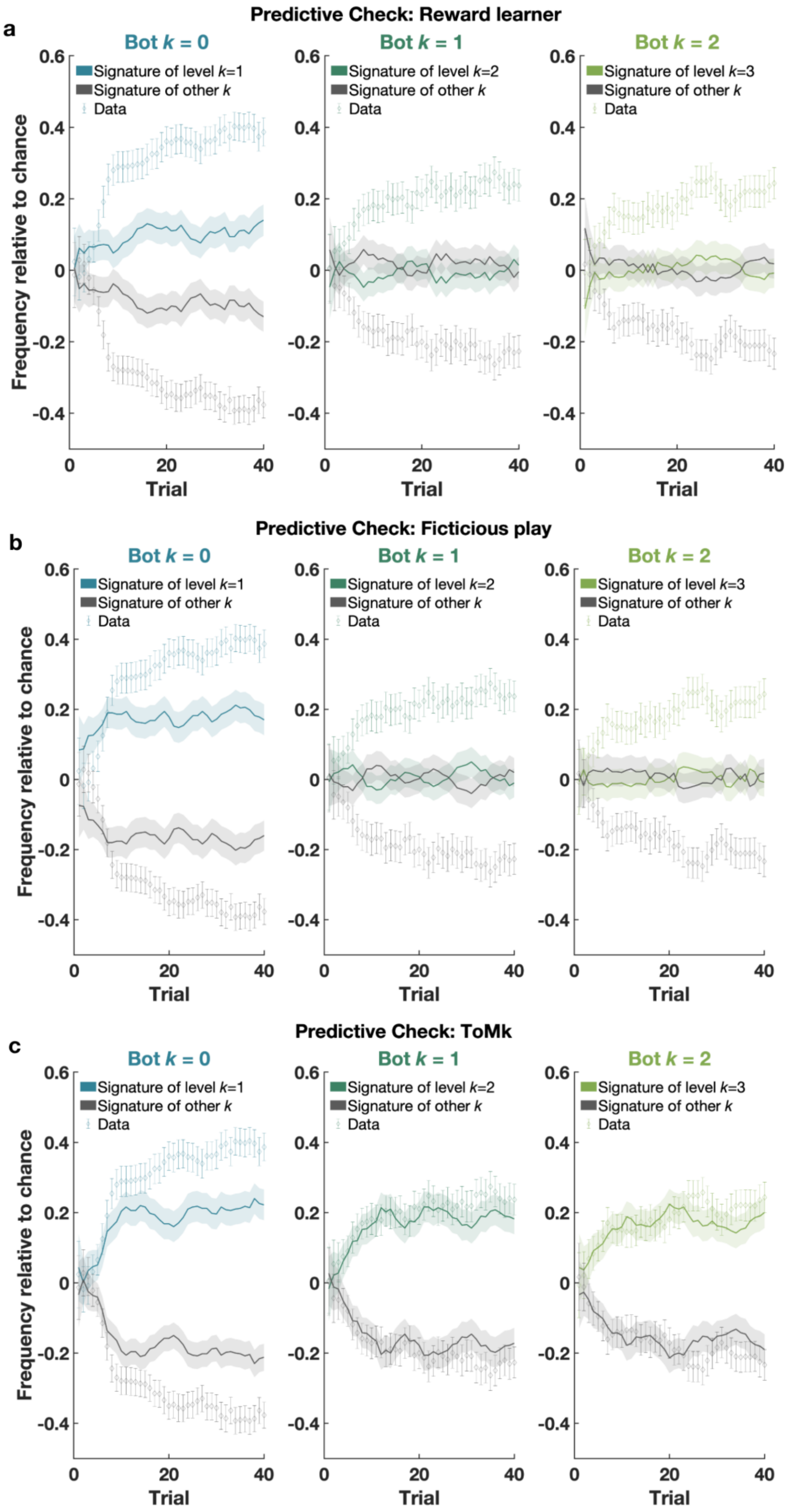

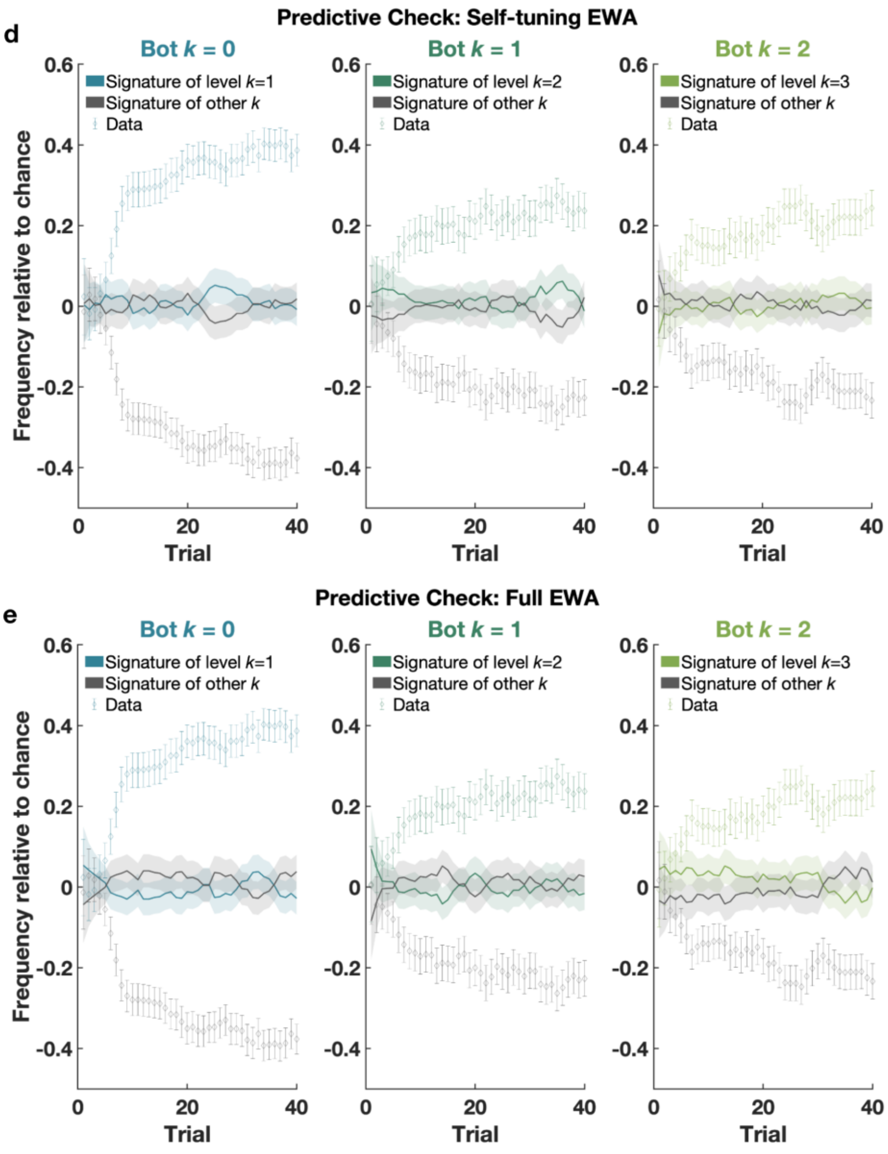

Supplementary Figure 6. Posterior predictive checks for all alternative models that were considered. Shown are the temporal evolutions of the mean frequency across participants (and 95% CI) of displaying qualitative signatures of stylized gameplay resulting from different levels of sophistication for both simulated data (as predicted by those models) and actual gameplay data from subjects, averaged using a moving window of 10 trials. The following models are displayed: (a) Reward Learning, (b) Fictitious Play, (c) ToMK-Model, (d) Self-tuning EWA and (e) Full EWA. Our results confirm that the predictions of the alternative models (solid lines) mostly fail to capture adaptive gameplay as observed empirically (error bar plots).

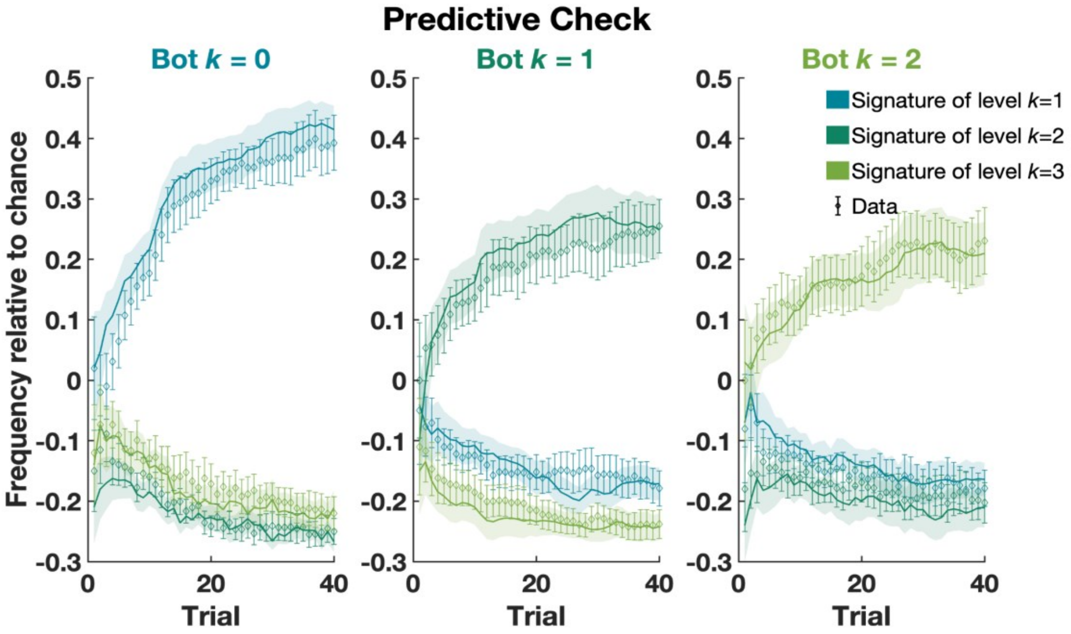

Supplementary Figure 7. Frequency of behavioral signatures consistent with level inference (levels *k*=1,2,3) for simulated (model-based) and empirical (human gameplay) data. As implied by level-*k* theory, participants show a clear increase in the qualitative signature of the level strategy that is exactly one step higher than the bot (i.e., *k*+1), while signatures of level strategies that are either higher or lower than that decrease. Synthetic data generated by the CHASE model successfully recapitulate this pattern. Behavioral signatures of strategy use were averaged across participants using a 10-trial moving window. If a given action was consistent with more than one level-k best-response signature, we applied a local history-consistent rule (examining the previous trial) to assign a unique level. The y-axis shows frequency relative to chance: $\Delta f(k) = f(k) - ⅓$; thus, zero denotes chance. Shaded bands indicate the model's posterior predictive trajectories (mean ± 95% CI); error bars show the corresponding empirical frequencies.

Imaging processing with fMRIPrep

Anatomical data preprocessing

Preprocessing was performed using fMRIPrep 20.2.3[3], which is based on Nipype 1.6.1[4]. The T1-weighted (T1w) image was corrected for intensity non-uniformity (INU) with N4BiasFieldCorrection[5], distributed with ANTs 2.3.3[6], and used as T1w-reference throughout the workflow. The T1w-reference was then skull-stripped with a Nipype implementation of the antsBrainExtraction.sh workflow (from ANTs), using OASIS30ANTs as target template. Brain tissue segmentation of cerebrospinal fluid (CSF), white-matter (WM) and gray-matter (GM) was performed on the brain-extracted T1w using fast of FSL 5.0.9[7]. Brain surfaces were reconstructed using recon-all in FreeSurfer 6.0.1[8], and the brain mask estimated previously was refined with a custom variation of the method to reconcile ANTs-derived and FreeSurfer-derived segmentations of the cortical gray-matter of Mindboggle[9]. Volume-based spatial normalization to one standard space (MNI152NLin2009cAsym) was performed through nonlinear registration with antsRegistration (ANTs 2.3.3), using brain-extracted versions of both the T1w reference and the T1w template. The following template was selected for spatial normalization: ICBM 152 Nonlinear Asymmetrical template version 2009c[10] [TemplateFlow ID: MNI152NLin2009cAsym].

Functional data preprocessing

For each of the 6 BOLD runs per subject, the following preprocessing was performed. First, a reference volume and its skull-stripped version were generated using a custom methodology of fMRIPrep. A B0-nonuniformity map (or fieldmap) was estimated based on two echo-planar imaging (EPI) references with opposing phase-encoding directions, with 3dQwarp[11] (AFNI 20160207). Based on the estimated susceptibility distortion, a corrected EPI (echo-planar imaging) reference was calculated for a more accurate co-registration with the anatomical reference. The BOLD reference was then co-registered to the T1w reference using bbregister (FreeSurfer) which implements boundary-based registration[12]. Co-registration was configured with six degrees of freedom. Head-motion parameters with respect to the BOLD reference (transformation matrices, and six corresponding rotation and translation parameters) were estimated before any spatiotemporal filtering using mcflirt in FSL 5.0.9[13]. BOLD runs were slice-time corrected using 3dTshift from AFNI[11,13]. The BOLD time series (including slice-timing correction) were resampled onto their original, native space by applying a single, composite transform to correct for head motion and susceptibility

distortions. These resampled BOLD time-series will be referred to as preprocessed BOLD in original space, or just preprocessed BOLD. The BOLD time series were resampled into standard space, generating a preprocessed BOLD run in MNI152NLin2009cAsym space. Confounding time series were calculated based on the preprocessed BOLD: The global signal was extracted within the whole-brain masks. All resamplings were performed with a single interpolation step by composing all the pertinent transformations (i.e. head-motion transform matrices, susceptibility distortion correction when available, and co-registrations to anatomical and output spaces). Gridded (volumetric) resamplings were performed using antsApplyTransforms (ANTs), configured with Lanczos interpolation to minimize the smoothing effects of other kernels[14]. Non-gridded (surface) resamplings were performed using mri_vol2surf (FreeSurfer).

Many internal operations of fMRIPrep use Nilearn 0.6.2[15], mostly within the functional processing workflow. For more details of the pipeline, see the section corresponding to workflows in fMRIPrep's documentation.

Technical details on decoding algorithms

We employed established toolboxes to perform multivariate decoding, using default parameter settings without any hyper-parameter tuning to avoid the risk of overfitting (which is inherent in the limited training data sizes feasible with task-based fMRI). Here we provide additional details about these default settings of all toolboxes.

First, we decoded strategic sophistication, which we derived from the model-inferred beliefs that participants hold about the sophistication of their opponent. We did so either by assigning all trials within a run to the model that was predominantly played during the whole run, or by assigning only those trials to a level where model-inferred beliefs were sufficiently high to rule out chance findings (i.e. $p < .05$ based on a permutation distribution). We could therefore test if information about playing a particular level is encoded throughout the whole interaction with a given opponent, or only once participants have figured out the correct strategy. To decode the level *k,* we used Support Vector Machines (SVM) as implemented in The Decoding Toolbox (TDT[16]). In the background, this toolbox relies on the libsvm library and defaults to the standard L2-regularized linear C-SVC formalization, where the level of regularization is determined by a parameter *c* that is set to 1. No prior data reduction step is applied. While this in principle could result in a higher risk of overfitting, our procedure of training across subjects and evaluating the performance on held-out subjects ensures that any overfitting would - if

anything - decrease the accuracy, suggesting that our results constitute a lower bound on the possible decoding accuracy.

For the continuous belief update decoding, we used the LASSO-PCR algorithm as implemented by the canlab toolbox (https://github.com/canlab/CanlabCore). This function first performs an economy size SVD to identify the first n principal components, where n is equal to the number of samples (in our case: number of participants times number of bins). Then, it uses a stepwise algorithm (LARS) to assess the effect of increasingly high L1 regularization. By default, the function returns the unregularized model where the regularization parameter λ is zero, effectively turning the procedure into a standard PCR. We confirmed that non-zero regularization would not further improve our out-of-sample decoding metrics, to ensure that this default behaviour does not unduly limit the robustness of our findings. To this end, we performed hyperparameter tuning of the regularization parameter λ using nested cross-validation either i) within individual subjects or ii) with 5 folds (i.e. combining several subjects). Alternatively, we iii) ran the whole decoding with 5 folds (rather than LOO-CV). Neither of these procedures produced better generalization than the default behaviour of the function (i.e., slower average correlation between model-inferred and decoded extent of belief updates; both in the training sample based on LOO-CV, and in the completely independent replication sample; see Supplementary Figure 8).

For both variables, we used a leave-one-subject-out cross-validation scheme, meaning that the number of folds was equal to the number of subjects, with each model being trained on all but one subject and tested on the left-out one. In contrast to leave-one-run-out decoding, this minimizes the risk of overfitting since learning needs to average over subject-specific idiosyncrasies. For permutation testing, we repeated these procedures 5000 times for both variables while randomly permuting the labels within each subject and collecting the resulting performance metrics.

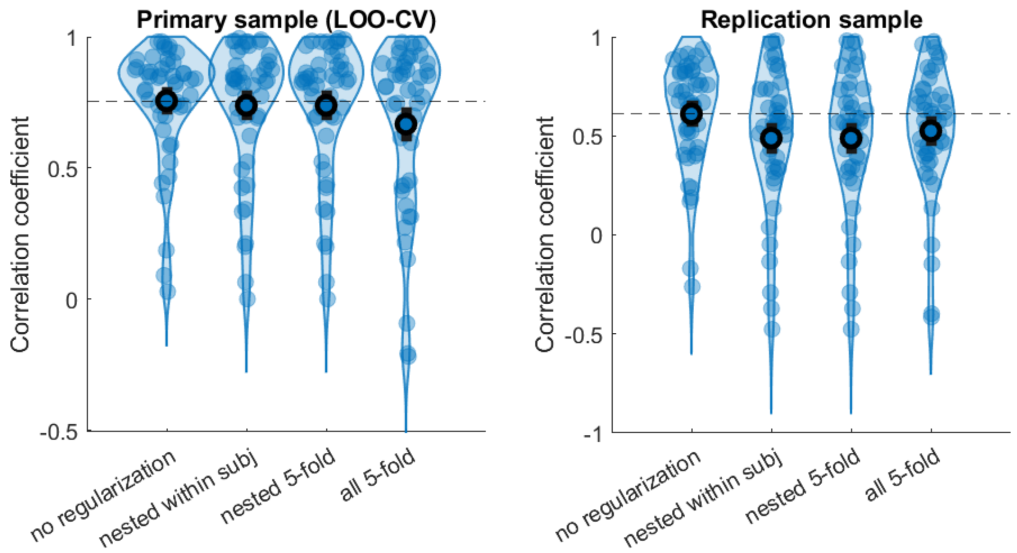

Supplementary Figure 8. Effect of different regimes of hyperparameter tuning to add regularization to the decoding model estimation. Both when considering generalization in the training sample (based on LOO-CV; panel **a**), and in the completely independent replication sample (panel **b**), the default model without regularization results in the highest out-of-subject decoding of belief updates, as assessed using the correlation between model-predicted and decoded extent of belief update. Dotted lines indicate the mean value of the model from the main text (with default settings of no regularization).

Analyses of the neural signature pattern

To identify the specific neural signatures of adaptive mentalization, we examined which voxels consistently contributed to predicting the belief update (BU) signal across all participants. To compute the multivariate activation pattern underlying out-of-sample BU predictions, we first calculated the dot product between each participant's observed BOLD responses to BU (across five bins, from 1 to 5) and the neural signature weights derived from the adaptive mentalization decoder. This approach yielded voxelwise activation values for BU, which, when summed, reconstruct the decoder's predicted BU. We repeated this procedure for all participants and then computed the slope of these five activation values for each individual, capturing how each voxel responded to incremental changes in BU. Finally, we converted the activation values to z-scores to identify the voxels that most consistently contributed to BU across participants. Figure 4d and Supplementary Figure 9 presents the thresholded z-mask (P < 0.001). This pattern reveals regions of interest (ROIs) exhibiting distinct negative contributions (e.g., the left superior temporal sulcus) as well as predominantly positive contributions (e.g., the bilateral anterior insula). We also observed a ventral-dorsal gradient in the rTPJ, with positive BU contributions in the dorsal parts, and negative BU contributions in the ventral part of rTPJ, as well as a complex pattern of positive and negative contributions in the dmPFC (please see Figure 4d in the main text). Please note that the patterns shown here are thresholded for illustration purposes, while the neural signature of adaptive mentalization predicts BU based on unthresholded weights.

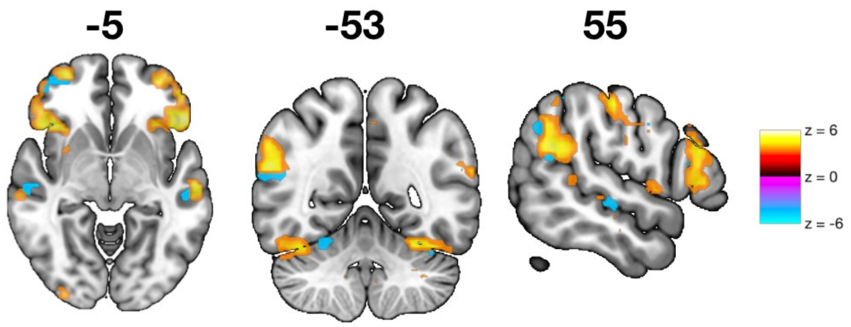

Supplementary Figure 9. The figure displays the multivariate activation pattern (identical to Figure 4d in the main text). This pattern reveals regions of interest (ROIs) exhibiting distinct negative contributions (e.g., the left superior temporal sulcus) as well as predominantly positive contributions (e.g., the bilateral anterior insula). We also observed an interesting ventral-dorsal gradient in the rTPJ, with positive BU contributions in the dorsal parts, and negative BU contributions in the ventral part of rTPJ. Please note that the patterns shown here are thresholded for illustration purposes (P < 0.001, uncorrected, whereas unthresholded patterns are used for prediction.

Supplementary Results

Natural distribution of played strategies

To examine the natural distribution of strategies employed during human-human interactions, we plotted the frequency of each level-k strategy (i.e., from strategic but non-adaptive players; see Methods) used by all participants in this condition (Supplementary Figure 10). The results indicate that while the majority of participants employed a level-1 strategy (35%), other strategies were also well-represented in the sample. Specifically, we observed level-0 strategies in ~15%, level-2 strategies in ~31%, and level-3 strategies in ~20% of interactions, suggesting that encountering diverse levels of reasoning is common in human interactions. Additionally, it is possible that some level-0 players might have intentionally adopted a Nash equilibrium strategy, as equilibrium play corresponds to a special case of level-0 gameplay in our model. The plot specifically illustrates the proportion of runs classified at each level k according to the CHASE model. Notably, in the RPS-3 variant, the distribution shifted slightly toward higher reasoning levels, with level-2 becoming the most common strategy (37%).

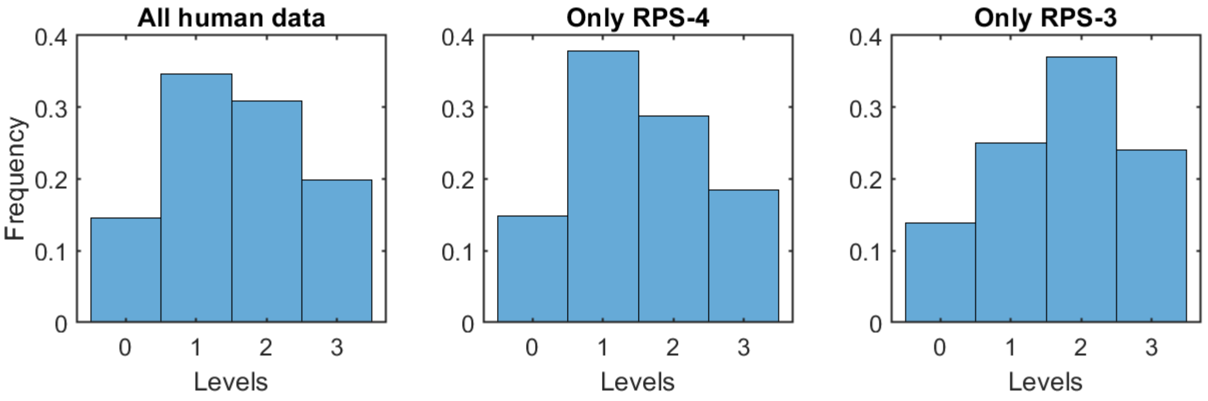

Supplementary Figure 10. Distribution of player types in human-human interactions. The plot shows the proportion of runs classified at each level of k in a simplified CHASE model with fixed level (k rather than κ), i.e., the opponent model in the full CHASE model. In line with previous work that typically relied on static levels, most participants were classified as level-1 or level-2 players (35% and 31%, respectively), though level-0 and level-3 play are observed as well (15% and 20%, respectively). In RPS-3, the distribution is slightly shifted to higher levels, with level-2 being the modal distribution with 37%.

Identifiability of levels in RPS games

An important conceptual question is at which level k the strategies in a three-action RPS game begin to repeat, potentially causing confusion between levels. While one might intuitively assume that strategies repeat at level k = 3 (due to the three possible actions), it

can be shown that strategy repetition and resulting confusion only arise at k = 6. Specifically, this stems from the fact that odd- and even-level agents respond differently — odd levels respond based on the opponent's previous action, whereas even levels respond based on their own previous action. Thus, as long as action distributions differ between players, strategies remain distinct for k<6. To illustrate, consider a scenario in which the Level 0 action for Player 1 is '2', and for Player 2 is '1' (see Supplementary Table 3). Under these conditions, distinct strategies repeat only after reaching Level k=6.

	Level 0	Level 1	Level 2	Level 3	Level 4	Level 5	Level 6
**Player 1 Action**	2	2	1	1	3	3	2
**Player 2 Action**	1	3	3	2	2	1	1

Supplementary Table 3: This example demonstrates that action pairs repeat only after reaching level k=6. Thus, counterintuitively, in a three-action Rock-Paper-Scissors game, the CHASE model can reliably differentiate strategies up to k=5.

Properties of the CHASE model

The effect of model parameters on performance. To examine the influence of specific CHASE model parameters on performance, we conducted a targeted simulation study. Given the model's complexity and the dynamic nature of the task, simulations are essential for rigorous interpretation, as CHASE parameters likely interact in non-linear and sometimes unpredictable ways. These interactions can bias performance in subtle ways that are not easily captured by simple correlational analyses. As such, simulations provide a more robust framework for isolating and understanding the functional roles of individual parameters and hence for interpreting individual differences in behavior and brain activity. Since the role of κ in determining strategic depth is conceptually straightforward, here, we focus on the parameters λ (loss sensitivity) and γ (sensitivity to level evidence), as their impact on performance is less clear a priori. To isolate their effects, we systematically varied either λ or γ across 500 simulated subjects while holding all other parameters constant (at their bayes-optimal or neutral values κ = 3, α = .5, β = 4,, so they would not interact non-linearly with all other parameters). This revealed that both parameters were systematically related to behavior, as described in the next paragraphs.

Loss sensitivity in the CHASE model. The observed λ parameter distribution, centered around approximately 0.5 in our data set, differs notably from values typically reported in the loss-aversion literature (~1.5). However, it is crucial to clarify that λ in our experimental paradigm does not directly reflect conventional loss aversion, as our task structure diverges significantly from standard risk-taking tasks. Specifically, within our repeated strategic interaction, low λ values effectively lead participants to treat losses as ties. This can yield a form of apparent "as-if" loss-seeking behavior—participants appear less sensitive to immediate losses not due to genuine loss-seeking preferences but rather as a strategic mechanism that enables exploration of alternative actions early within interaction blocks. Because immediate payoffs are linked to future outcomes through the learning component, low λ values may, for instance, reflect participants' strategic attempts to identify optimal winning strategies through initial exploratory play.

In our targeted simulation study, we observed that λ was negatively associated with performance (β = –0.74, t(498) = 24.55, P < 0.001), suggesting that lower loss sensitivity promotes greater exploration of potentially winning strategies and thereby higher behavioral success. This indicates that, in the context of our task, λ may function as a proxy for exploratory behavior, with participants treating losses less negatively in order to search more broadly for successful strategies.

Sensitivity to level-evidence and belief updating. The γ parameter in our model governs how quickly participants update their beliefs about their opponents, and thus we interpret γ as reflecting a participant's sensitivity to level-specific evidence. Higher γ values indicate that participants place stronger weight on evidence for the most likely level of opponent sophistication. In our targeted simulation study, γ was associated positively with performance (β = 0.34, t(498) = 8.08, *P* < 0.001), indicating that stronger integration of level-specific evidence facilitates faster belief updating and leads to better outcomes. Importantly, however, this positive relationship depended on correct assumptions about the opponent level: participants with κ = 0 who strongly but incorrectly weight the evidence (e.g., by mistakenly attributing the wrong level to an opponent), exhibit no relationship between γ and performance (β = -.0003, *t*(498) = -0.03, *P* = .98). Future research should further investigate the nuanced role of γ, particularly by examining whether clinical populations with deficits in Theory of Mind would also display lower levels of γ, and how it affects overall performance in such games.

Bayes optimal inference. Since the CHASE model utilizes a Bayesian inference approach, Bayes-optimal play against the artificial opponents corresponds directly to a special case of the CHASE model (i.e., matching parameters of the artificial opponent). Thus, within our framework, deviations from Bayes-optimal behavior are naturally captured by variations in participants' fitted parameter values. These parameters therefore reflect the extent to which each participant's gameplay departs from theoretically optimal Bayesian inference (given the artificial opponent). However, in the context of human-human interactions, optimal Bayesian inference strongly depends on the opponent's sophistication level and learning dynamics. Consequently, determining universally optimal Bayesian parameters in such interactions is not straightforward, as the optimal strategy varies directly with opponent strategies.

Whole brain analyses

While the focus of our analyses was on regions of the social brain network, we also performed whole-brain corrected analyses to test the robustness of our findings. We found that almost all of the reported results also survive whole-brain correction (only exceptions: PCC for the action prediction error, and vmPFC as well as the left TPJ/MTL for the belief update; see Supplementary Figure 11 and Supplementary Tables 4-9). Conversely, most of the clusters from the whole brain analyses were also detected in the analyses restricted to the social brain mask (additional regions that were significant at $p < .01$ were deactivations of parietal cortex for choice value, occipital cortex for action prediction error, and postcentral gyrus for belief updates). This further corroborates the key role that areas of the social brain network play during mentalization.

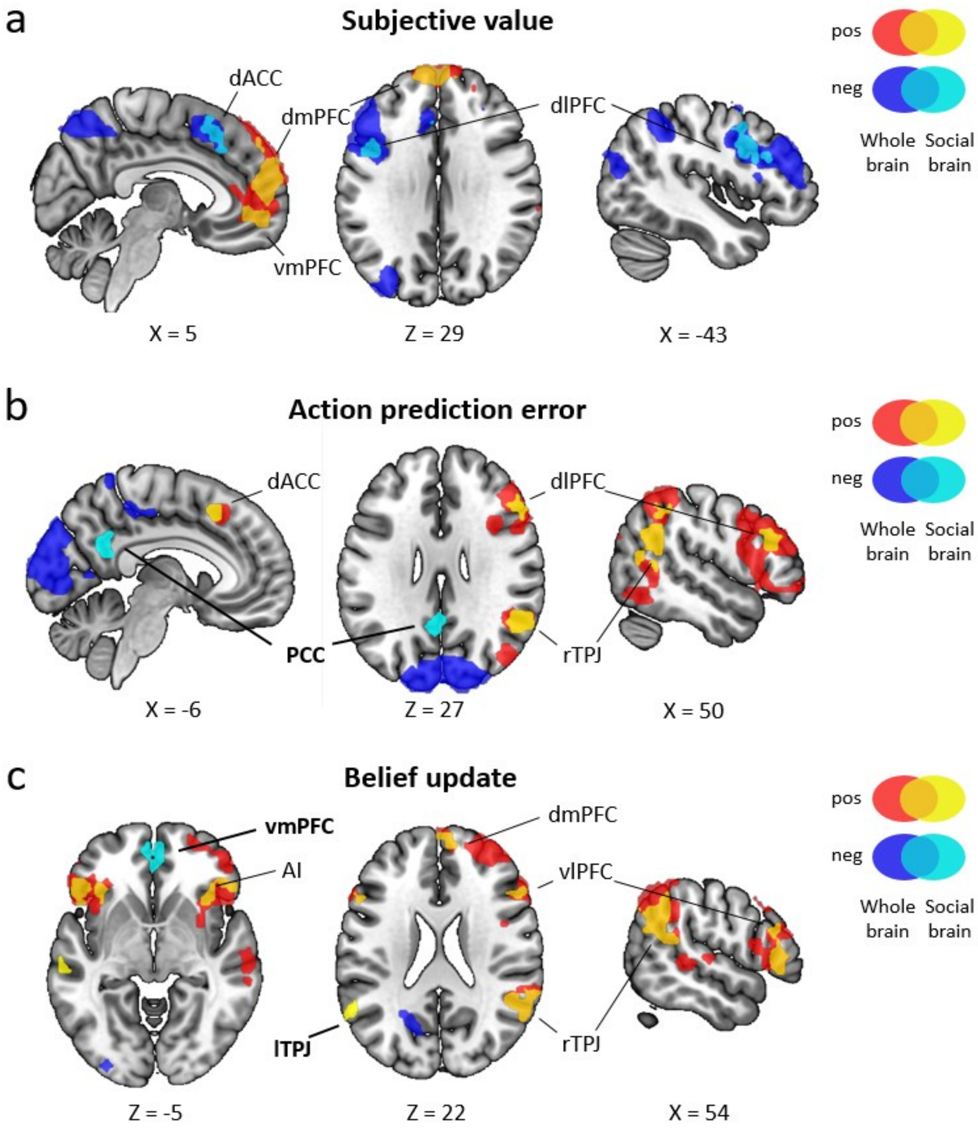

Supplementary Figure 11. Overlay of the results of applying i) whole-brain correction versus ii) small-volume correction (SVC) within regions of the social brain. Almost all regions and voxels identified in the SVC analysis survive whole-brain family-wise error correction; the only expectations are PCC for the action prediction error, and vmPFC and the left TPJ/MTL for the belief update (corresponding labels in bold).

Control analyses to assess neural overlap of APE and BU signals

One might expect APE and BU to be linked, given that surprising actions by the opponent might nudge subjects to adjust their beliefs. Moreover, we found that the activation clusters linked to APE and BU partly overlapped in the rTPJ (n = 106 overlapping voxels; representing 64% and 63% of voxels significantly linked to APE and BU, respectively). However, a mixed effects analysis did not reveal a statistically significant relationship between the two variables (β = -0.094, $t(39.5)$ = -0.72, p = 0.473), and within-subject correlations were generally low (i.e. $|r| < .3$ in 90% of subjects, and never $|r| > .5$). This indicates that any overlap between the associated neural activation patterns was not just trivially driven by correlations of the underlying variables. To further investigate if this neural overlap reflects similar or different neurons in this area representing the two computational processes, we tested if the strength of expressing APEs vs BUs was correlated across subjects (by extracting average betas in either the meta-analytically derived ROI, or their respective activation clusters). This did not reveal a significant relationship (p = 0.47 and p = 0.66, respectively), suggesting that the underlying neural populations are (at least partly) distinct and that the two neural activations constitute separable processes.

Control analyses to account for systematic trends in BU signal

As the BU signal - like any learning signal - on average tends to decrease during an interaction with an opponent (as the subject is reducing uncertainty about the other's strategy), one might worry that the neural signal revealed by our analyses instead reflects some other quantity that decreases monotonically over time. Thus, a potential concern is that (parts of) the corresponding neural activation may reflect general learning-related signals, such as novelty or a related quantity that decreases monotonically throughout a run. However, theoretical considerations and results of control analyses suggested that a substantial part of the neural activity indeed specifically reflects BUs. First, individual subjects rarely showed a monotonic decline of BU, with subjects on average exhibiting an increase in BU in almost half of all trials (i.e. 40%; s.t.d. = 9%; range 17% - 56%). Second, we fitted two additional sets of first-level GLMs where we added either a linear trend alone or a linear trend in combination with a quadratic trend as additional parametric modulators during the feedback period (with orthogonalization turned off, to allow the predictors to compete for variance). When using these GLMs to test for an effect of BU within the significant clusters from the main analyses, we still found significant activations in all clusters that were replicated across fMRI datasets (i.e.

bilateral TPJ and AI/vlPFC) in the models where both terms were added, and all but the left TPJ in the model where only a linear term was added. This confirms that our BU results cannot be explained by simple temporal trends but instead rely on the trial-specific signal that is captured by our model-derived belief updates.

Control analyses of the BU decoding

We can rule out several possible confounds of the BU decoding results (see also above for discussion of these points in the context of the univariate analyses). First, beliefs may be updated more strongly whenever subjects are surprised about the observed opponent action, so that the pattern identified here might reflect a surprise signal instead. Note that in our model, this type of surprise is explicitly captured by the APE, allowing us to test explicitly if the predictions of the decoder carry any information about the APE as well. However, when correlating the decoder predictions with the average APE per BU bin used for training, we found a non-significant correlation close to zero (i.e. $r = .03$; $p = .80$; see Supplementary Figure 18), suggesting that the multivariate pattern is not related to surprise. Corresponding control analyses also ruled out a link to rewards or reaction times (see Supplementary Figure 18). Second, since BUs generally become weaker over time when facing a specific opponent (though not monotonically; see above), one might worry that the signal may relate to another process that decreases over time, such as purely temporal adaptation. To address this, we regressed the decoder's predictions on both the model-inferred BUs and the average trial number (within that bin of BUs) to test if the decoder picks up on variance that goes above and beyond a monotonic decline. This revealed not only that the model-inferred BU remained highly significant ($p < 10^{-7}$), but that the non-linear component was roughly twice as large as the linear decline (i.e., standardized betas 0.36 vs. -0.18). The BU-related pattern was even robust to controlling for a quadratic effect of time, which left the estimate and significance of the model-inferred BU essentially unchanged ($\beta = .36$, $p < 10^{-5}$) but rendered both time predictors non-significant (linear effect: $p = .24$; quadratic effect: $p = .65$). These control analyses thus corroborate that the multivariate pattern specifically picks up and predicts the strength of adaptive mentalization-related belief updates.

Replication of main univariate and multivariate results

The out-of-subject predictive power of the multivariate neural signature indicates a remarkable consistency in the neural processes associated with adaptive mentalization. However, while

the leave-of-subject-out procedure that we employed strictly separates training and testing data, it might still overestimate the confidence in the effects[80]. In addition, our primary sample consisted of young male university students, raising questions about their generalizability. To address this question of ecological validity, and to corroborate our findings, we tested if our neural results replicate in a demographically more diverse group of participants (N = 47, 57% female, age 32 ± 8.2, years of education 16 ± 3.1, see Methods for more details).

First, we replicated the univariate neural analysis. This confirmed that most clusters that were significant at p(FWE) < .01 in the original sample were also significantly activated in this more population-representative sample (only exceptions: dlPFC for subjective value and left TPJ for belief update; analysis restricted to voxels previously identified to be implicated; see Figure 5a:c, Supplementary Tables 10-13, and Supplementary Figure 15 for individual data points). In line with this consistency between samples, we found that the spatial distribution of the second-level t-values across the social brain was highly correlated between the two samples, especially for the belief update signal, indicating that the neural activation pattern was also similar below the threshold for statistical significance (belief update: r = .72, action prediction error: r = .55, subjective value: r = .42; all p < 0.0001 based on permutation testing). Both of these analyses thus suggest that the neural correlates of the model-derived variables are robust and generalizable.

We then turned to the replication of the individual difference analysis of functional connectivity effects related to the parameter gamma (γ), which governs the individual speed of level-belief updating (BU). In our independent sample, we successfully replicated 9 out of the 11 initially significant ROI correlations ($p_{uncorrected}$ < .05), using the same SBC analysis approach with rTPJ as the seed region, outcome activity as the psychological context, and γ as a second-level covariate. Specifically, all significant regions in the discovery sample replicated, except for the left amygdala and the dorsal anterior cingulate cortex. These results confirm that functional network integration of the rTPJ, during the processing of outcome information about opponent strategies, is stronger in individuals with higher behavioral sensitivity to this information. Moreover, these results establish that across diverse samples, the network of areas the rTPJ communicates with - differentially in people with different sensitivity to level information - comprises bilateral anterior insula (AI), bilateral dorsolateral prefrontal cortex (dlPFC), bilateral medial temporal lobe (MTL), precuneus (PC), dorsomedial prefrontal cortex (dmPFC), and the left TPJ (see Extended Data Figure 1b).

Next, we tested if the identified multivariate neural pattern - the neural signature of mentalization - can be used to predict the belief update in this independent dataset. Note that in contrast to the univariate analyses, this does *not* entail fitting a new model and comparing the results, but using the pretrained model to predict the belief updates in this new sample. Despite the demographically more diverse composition of this sample, we found that the prediction performance only dropped slightly and remained at a very high level (average correlation predicted-actual belief update $r = .67$, overall correlation in pooled data $r = .60$, $p < .0002$ based on permutation testing, in 74% of participants $r > .5$). In line with this, a linear regression showed that age, sex, and education were not significantly linked to this prediction performance (age: $\beta = 0.210$, $t(41) = 1.47$, $p = .149$; sex: $\beta = -0.35$, $t(41) = -1.18$, $p = .247$; years of education: $\beta = -0.040$, $t(41) = -0.284$, $p = .778$), whereas IQ had a significant positive effect ($\beta = 0.432$, $t(41) = 2.788$, $p = .008$; estimated based on the two-subtest version of the WAIS-IV[81]). To provide further evidence for the generality of the pattern, we also performed these procedures in reverse order (i.e., training the decoder on the more diverse replication sample with mixed sex and more varying socio-economic variables and testing in the male-only, more homogenous primary sample). Reassuringly, this reverse decoding analysis did not result in systematic performance differences (the average cross-validated correlation dropped slightly from 0.82 to 0.74, while the average test sample correlation increased from 0.67 to 0.77). This generalizability shows that the multivariate neural signature is a sensitive and robust measure of adaptive mentalization that generalizes across different groups and demographics, suggesting that this neural marker holds potential for assessing adaptive mentalization in clinical applications.

Supplementary Figures and Tables

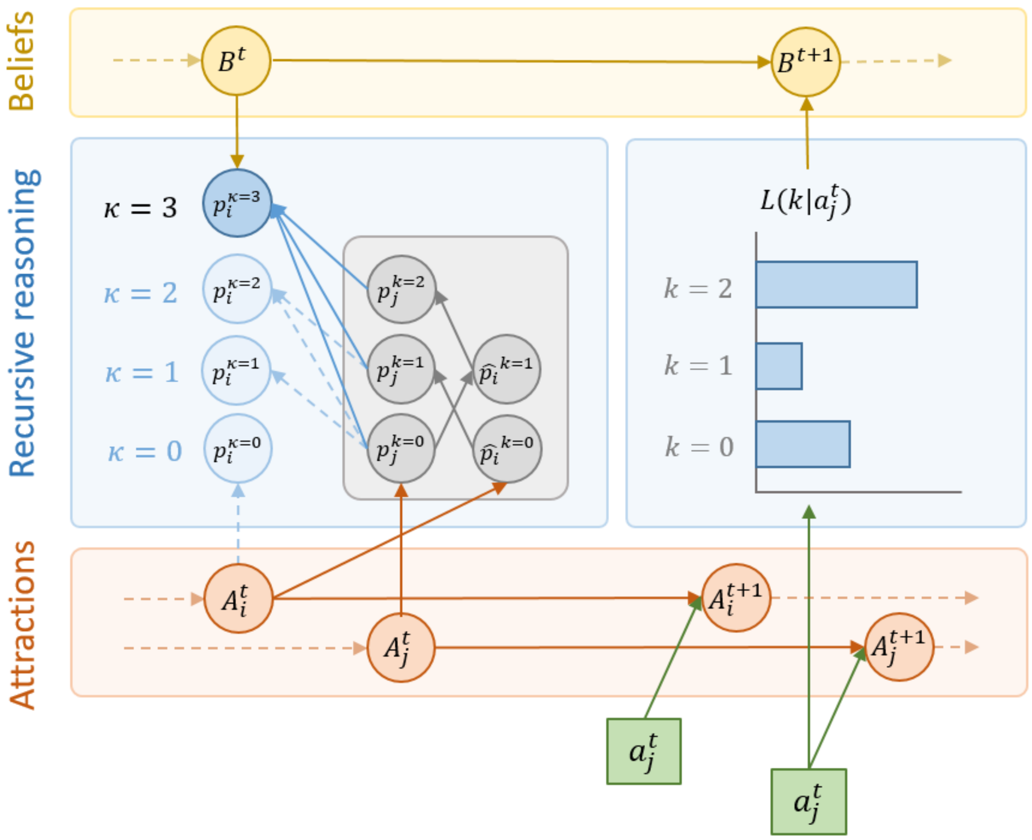

Supplementary Figure 12. Extended graphical model. For a high-level explanation, please refer to Figure 1c in the main text. Bottom: Attractions *A* are tracked for both players (*i* and *j*) and updated based on the observed actions *a*. Middle: Based on these attractions, strategic players (gray plate) form a finite number of recursive reasoning steps (determined by their level *k*) by iteratively best-responding to the predicted behavior of an opponent whose sophistication is exactly one level lower than their own (leading to a series of response probabilities *p(a)*; in the figure abbreviated with *p* for improved clarity). E.g. a level-0 player will act directly in line with his attractions, whereas a level-1 player predicts that her opponent plays a level-0 strategy (i.e. acting on *his* attractions) and responds to those, etc. (note that it follows that odd and even levels differ w.r.t. the attractions which they are based on). In contrast, adaptive players (blue plates) assume they are facing a strategic player and try to infer their level of sophistication. To this end, they simulate the reasoning process of a strategic opponent (gray plate) up to their own upper bound of sophistication κ, and use this to both i) create an integrated action prediction, weighted by their current belief about the opponent's level *B* (left blue plate), and ii) create a likelihood function over levels, once the opponent's action is observed (right blue plate). Top: Finally, they use this likelihood function to update their belief about the opponent's sophistication using Bayes rule. All of these latent variables (in circles) are inferred based on the observed actions (in squares). Arrows indicate information flow.

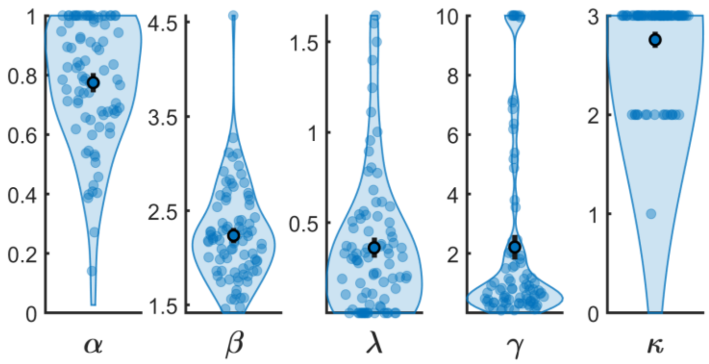

Supplementary Figure 13. Maximum-likelihood parameter estimates reveal substantial variability in the population, indicating individual differences in the capacity to mentalize (α = speed of updating attractions, β = recursive reasoning noise, λ = loss sensitivity, γ = sensitivity to evidence for opponent's level, κ = depth of mentalization ability; estimates from datasets 2d and 2e with final calibrated artificial opponents).

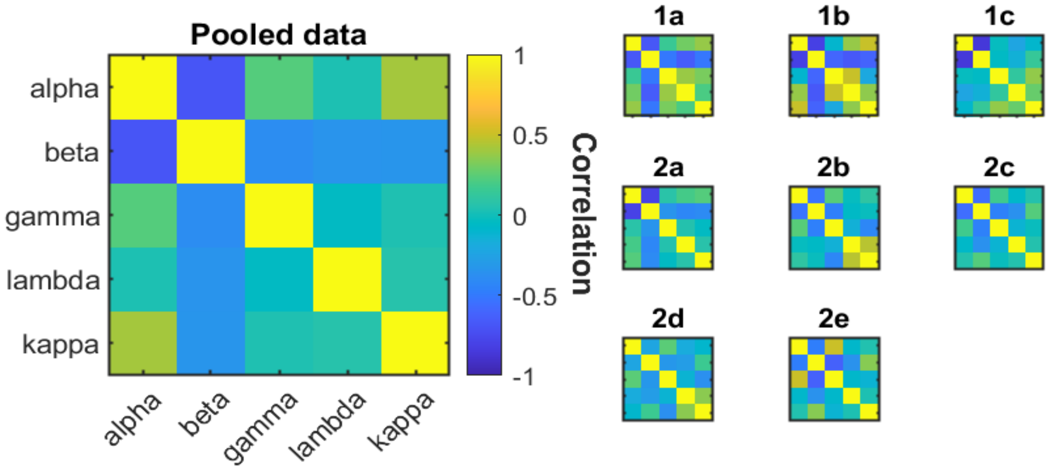

Supplementary Figure 14. Spearman correlations between parameter estimates. Correlations are mostly weak to moderate (i.e. |rho| ≤ .4), with the exception of α and β, which are negatively correlated with rho = -.69. These relationships are fairly stable across task parametrizations, as regressing the correlation coefficients per dataset on the different task parameters for which more than one dataset exists (i.e. presence of a focal point, number of actions, and type of opponent) did not reveal any significant effects at $p < .05$ (using Bonferroni correction to account for the multiple comparisons resulting from the number of parameter combinations). Conceptually, the negative correlation between α and β might be related to the fact that higher values of α lead to much more precise predictions (i.e. concentrating the probability mass on a single action), which might be compensated with a lower β (i.e. higher behavioural temperature). Left panel: Pooled data. Panels on the right: Individual datasets as defined in Supplementary table 1.

Supplementary Table 4. Significant clusters for the model-predicted choice value in the primary fMRI sample within a social brain mask.

ROI	cluster-level		voxel-level (peak)	x,y,z mm
	pFWE	*k*	*T*	
dACC	0.0082	97	6.38 (-)	-1, 12, 51
dlPFC (l)	0.0034	138	5.78 (-)	-31, 3, 58
vm/dmPFC	0.0002	336	5.19	3, 63, 27

Supplementary Table 5. Significant clusters for model-predicted action prediction errors in the primary fMRI sample within a social brain mask.

ROI	cluster-level		voxel-level (peak)	x,y,z mm
	pFWE	*k*	*T*	
AI (r)	0.0500	47	5.75	36, 24, -5
TPJ (r)	0.0012	167	5.08	48, -49, 27
dACC	0.0378	54	5.03	-7, 21, 48
dlPFC (r)	0.0472	49	3.77	45, 33, 20
PCC	0.0256	65	3.62 (-)	-1, -49, 20

Supplementary Table 6. Significant clusters for model-predicted belief updates in the primary fMRI sample within a social brain mask.

ROI	cluster-level		voxel-level (peak)	x,y,z mm
	pFWE	***k***	***T***	
vlPFC/AI (l)	0.0028	118	5.51	-46, 24, -5
TPJ (r)	0.0006	165	5.37	51, -43, 16
TPJ/MTL (l)	0.0022	125	4.72	-58, -46, 37
vlPFC/AI (r)	0.0036	116	4.45	39, 27, -2
dmPFC	0.0206	60	4.31	6, 51, 44
vmPFC	0.0208	60	4.29 (-)	-4, 45, -9

Supplementary Table 7. Significant clusters for the model-predicted choice value in the primary fMRI sample (whole-brain corrected).

Anatomical region (s)	cluster-level		voxel-level (peak)	x,y,z mm
	pFWE	***k***	***T***	
dACC/dlPFC/vlPFC (l)	0.0008	1471	6.38 (-)	-1, 12, 51
dlPFC (r)	0.0162	576	6.34 (-)	36, -1, 55
IPL (l)	0.0048	855	5.64 (-)	-40, -46, 41
vm/dmPFC	0.0058	800	5.19	3, 63, 27
MTL (r)	0.0444	375	4.34	48, -34, 6

Note. IPL = Inferior parietal cortex.

Supplementary Table 8. Significant clusters for model-predicted action prediction errors in the primary fMRI sample (whole-brain corrected).

Anatomical region (s)	cluster-level		voxel-level (peak)	x,y,z mm
	pFWE	***k***	***T***	
TPJ/MTL (r)	0.0146	793	6.24	60, -55, -2
Occipital	0.0034	1356	6.15 (-)	18, -97, 16
AI/vlPFC/ dlPFC/dACC (r)	0.0040	1247	5.85	27, 18, 51
SPL (bilateral)	0.0174	686	4.94 (-)	-22, -52, 72
m/pSTS (l)	0.0500	412	4.80 (-)	-46, -25, 20

Note. SPL = Superior Parietal Lobule; m/pSTS = medial/posterior Superior Temporal Sulcus.

Supplementary Table 9. Significant clusters for model-predicted belief updates in the primary fMRI sample (whole-brain corrected).

Anatomical region (s)	cluster-level		voxel-level (peak)	x,y,z mm
	pFWE	*k*	*T*	
Postcentral (l)	0.0016	789	9.01 (-)	-52, -28, 55
Cerebellum	0.0404	301	6.80	-28, -79, -30
AI/vlPFC/ dlPFC (r)	0.0004	1272	5.88	48, 9, 48
dACC	0.0266	339	5.56 (-)	-1, 6, 44
AI/vlPFC (l)	0.0422	295	5.51	-46, 24, -5
TPJ (r)	0.0106	502	5.48	60, -49, 48
Fusiform (l)	0.0378	289	5.44 (-)	-40, -73, -16
Cuneus (l)	0.0268	336	5.41 (-)	-7, -79, 41

Supplementary Table 10. Significant clusters for the model-predicted choice value in the replication dataset (within significant voxels from the primary dataset).

ROI	cluster-level		voxel-level (peak)	x,y,z mm
	pFWE	*k*	*T*	
dACC	0.0081	54	4.53 (-)	2, 18, 44
dlPFC (l)	0.0433	21	4.25 (-)	-42, 6, 26
vm/dmPFC	0.0345	23	3.62	-1, 56, 2

Supplementary Table 11. Significant clusters for model-predicted action prediction errors in the replication dataset (within significant voxels from the primary dataset).

ROI	cluster-level		voxel-level (peak)	x,y,z mm
	pFWE	*k*	*T*	
TPJ (r)	0.0156	27	3.85	48, -46, 48

Supplementary Table 12. Significant clusters for model-predicted belief updates in the replication dataset (within significant voxels from the primary dataset).

ROI	cluster-level		voxel-level (peak)	x,y,z mm
	pFWE	*k*	*T*	
TPJ (r)	0.0001	114	6.12	50, -46, 34
vlPFC/AI (r)	0.0010	66	5.61	50, 30, -8
TPJ/MTL (l)	0.0194	20	4.56	-58, -28, -2
vlPFC/AI (l)	0.0135	26	3.94	-42, 24, -12

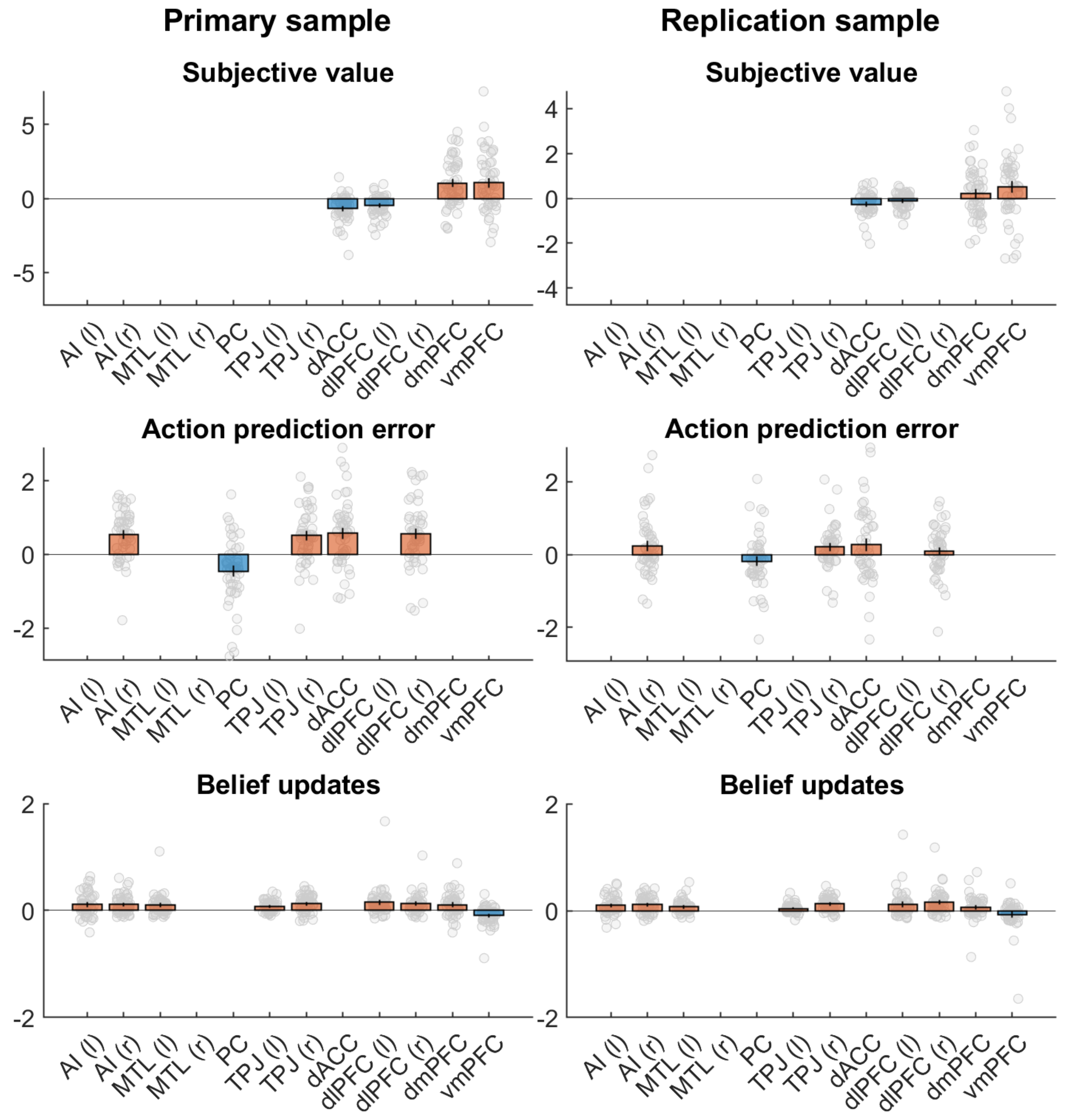

Supplementary Figure 15. Average neural betas in the two datasets within significant clusters from the primary dataset in the different regions-of-interest (ROIs; mean across subjects +/- s.e.m.; ROIs with no significant clusters are omitted). Betas from individual subjects are overlaid in grey (with some jitter). Left panel: Primary dataset. Right panel: Replication dataset.

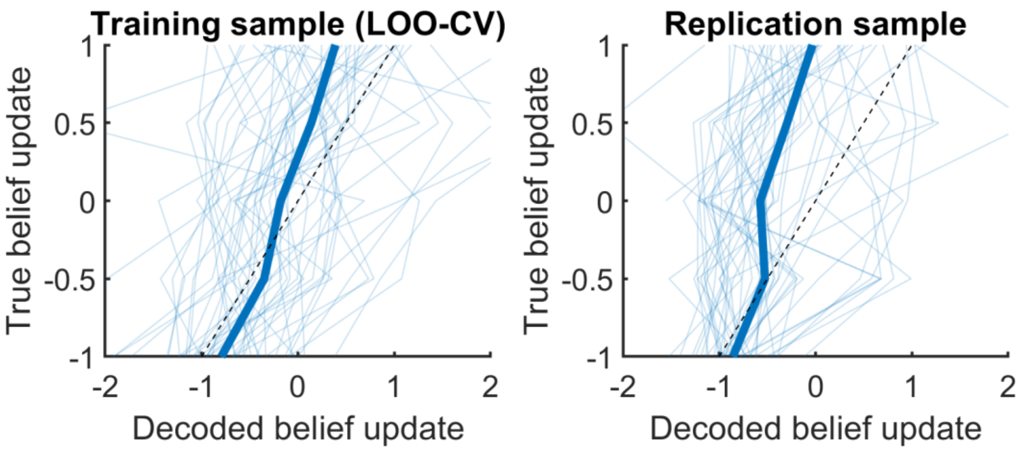

Supplementary Figure 16. Relationship between the ground truth belief updates (as computed by the model) and the bin-level out-of-subject predictions from the neural signature. As in Figure 4a, but blue lines are subject-level line plots for the prediction per bin (rather than regression lines), and the thick blue line is the average across subjects. The dotted black line represents ideal decoding.

Supplementary Table 13. Parcellation of weights of the neural signature of adaptive mentalization (only showing FDR-corrected weights based on bootstrapping; parcellation based on the AAL atlas with collapsed subregions for ease of presentation). The dot product between these weights and the neural betas gives the predicted extent of mentalization-related belief update; accordingly, positive weights contribute to a larger predicted BU while negative weights do the opposite. To give a sense of overall contribution as well as average effect, we report both the total absolute weight (i.e., the sum over all unsigned weights) and the mean weight per region. Shown are regions with 10 or more significant voxels; for ease of presentation, weights are multiplied by a factor of 1e4.

**Region**	**voxels (pos, neg)**	**total abs. weight (pos, neg)**	**mean weight (pos, neg)**
Cerebellum	465 (419, 46)	4350 (3797, 553)	7 (9, -12)
Frontal	356 (341, 15)	3562 (3449, 113)	9 (10, -8)
Postcentral	356 (82, 274)	3096 (705, 2391)	-5 (9, -9)
Occipital	334 (98, 236)	3024 (771, 2253)	-4 (8, -10)
Temporal	311 (274, 37)	2525 (2174, 351)	6 (8, -9)
Calcarine	237 (213, 24)	2132 (1861, 272)	7 (9, -11)
Precuneus	157 (71, 86)	1434 (690, 743)	0 (10, -9)
Precentral	141 (0, 141)	1376 (0, 1376)	-10 (0, -10)
Parietal	108 (3, 105)	1009 (24, 986)	-9 (8, -9)
Rolandic	93 (1, 92)	903 (9, 893)	-10 (9, -10)
Cuneus	83 (65, 18)	807 (588, 219)	4 (9, -12)
SupraMarginal	79 (43, 36)	657 (354, 303)	1 (8, -8)
Supp Motor Area	77 (2, 75)	859 (18, 842)	-11 (9, -11)
Insula	75 (58, 17)	720 (559, 161)	5 (10, -9)
Angular	22 (18, 4)	166 (129, 37)	4 (7, -9)
ParaHippocampal	18 (18, 0)	406 (406, 0)	23 (23, 0)
Cingulum	13 (2, 11)	148 (21, 127)	-8 (11, -12)

Supplementary Table 14. Parcellation of FDR-corrected weights resulting from retraining the decoder of adaptive mentalization in the replication sample. Presentation format as in Supplementary Table 13; for ease of presentation, weights are multiplied by a factor of 1e4.

Region	voxels (pos, neg)	total abs. weight (pos, neg)	mean weight (pos, neg)
Temporal	317 (301, 16)	3669 (3453, 217)	10 (11, -14)
Frontal	254 (239, 15)	2936 (2756, 180)	10 (12, -12)
Precentral	189 (5, 184)	2580 (45, 2535)	-13 (9, -14)
Angular	128 (2, 126)	1664 (22, 1642)	-13 (11, -13)
Precuneus	119 (119, 0)	1690 (1690, 0)	14 (14, 0)
Supp Motor Area	111 (6, 105)	1494 (73, 1421)	-12 (12, -14)
Cerebelum	95 (54, 41)	1226 (658, 568)	1 (12, -14)
Occipital	49 (1, 48)	540 (13, 527)	-10 (13, -11)
Cingulum	43 (9, 34)	586 (112, 474)	-8 (12, -14)
Paracentral	38 (20, 18)	533 (243, 291)	-1 (12, -16)
Caudate	37 (37, 0)	507 (507, 0)	14 (14, 0)
Vermis	34 (34, 0)	850 (850, 0)	25 (25, 0)
Parietal	26 (26, 0)	345 (345, 0)	13 (13, 0)
Calcarine	22 (16, 6)	358 (283, 75)	9 (18, -12)
Thalamus	16 (12, 4)	232 (175, 57)	7 (15, -14)
Hippocampus	13 (0, 13)	162 (0, 162)	-12 (0, -12)

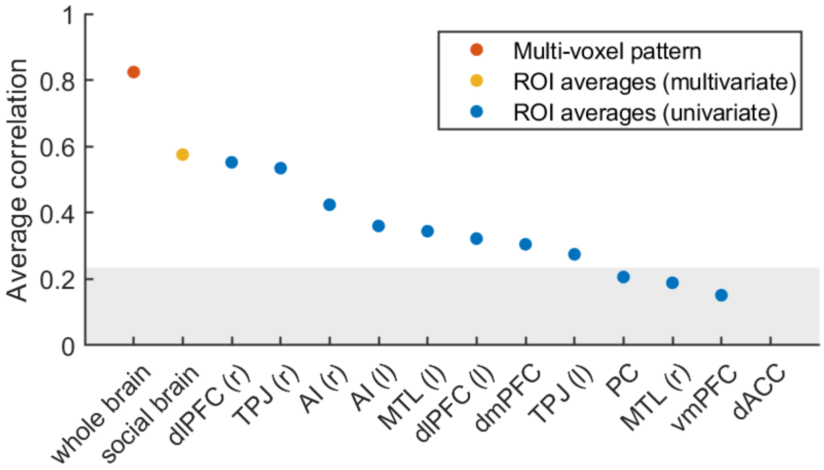

Supplementary Figure 17. Assessing the importance of multivariate patterns over and above average activity per ROI. Plotted is the mean out-of-sample correlation between the decoded and the actual extent of the belief update from a leave-one-subject-out cross-validation procedure for different models. Note that the value for dACC is omitted, as it is negative (-0.50) but its absolute value is smaller than the positive value for other ROIs, hence not affecting our conclusions. The shaded area indicates chance (based on permutation testing). The models are either based on multi-voxel patterns (in red) or mean activity per ROI, for different individual ROIs (in blue) or their combination (in yellow).

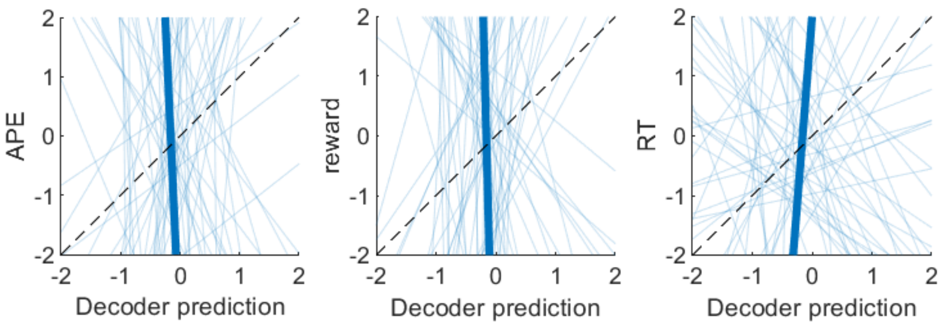

Supplementary Figure 18. The predictions from the neural signature of adaptive mentalization are not related to model-predicted action values (p = .84), experienced rewards (p = .82), or response times (p = .14). Figure format as in Figure 4a in the main text.

Extended Discussion

Relation to existing models of adaptive mentalization. The CHASE model extends previous accounts of adaptive mentalization in three ways. First, as previous models were either tailored to binary games[21] or more complex grid worlds[5,10], we extend the scope of this model family to Rock-Paper-Scissors (RPS) (see [45] for a similar approach). Second, in place of complex update equations[21] or value function approximation[5,10], we provide a simple and general formalism that allows capturing these belief updates in a straightforward way, by combining a best-response function with Bayes rule. Third, we address a key issue in models of recursive reasoning: the question of what constitutes non-strategic gameplay, which recursively defines the behaviour of all higher levels. While existing accounts utilized a fixed non-strategic gameplay strategy, we determined this empirically by comparing several plausible candidates. As a result, the CHASE model provides a tool for inferring how people learn about others' strategies, and thus a principled approach to investigate the neural mechanisms underlying fully adaptive mentalization in strategic interactions.

Conceptual interpretation of level-0 gameplay. It is important to note that within the CHASE model (or any related level-k model), level-0 gameplay does not have to correspond to actual actions of unstrategic participants but rather to a mental construct that higher-level players (k>0) use in their recursive reasoning process. That is, the model does not require the empirical existence of level-0 players, but rather captures what participants believe a non-strategic agent would do. Nevertheless, the CHASE model is able to identify level 0 gameplay, as evidenced by the parameter recovery (Supplementary Figure 3). Moreover, we tested common variations of level-0 assumptions (e.g., reward learning, regret learning) and found that the frequency learner—which updates probabilistic action tendencies based on observed frequencies—provided the best fit to participant behavior.

Level-0 gameplay and parameter recoverability. While our CHASE model remains robust across the empirically observed parameter space, certain edge cases warrant clarification. Our parameter recovery analysis (Supplementary Figure 3) demonstrates that the model can successfully recover and characterize meaningful behavior across the whole parameter range observed in the fMRI dataset. That said, as with virtually all computational models of social behavior, parameter recovery in the CHASE model can be difficult when behavior is extremely noisy or no learning takes place—such as for very high inverse decision noise (β) or very low learning rates (α). These limits to reliable parameter estimation when decision-making

becomes very stochastic are a general problem of all choice and learning models. However, our recovery analyses show that such theoretical edge cases do not impact the parameter estimates obtained from our fMRI dataset, supporting the robustness and general applicability of the CHASE model for capturing realistic human strategic reasoning.

Types of adaptiveness in adaptive mentalization. Our model assumes a Bayesian belief system that updates dynamically on a trial-by-trial basis to adapt to idiosyncrasies of a particular opponent. While it was tested extensively in human-human interactions (datasets 1a:c, N = 127), where opponents could freely change their strategies across trials, the fMRI paradigm used artificial opponents with static levels. In other words, while participants in the human-human experiment were free to adapt their strategies or beliefs throughout the interactions, in the fMRI studies we primarily investigated how participants form and update beliefs about opponents who follow different strategies, but do this consistently across time (rather than how they adapt to opponents that dynamically change their strategy throughout a single interaction). Accordingly, the adaptiveness that we investigate here is primarily concerned with adapting to changing opponents, rather than adapting to dynamically varying opponents. We made this design choice deliberately for the fMRI experiment to address several methodical constraints imposed by fMRI methodology. That is, we needed to (i) ensure balanced exposure to different sophistication levels, (ii) establish a clear ground truth for model validation, and (iii) optimize scanning efficiency. Although opponent strategies were fixed within each block (40 trials), participants were unaware whether they were facing the same or a different opponent when rematched, requiring them to continuously update their beliefs. While this represents a key form of adaptive mentalization, real-world settings might entail additional complexity as the opponent's strategy may evolve dynamically within a single interaction (though note that in the level-*k* framework employed here, this would only affect behaviour of agents with $\kappa \geq 2$; see Discussion in the main text). Future research could explore modifications to the task, such as introducing fully adaptive artificial opponents, to further investigate how participants track and respond to within-opponent changes over time. However, in our human-human interactions, where opponents could freely change their strategies, our CHASE model still provided the best fit compared to all available alternatives, showing that it can successfully capture flexible strategic reasoning in competitive interactions with dynamically adapting opponents.

That said, we emphasize the substantial methodological advantages provided by our approach of using carefully calibrated artificial opponents. First, employing artificial opponents allows researchers to systematically balance the distribution of opponent types across different experimental conditions. This effectively eliminates sampling biases commonly encountered in natural human-human interactions and, in turn, may reduce the number of false positives in the literature. Second, the use of well-defined artificial opponents strongly reduces noise in the data related to variability in opponent strategies, thus enhancing statistical power and increasing the sensitivity of experimental analyses. Moreover, artificial opponents can be further fine-tuned by systematically manipulating critical model parameters such as the learning rate (α) or sensitivity to level evidence (γ), enabling researchers to rigorously investigate how these parameters of the bot may influence human responses. For instance, future studies might adjust these parameters to create opponent strategies that are suitable for specific participant populations, such as children or patients with memory impairments. In summary, the high experimental control over bot strategies offered by the CHASE framework not only improves methodological rigor but also provides novel opportunities to customize experiments according to the specific cognitive and clinical characteristics of participant groups.

Ecological validity. Apart from extending the CHASE model to other types of games, it currently does not explicitly incorporate (a) emotional states (or inferences about emotional states), (b) social norms, or (c) group dynamics, such as interactions involving multiple players. Since many social interactions naturally involve these components to varying degrees—such as feelings of betrayal or fairness considerations—we argue that emotional states as well as social norms could be easily integrated into the model by modifying the value (payoff) function, similar to such approaches in existing frameworks on inequity aversion or betrayal aversion. Likewise, extending the model to multiple-player scenarios would be a straightforward adaptation, requiring only modifications to allow for additional players in the recursive reasoning structure. Ecological validity is a general concern for lab-based experiments. However, we believe that using a simple, well-known game like Rock-Paper-Scissors—widely played across different cultures and age groups—enhances the ecological validity of our behavioral paradigm. Similarly, we speculate that the neural signature identified in this study could be easily adapted to other types of task-based fMRI studies. Specifically, an exciting avenue for future research would be to test whether this neural signature generalizes across different social contexts, such as trust games or even

tasks involving emotional inference of mental states. Examining how this neural pattern manifests in diverse strategic and non-strategic social interactions could provide deeper insights into the neural basis of mentalization.

Potential extensions for the CHASE model. One strength of the formalism for belief updating that we introduce and validate here is that it is relatively simple, meaning that it is generic and stable across different opponents. However, CHASE is also flexible enough that it may in the future be extended to incorporate more detailed (but also complex) assumptions. First, it would be interesting to assess to what extent the model can be adapted to capture behaviour in games with a different structure than the RPS games we tested here. While this technically only requires adapting i) what governs unstrategic play and ii) the best-response function, practically it needs to be assessed for different games if the resulting level-specific behavioural predictions are distinct enough to allow for successful learning, as demonstrated here for RPS. Such an adaptation might also entail incorporating more complex social inference components, such as inference over parameters other than the level or multiple step forward planning (e.g., potentially leading to strategic manipulation and effects towards the end of an interaction in non-zero sum games where constant exploitation leads to worse outcomes, unlike the RPS game under study here). Second, while the model updates beliefs about the other's sophistication, it assumes that the other is endowed with a static level; this could be extended to also incorporate belief updates about belief-updating opponents (but note that this would only change predictions for $\kappa \geq 3$, as $\kappa = 2$ agents assume the opponent is $\kappa = 1$ and therefore doesn't update beliefs). Third, it is possible that participants hold non-uniform (initial) priors about others' levels, or update priors based on experience from interacting with previous opponents. Future work could test for all these more complex model extensions, which would probably require increasing the number of opponents to robustly estimate such possible intricacies of the belief-updating process.

Comparison to previous empirical results of level *k* reasoning. In line with previous work, we found that not all participants were able to compete with the most sophisticated artificial opponents, indicating a ceiling effect on their strategic sophistication. This suggests that despite its flexibility, the mentalization process may be bounded in its complexity by individual factors. However, in contrast to previous accounts, most participants in our study were able to reach level 3 (and potentially even higher, which was not investigated here). A potential reason for this discrepancy is that static (typically one-shot) approaches for eliciting

mentalization may have provided only a snapshot of this ability that may have been confounded by initial beliefs. Accordingly, it is unclear whether participants could have adapted to even higher levels than investigated here with sufficient time and practice. In line with this, it has been shown that in dominance-solvable games participants can quickly adapt to even higher levels, but this may be confounded by an overlap of high-level strategies and optimal play as prescribed by game theory (i.e. Nash equilibrium). Future studies in economics and psychology should therefore account for the belief updating process, as in the CHASE model, to disentangle equilibrium strategies from actual adjustments in mentalization depth.

Implication for related computational models of mentalization. Our results also provide important information concerning the question of which information participants may use to form beliefs about their opponents' behavior. While many previous models addressing this question have assumed recursive reasoning (i.e. "I think that you think that I …"), what assumptions participants hold to predict the behavior of the least sophisticated agent (i.e. level-0 behavior that all higher levels respond to) is often arbitrarily determined by the experimenter. This question is especially challenging in the context of repeated interactions. Here, we address this challenge by means of empirical model comparison. In particular, we tested a range of plausible level-0 behaviors proposed in the literature and found strong evidence that players track their opponent's action frequencies, rather than rewards (of picking a particular action) or desired counterfactuals. While this comparison was conclusive in all datasets employing our artificial opponents, in human-human interactions we also found some evidence for reward-learning. As we show that these two strategies can be disentangled unambiguously by our model, future work investigating the complexities of natural interactions (rather than the more controlled setup employed here) might benefit from the possibility to empirically investigate this variability.

Our results also inform the nature of the best response, given one's belief about the opponent's sophistication. In particular, subjects seem to base their response not on a single level (or belief), as was suggested in a series of models of level-*k* reasoning, but rather on the distribution of beliefs, as suggested by cognitive hierarchy approaches. Accordingly, our findings substantially narrow down the modeling space for future studies of repeated interactions in mixed strategy games. Note that in this aspect, our model converges with other prominent computational models, such as the influence model (Hampton et al, 2008), which also capture (implicitly) recursive steps of reasoning. However, the influence model is

restricted to two-action games and is limited to lower levels of reasoning (levels 1 and 2), so that a direct comparison of the two models is not feasible for RPS games with three or more responses as used here. Nevertheless, it would be an interesting avenue for future work to examine how both models perform in simpler two-action games, such as matching pennies.

Clinical applications. Our results also have implications for clinical studies of patient populations that are typically characterized by impaired mentalization abilities. The approach we introduce here provides an effective computational tool to assess adaptive mentalization capabilities: A lack of adaptation to the other's strategy inevitably will lead to a quantifiable decrease in behavioral success, since our task provides a standardized strategic interaction for which a failure to mentalize clearly maps to measurable decreases in win rates. This, as well as the neural marker specifically for assessing the adaptiveness of mentalization, may help to diagnose and understand the social interaction and mentalization difficulties often observed in certain mental disorders and neurodivergent phenotypes, like autism spectrum disorder (ASD) or borderline personality disorder (BPD). The CHASE model and its implementation may be particularly suited for such clinical applications, since it offers a standardized approach to investigate mentalization processes across various contexts, treatments, and clinical populations. It allows the experimenter to exercise full control over the strategic setting, by integrating artificial opponents that are indistinguishable from human opponents. This offers not only logistical advantages (less complex experimental setups and lower experimental costs), but also full experimental control over the opponent's behavior. This opens the door for targeted investigation of deficits in both specific mentalization strategies (e.g., level-2 play) as well as the ability to recognize and adapt to changes in the behavior of interaction partners, thereby offering a much more realistic model of the multi-faceted demands of real-life mentalization than existing clinical diagnosis tools.

References

1. Camerer, C. & Hua Ho, T. Experience-weighted attraction learning in normal form games. *Econometrica* **67**, 827–874 (1999).
2. Palminteri, S., Wyart, V. & Koechlin, E. The Importance of Falsification in Computational Cognitive Modeling. *Trends Cogn Sci* **21**, 425–433 (2017).
3. Esteban, O. *et al.* fMRIPrep: a robust preprocessing pipeline for functional MRI. *Nat. Methods* **16**, 111–116 (2019).
4. Gorgolewski, K. *et al.* Nipype: a flexible, lightweight and extensible neuroimaging data processing framework in python. *Front. Neuroinform.* **5**, 13 (2011).
5. Tustison, N. J. *et al.* N4ITK: improved N3 bias correction. *IEEE Trans. Med. Imaging* **29**, 1310–1320 (2010).
6. Avants, B. B., Epstein, C. L., Grossman, M. & Gee, J. C. Symmetric diffeomorphic image registration with cross-correlation: evaluating automated labeling of elderly and neurodegenerative brain. *Med. Image Anal.* **12**, 26–41 (2008).
7. Zhang, Y., Brady, M. & Smith, S. Segmentation of brain MR images through a hidden Markov random field model and the expectation-maximization algorithm. *IEEE Trans. Med. Imaging* **20**, 45–57 (2001).
8. Dale, A. M., Fischl, B. & Sereno, M. I. Cortical surface-based analysis. I. Segmentation and surface reconstruction. *Neuroimage* **9**, 179–194 (1999).
9. Klein, A. *et al.* Mindboggling morphometry of human brains. *PLoS Comput. Biol.* **13**, e1005350 (2017).
10. Fonov, V. S., Evans, A. C., McKinstry, R. C., Almli, C. R. & Collins, D. L. Unbiased nonlinear average age-appropriate brain templates from birth to adulthood. *Neuroimage* **Supplement 1**, S102 (2009).
11. Cox, R. W. & Hyde, J. S. Software tools for analysis and visualization of fMRI data. *NMR Biomed.* **10**, 171–178 (1997).
12. Greve, D. N. & Fischl, B. Accurate and robust brain image alignment using boundary-based registration. *Neuroimage* **48**, 63–72 (2009).
13. Jenkinson, M., Bannister, P., Brady, M. & Smith, S. Improved optimization for the robust and accurate linear registration and motion correction of brain images.

Neuroimage **17**, 825–841 (2002).

14. Lanczos, C. Evaluation of Noisy Data. *Journal of the Society for Industrial and Applied Mathematics Series B Numerical Analysis* **1**, 76–85 (1964).
15. Abraham, A. *et al.* Machine learning for neuroimaging with scikit-learn. *Front. Neuroinform.* **8**, 14 (2014).
16. Hebart, M. N., Görgen, K. & Haynes, J.-D. The Decoding Toolbox (TDT): a versatile software package for multivariate analyses of functional imaging data. *Front. Neuroinform.* **8**, 108528 (2015).
