# Supplementary material for: A neural signature of adaptive mentalization
Source: Nat Neurosci. 2026 Mar 9;29(4):934–44. doi: 10.1038/s41593-026-02219-x (PMC13061600; doi:10.1038/s41593-026-02219-x)
Supplement: Supplementary file 2 — Reporting Summary [file 41593_2026_2219_MOESM2_ESM.pdf]

nature portfolio

Corresponding author(s): Niklas Buergi, Gökhan Aydogan, Christian Ruff

Last updated by author(s): 12.11.2025

Reporting Summary

Nature Portfolio wishes to improve the reproducibility of the work that we publish. This form provides structure for consistency and transparency in reporting. For further information on Nature Portfolio policies, see our Editorial Policies and the Editorial Policy Checklist.

Statistics

For all statistical analyses, confirm that the following items are present in the figure legend, table legend, main text, or Methods section.

n/a	Confirmed	
☐	☒	The exact sample size (*n*) for each experimental group/condition, given as a discrete number and unit of measurement
☐	☒	A statement on whether measurements were taken from distinct samples or whether the same sample was measured repeatedly
☐	☒	The statistical test(s) used AND whether they are one- or two-sided<br>*Only common tests should be described solely by name; describe more complex techniques in the Methods section.*
☐	☒	A description of all covariates tested
☐	☒	A description of any assumptions or corrections, such as tests of normality and adjustment for multiple comparisons
☐	☒	A full description of the statistical parameters including central tendency (e.g. means) or other basic estimates (e.g. regression coefficient) AND variation (e.g. standard deviation) or associated estimates of uncertainty (e.g. confidence intervals)
☐	☒	For null hypothesis testing, the test statistic (e.g. *F*, *t*, *r*) with confidence intervals, effect sizes, degrees of freedom and *P* value noted<br>*Give P values as exact values whenever suitable.*
☒	☐	For Bayesian analysis, information on the choice of priors and Markov chain Monte Carlo settings
☐	☒	For hierarchical and complex designs, identification of the appropriate level for tests and full reporting of outcomes
☐	☒	Estimates of effect sizes (e.g. Cohen's *d*, Pearson's *r*), indicating how they were calculated

Our web collection on statistics for biologists contains articles on many of the points above.

Software and code

Policy information about availability of computer code

Data collection	Stimulus presentation and experimental control were implemented using Psychtoolbox (MATLAB). The toolbox was used to generate and perform the interactive task with precise timing. No custom hardware was used beyond standard laboratory equipment. The code used to generate the stimuli is available upon request.
Data analysis	The analysis code to produce all main results and figures can be accessed at: https://github.com/ruffgroup/neural_signature_of_mentalization. The analysis was performed using Matlab R2023b, in addition to the following external toolboxes: VBA toolbox (5899497), SPM12 (7771) and SnPM13 (13.1.09), The Decoding Toolbox (3.999G), CanlabCore (d0122bc), MRIcroGL (1.2.20220720), and AAL (v4).

For manuscripts utilizing custom algorithms or software that are central to the research but not yet described in published literature, software must be made available to editors and reviewers. We strongly encourage code deposition in a community repository (e.g. GitHub). See the Nature Portfolio guidelines for submitting code & software for further information.

Data

Policy information about availability of data

All manuscripts must include a data availability statement. This statement should provide the following information, where applicable:

- Accession codes, unique identifiers, or web links for publicly available datasets
- A description of any restrictions on data availability
- For clinical datasets or third party data, please ensure that the statement adheres to our policy

All behavioral data can be accessed at: https://github.com/ruffgroup/neural_signature_of_mentalization. Preprocessed neural data is available upon request.

Research involving human participants, their data, or biological material

Policy information about studies with human participants or human data. See also policy information about sex, gender (identity/presentation), and sexual orientation and race, ethnicity and racism.

Reporting on sex and gender	Sex and gender were not the primary focus of this study and were therefore not specifically analyzed, but self-reported gender was used as a control variable in some of the behavioral analyses. We recruited 506 healthy participants from the subject pool of the Department of Economics at the University of Zurich. Fifty of these completed the task while undergoing functional magnetic resonance imaging (fMRI; all males, age = 23.44 ± 2.57), the others only performed a behavioral version of the task (56% females, age = 24.7 ± 4.7). To replicate our neural results in a more diverse sample, we also recruited another sample of 47 participants (N = 47, 57% female, age 32 ± 8.2, years of education 16 ± 3.1).
Reporting on race, ethnicity, or other socially relevant groupings	Socially relevant group differences were not the primary focus of this study and were therefore not specifically elicited or analyzed.
Population characteristics	Population characteristics were not the primary focus of this study and were therefore not further analyzed.
Recruitment	Participants were recruited using the Online Recruitment System for Economic Experiments (ORSEE) from local universities in Zurich for the main study, and via online ads for the replication study.
Ethics oversight	The study was reviewed and approved by the Kantonale Ethikkommission Zürich (KEK).

Note that full information on the approval of the study protocol must also be provided in the manuscript.

Field-specific reporting

Please select the one below that is the best fit for your research. If you are not sure, read the appropriate sections before making your selection.

☐ Life sciences ☒ Behavioural & social sciences ☐ Ecological, evolutionary & environmental sciences

For a reference copy of the document with all sections, see nature.com/documents/nr-reporting-summary-flat.pdf

Behavioural & social sciences study design

All studies must disclose on these points even when the disclosure is negative.

Study description	Experimental study using quantitative methods, drawing on principles of computational neuroscience to investigate behavior under controlled conditions.
Research sample	The study sample consisted of (non-representative) university student populations recruited from local universities in Zurich. We recruited 506 healthy participants from the subject pool of the Department of Economics at the University of Zurich. Fifty of these completed the task while undergoing functional magnetic resonance imaging (fMRI; all males, age = 23.44 ± 2.57), the others only performed a behavioral version of the task (56% females, age = 24.7 ± 4.7). To replicate our neural results in a more diverse (non-representative) sample, we also recruited another sample of 47 participants (N = 47, 57% female, age 32 ± 8.2, years of education 16 ± 3.1) from the general population in Zurich and surrounding area.
Sampling strategy	The sample size was predetermined based on effect size estimates from previous publications (e.g. Hill et al 2017), ensuring adequate statistical power to test the main hypotheses.
Data collection	Data were collected in a computerized task conducted during functional magnetic resonance imaging (fMRI). Stimulus presentation and task control were computer-based (using PsychToolbox in Matlab), and responses were recorded digitally.
Timing	The behavioral data was collected between February 2019 and July 2021, the neural data was collected between October 2021 and July 2022.

Data exclusions	Test Sample: To ensure that all participants indeed engaged in strategizing and did not just resort to random gameplay, we assessed their behavior against the artificial opponent of the lowest sophistication (k = 0). Since this opponent shows a simple tendency to repeat past actions, any attentive player should be able to successfully adapt to them over time. Accordingly, participants who did not perform above chance against this opponent were excluded from the analysis; this affected only 2 participants, leaving a final sample of N = 48.<br>Replication Sample: To test the robustness of our main findings, we repeated the corresponding neural analyses in an independent sample (N = 47; see Participants). Data exclusion criteria (see above) were met by only one participant, leaving a final sample of N = 46.
Non-participation	No participants dropped out.
Randomization	Participants played against three different opponent types in the scanner and the order of opponent types was randomized and counterbalanced across runs.

Reporting for specific materials, systems and methods

We require information from authors about some types of materials, experimental systems and methods used in many studies. Here, indicate whether each material, system or method listed is relevant to your study. If you are not sure if a list item applies to your research, read the appropriate section before selecting a response.

Materials & experimental systems

n/a	Involved in the study	
☒	☐	Antibodies
☒	☐	Eukaryotic cell lines
☒	☐	Palaeontology and archaeology
☒	☐	Animals and other organisms
☒	☐	Clinical data
☒	☐	Dual use research of concern
☒	☐	Plants

Methods

n/a	Involved in the study	
☒	☐	ChIP-seq
☒	☐	Flow cytometry
☐	☒	MRI-based neuroimaging

Plants

Seed stocks	n/a
Novel plant genotypes	n/a
Authentication	n/a

Magnetic resonance imaging

Experimental design

Design type	Experimental study using a event-related task-based fMRI design, with computerized stimulus presentation and behavioral response recording.
Design specifications	In the discovery fMRI data set, participants were scanned 6 runs in total. One run consisted of 40 trials, while each trial consisted of a fixation phase (0 - 6 secs), a response phase (3 - 6 secs) and feedback phase (2 secs). Inter trial intervals (ITIs) were carefully chosen via simulations to maximize design efficiency and to de-correlate response from feedback phase. On average, trials lasted around 7.3 secs, with a total of 24 minutes of the game inside the fMRI scanner.<br>For the replication fMRI data set, we collected more data by increasing the number of observations per subject (instead of scanning more participants), and thus changed the experimental timing in the replication fMRI data set as follows. Participants were scanned 9 runs in total. One run consisted of 40 trials, while each trial consisted of a fixation phase (1 - 3 secs), a response phase (3 - 5 secs) and feedback phase (2 secs). Again, ITIs were carefully chosen via simulation to maximize design efficiency and to de-correlate response from feedback phase. On average, trials lasted around 7.3 secs, with a total of about 45 minutes of the game inside the scanner.
Behavioral performance measures	Participants played variants of repeated Rock-Paper-Scissors (RPS) games against artificial opponents. In every round, both players simultaneously picked one out of three available actions (“rock”, “paper”, or “scissors”). Each action beat one action and in turn got beaten by the third, leading to a circular, non-transitive dominance structure (i.e. “rock”

beats “scissors”, “scissors” beats “paper”, “paper” beats “rock”). The player who picked the number that was exactly one step ahead of the opponent’s number won the round (and the other player lost; choosing the same action resulted in a tie; the direction of the action dominance was indicated by arrows).

Acquisition

Imaging type(s): Functional and structural

Field strength: 3T

Sequence & imaging parameters: While participants performed the task in the scanner, we acquired T2*-weighted whole-brain echo planar images using a Philips Achieva 3 T whole-body scanner (Philips Medical Systems) equipped with an 8-channel Philips sensitivity-encoded (SENSE) head coil. We used a TR of 2238 ms and TE of 30 ms with 40 slices (transversal, ascending acquisition); 3 mm slice thickness; 3 mm x 3 mm in-plane resolution; 0.5 mm gap; 90° flip angle. Five dummy-image excitations were performed and discarded before functional image acquisition started. Additionally, we acquired a high-resolution T1-weighted 3D fast-field echo structural scan used for image registration during post-processing (sequence parameters: 170 sagittal slices; matrix size: 256 x 256; voxel size: 1 x 1 x 1 mm; TR/TE: 8.3/3.9 ms). Additionally, we recorded physiological data during scanning to control for heart and breathing artefacts.

Area of acquisition: Whole brain

Diffusion MRI: ☐ Used ☒ Not used

Preprocessing

Preprocessing software: fMRIPrep 20.2.3102, which is based on Nipype 1.6.1103, with standard settings.

Normalization: Volume-based spatial normalization to one standard space (MNI152NLin2009cAsym) was performed through nonlinear registration with antsRegistration (ANTs 2.3.3), using brain-extracted versions of both the T1w reference and the T1w template. The following template was selected for spatial normalization: ICBM 152 Nonlinear Asymmetrical template version 2009c117 [TemplateFlow ID: MNI152NLin2009cAsym].

Normalization template: MNI152NLin2009cAsym

Noise and artifact removal: To control for potential confounds of movement, we added the six motion parameters (three rotations and three translations), their derivatives, and the volume-wise global signal estimate from fMRIPrep as regressors-of-no-interest. We also included 18 regressors based on the software package TAPAS105 (version R2018.1.1) to control statistically for the effects of cardiac and respiratory cycles.

Volume censoring: N/A

Statistical modeling & inference

Model type and settings: Mass univariate analyses were conducted in SPM. At the first level, we implemented a mass-univariate general linear model (GLM) in which the behaviorally derived variables (e.g. belief update) were entered as a parametric modulator and regressed against the BOLD signal at each voxel across the whole brain. Second-level analyses were performed using a random-effects model in SPM.
Multivariate Analysis: We employed established toolboxes to perform multivariate decoding, using default parameter settings without any hyper-parameter tuning to avoid the risk of overfitting (which is inherent in the limited training data sizes feasible with task-based fMRI).
For the categorical level decoding, we used Support Vector Machines (SVM) as implemented in The Decoding Toolbox (TDT110). In the background, this toolbox relies on the libsvm library and defaults to the standard L2-regularized linear C-SVC formalization, where the level of regularization is determined by a parameter c that is set to 1. No prior data reduction step is applied. While this in principle could result in a higher risk of overfitting, our procedure of training across subjects and evaluating the performance on held-out subjects ensures that any overfitting would - if anything - decrease the accuracy, suggesting that our results constitute a lower bound on the possible decoding accuracy.
For the continuous belief update decoding, we used the LASSO-PCR algorithm as implemented by the canlab toolbox (https://github.com/canlab/CanlabCore).

Effect(s) tested: Univariate analyses: At the group (second) level, we tested for brain regions in which BOLD activity scaled with the behaviorally derived parametric modulators. Whole-brain and ROI-based mass-univariate tests were performed across relevant voxels, and significance was assessed using cluster-level family-wise error (FWE) correction.
Multivariate analyses: We used principal components regression with LASSO regularization (PCR-LASSO), as implemented in the method developed by Wager and colleagues, to predict the behaviorally derived parametric modulators from whole-brain activity patterns.

Specify type of analysis: ☐ Whole brain ☐ ROI-based ☒ Both

Anatomical location(s)	We restricted the analyses to a set of a priori selected regions-of-interest (ROIs) based on an automated meta-analysis for the term "theory [of] mind" (using Neurosynth's uniformity test with the default p(FDR) < .01 cutoff; including 181 studies as of September 16th, 2022). We only kept connected clusters of size k > 50 to remove noise from the mask.
Statistic type for inference (See Eklund et al. 2016)	Cluster-based inference
Correction	FWE

Models & analysis

n/a	Involved in the study
☐	☒ Functional and/or effective connectivity
☒	☐ Graph analysis
☐	☒ Multivariate modeling or predictive analysis

Functional and/or effective connectivity	Functional connectivity strength was represented by Fisher-transformed bivariate correlation coefficients from a general linear model (weighted-GLM), estimated separately for each pair of ROIs, characterizing the association between their BOLD signal time series.
Multivariate modeling and predictive analysis	We used a multivariate pattern analysis approach (MVPA) to test if the levels of strategic sophistication as well as the extent of the associated belief update can be decoded and predicted out-of-sample from neural activity. For the former, we used Support Vector Machines as implemented in The Decoding Toolbox (for technical details, see the Supplementary Materials). This was based on average run-level activity during either the choice or the feedback phases of the game. We fitted first-level models where we either included all trials within a run (and linked it to the level that was predominantly played during this run), or only trials where behavior can be linked to a particular level with certainty (based on a permutation distribution; we also added regressors for motion and physiological noise correction).<br>For the continuous belief update decoding, we employed Lasso-PCR (least absolute shrinkage and selection operator principal component regression) as implemented in the canlab toolbox (https://github.com/canlab/CanlabCore; for technical details, see the Supplementary Materials). We fitted another set of first-level models where trials are assigned to one of five separate regressors according to the extent of the BU (using equally spaced subject-specific bins of the log-transformed time-series; again including regressors for noise correction). The resulting beta maps were then averaged across runs, giving one beta map per bin for each subject. To evaluate the model whilst minimizing the risk of over-fitting, we performed a leave-one-subject-out cross-validation scheme for both types of decoding (i.e. training the classifier on all but one subjects and evaluating it on the left-out one), and performed permutation testing to compute non-parametric p-values. For the categorical level decoding, we report balanced accuracy scores. For the continuous belief update decoding, we report both average (using Fisher's z-transformation) and overall (i.e. pooled across subjects) Pearson correlation coefficients between the computational model-inferred extent of the belief update and the one predicted from neural activation patterns.
